# Supplementary material for: Non-invasive assessment of normal and impaired iron homeostasis in the brain
Source: Nat Commun. 2023 Sep 12;14:5467. doi: 10.1038/s41467-023-40999-z (PMC10497590; doi:10.1038/s41467-023-40999-z)
Supplement: Supplementary file 1 — Supplementary Information [file 41467_2023_40999_MOESM1_ESM.pdf]

Supplementary Materials for

# "Non-invasive assessment of normal and impaired iron homeostasis in the brain"

# Contents

Supplementary Section 1 .... 3
Supplementary Figure 1 .... 4
Supplementary Section 2 (Supplementary Figures 2,3) .... 5
Supplementary Section 3 (Supplementary Figure 4) .... 8
Supplementary Figure 5 .... 10
Supplementary Section 4 (Supplementary Figures 6-8) .... 11
Supplementary Figure 9 .... 15
Supplementary Section 5 (Supplementary Figure 10) .... 15
Supplementary Section 6 (Supplementary Figures 11-13) .... 18
Supplementary Figure 14 .... 23
Supplementary Section 7 (Supplementary Figures 15-16) .... 23
Supplementary Section 8 (Supplementary Figures 17-19) .... 27
Supplementary Figure 20 .... 31
Supplementary Figure 21 .... 32
Supplementary Figure 22 .... 32
Supplementary Section 9 (Supplementary Figure 23-31) .... 33
Supplementary Section 10 (Supplementary Figures 32-34) .... 43
Supplementary Section 11 (Supplementary Figures 35-37) .... 45
Supplementary Table 2 .... 47
Supplementary Figure 38 .... 47
Supplementary Section 12 (Supplementary Figure 39) .... 48
Supplementary Table 3 .... 49
Supplementary Figure 40 .... 50
Supplementary Table 4 .... 51
Supplementary Figure 41 .... 52
Supplementary methods .... 53
Supplementary references .... 53

# Supplementary Section 1

## The theoretical basis for the r1-r2* relaxivity of brain tissue

Brain tissue contains a complex milieu of iron compounds with variable iron binding capacities and aggregation states and includes myelin. Here we will expand the biophysical model of the $r_1$-$r_2^*$ relaxivity ("*In vivo* iron relaxivity model" in Methods) for the case of a heterogenous iron environment and in the presence of myelin.

Assuming the iron environment of a myelinated tissue contains N different iron compounds, and under the assumption that water can freely diffuse, the MR relaxation rates can be expressed as[1,2]:

S1) $$R_1 = \sum_N r_{(1,i)}[i] + r_{(1,M)}[M] + C_1$$

S2) $$R_2^* = \sum_N r_{(2,i)}[i] + r_{(2,M)}[M] + C_2$$

Where $[i]$ is the concentration of the i'th iron compound, and $[M]$ is the myelin concentration. $r_{(1,i)}$ and $r_{(2,i)}$ are the $R_1$- and $R_2^*$- relaxivities of the i'th iron compound. $r_{(1,M)}$ and $r_{(2,M)}$ are the $R_1$- and $R_2^*$-relaxivities of myelin. $C_1$ and $C_2$ are constants which capture other non-iron and non-myelin contributions.

The $r_1$-$r_2^*$ relaxivity measurement is defined as the linear dependency of $R_1$ on $R_2^*$ (within an ROI in the brain, across *in vitro* samples or across each voxel's local neighborhood). This is equivalent to the total change in $R_1$ values relative to the total change in $R_2^*$ values ($\frac{\Delta R_1}{\Delta R_2^*}$):

S3) $$\frac{\Delta R_1}{\Delta R_2^*} = \frac{\sum_N r_{(1,i)}[\Delta i] + r_{(1,M)}[\Delta M]}{\sum_N r_{(2,i)}[\Delta i] + r_{(2,M)}[\Delta M]}$$

Where $[\Delta i]$ and $[\Delta M]$ are the changes in the iron compounds and myelin concentrations within an ROI respectively.

## Supplementary Figure 1

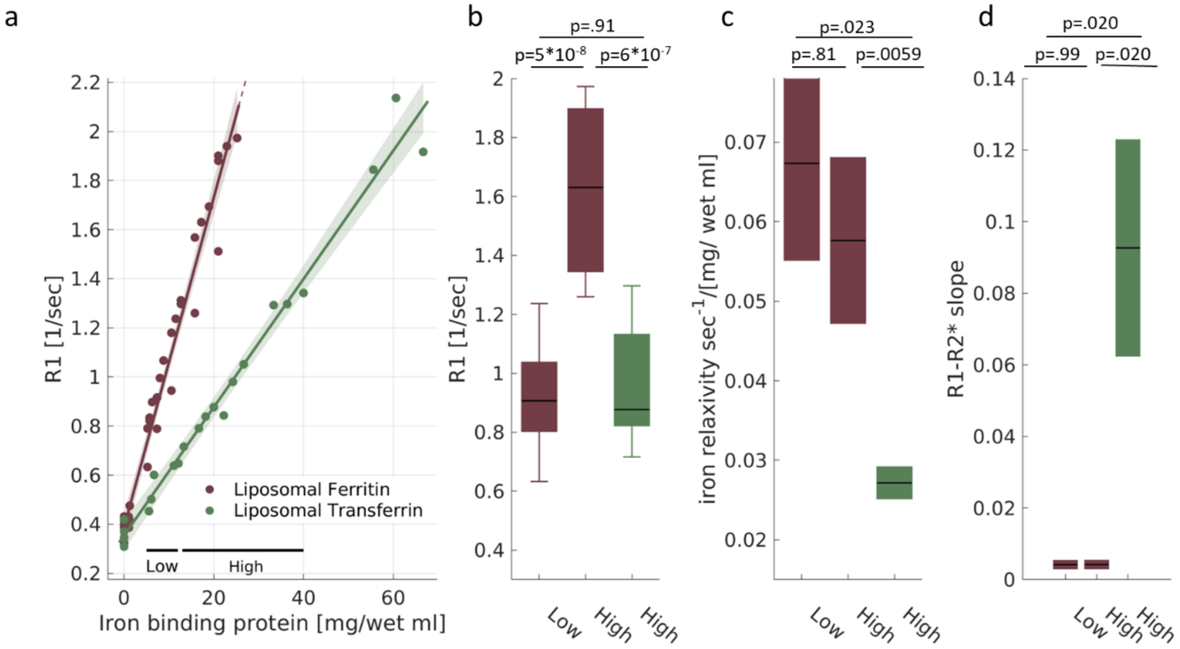


***Sup. Figure 1: The effect of iron concentration on different MR estimations. (a)*** *The dependency of $R_1$ on the iron-binding protein concentration for liposomal ferritin and liposomal transferrin. Data points represent biologically independent liposomal samples with varying iron-binding protein concentrations relative to the water fraction ([mg/wet ml]), showing median values for each sample. The linear relationships between relaxation rates and iron-binding protein concentrations are marked by solid lines. The slopes of these lines are the iron relaxivities. Shaded areas represent the 95% confidence bounds.* ***(b)*** *The ambiguity in $R_1$; $R_1$ changes as a function of both iron environment and iron concentration. This is shown by calculating the median $R_1$ value over biologically independent samples with high and low iron-binding protein concentrations (marked in (a); concentration ranges were chosen so that the number of data points in each range is relatively similar; N(low ferritin)=12), N(high ferritin)=11, N(high transferrin)=9). We find that $R_1$ is greater for a higher ferritin concentration than for a lower ferritin concentration, but also find that $R_1$ is greater for ferritin than for transferrin. For each box, the central line marks the median, the box extends vertically between the 25th and 75th percentiles, and the whiskers extend to the most extreme data points.* ***(c-d)*** *The ambiguity in $R_1$ is resolved by the $R_1$-iron relaxivity (c) and the $r_1$-$r_2^*$ (d), which are consistent when computed over higher or lower ferritin concentrations, and are consistently different from the iron relaxivity of transferrin regardless the concentration. N(low ferritin)=12), N(high ferritin)=11, N(high transferrin)=9, biologically independent samples. For each box, the central lines marks the iron relaxivity, and the box shows the 95% confidence bounds of the linear fit. p-values are for the one-way ANCOVA test corrected for multiple comparisons.*

# Supplementary Section 2 (Supplementary Figures 2,3)

## The dependency of R1 and R2* on the iron concentration.

In Figure 2 we computed the iron relaxivity as the dependency of $R_1$ and $R_2^*$ on the concentration of iron-binding proteins. We showed that different iron environments have different relaxivities. However, different proteins bind different amounts of iron. For example, ferritin binds three orders of magnitude more iron ions than does transferrin[3]. Therefore, we wanted to exclude the possibility that the different iron ion concentrations drive the different relaxivities of ferritin and transferrin.

We verified that the relaxivity changes according to the molecular type of iron, even when accounting for discrepancies in iron loading. We estimated the iron ion concentrations for ferritin and transferrin (see Methods section "Estimation of total iron content in phantoms”) and tested whether those values can explain their different iron relaxivities. Importantly, after computing the iron relaxivity as the dependency of relaxation rates on the iron ion concentrations (rather than the concentration of iron-binding proteins), we still find that different iron environments have distinct relaxivities (Sup. Figure 2, p(one-sided ANCOVA)$<10^{-39}$).

To further stress the sensitivity of the $r_1$-$r_2^*$ relaxivity to the iron environment, we compared the relaxivity of liposomal ferrous iron ($Fe^{2+}$) and iron bound to liposomal transferrin (Sup. Figure 3). Unlike ferritin and transferrin, these two iron compounds have relatively similar iron ion concentrations. Yet we find that they produce different iron relaxivities (p(one-sided ANCOVA)$=1.3*10^{-13}$ & $1.4*10^{-5}$ for R1 and R2* respectively). The $r_1$-$r_2^*$ relaxivities of these two iron environments are different as well (p(one-sided ANCOVA)=0.027). Therefore, the iron relaxivity and the $r_1$-$r_2^*$ relaxivity are changing as a function of the molecular  iron environment, even when accounting for the differences in iron binding.

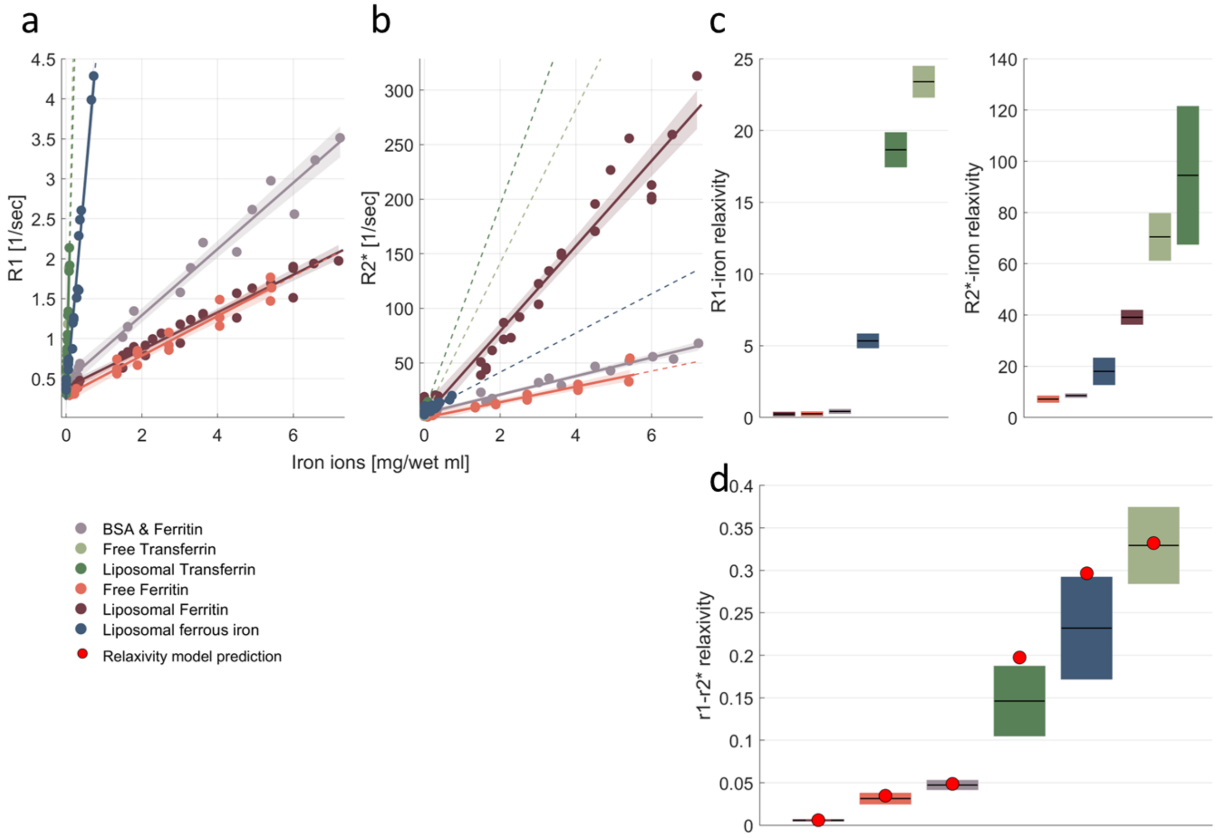


***Sup. Figure 2: The iron relaxivity and the $r_1$-$r_2^*$ relaxivity are sensitive to the molecular type of iron regardless of the differences in iron-binding. (a-b)*** *The dependency of $R_1$ and $R_2^*$ on the estimated iron concentration (see method section "Estimation of total iron content in phantoms") for six different iron compounds free ferritin (N=20), liposomal ferritin (N=36), bovine serum albumin (BSA)-ferritin mixture (N=22), free transferrin (N=6), liposomal transferrin (N=22) and liposomal ferrous iron (N=20). Data points represent median values of biologically independent samples with varied estimated iron ion concentrations relative to the water fraction ([mg/wet ml]). The linear relationships between relaxation rates and iron concentration are marked by lines. The slopes of these lines are the iron relaxivities. Dashed lines represent extrapolations of the linear fits, and shaded areas represent the 95% confidence bounds.* ***(c)*** *The iron relaxivities of $R_1$ and $R_2^*$ are different for different iron environments (p(one-sided ANCOVA)<$10^{39}$). Iron relaxivity is calculated here based on the estimated iron concentration (and not iron-binding proteins concentrations, as in Figure 2). To do so, we use the slope of the linear relationships shown in (a,b), expressed in [sec-1/(mg/wet ml)]. For each box the central lines mark the iron relaxivity, and the box shows the 95% confidence bounds of the linear fit.* ***(d)*** *The theoretical model successfully predicts the $r_1$-$r_2^*$ relaxivity even when it is based on the estimated iron ions concentration (and not iron compound concentrations, as in Figure 2). The model's prediction is based on the ratio between the iron relaxivities of $R_1$ and $R_2^*$ as shown in (c). For each box the central line marks the $r_1$-$r_2^*$ relaxivity, and the box shows the 95% confidence bounds of the linear fit. Red dots represent the prediction of the theoretical model.*

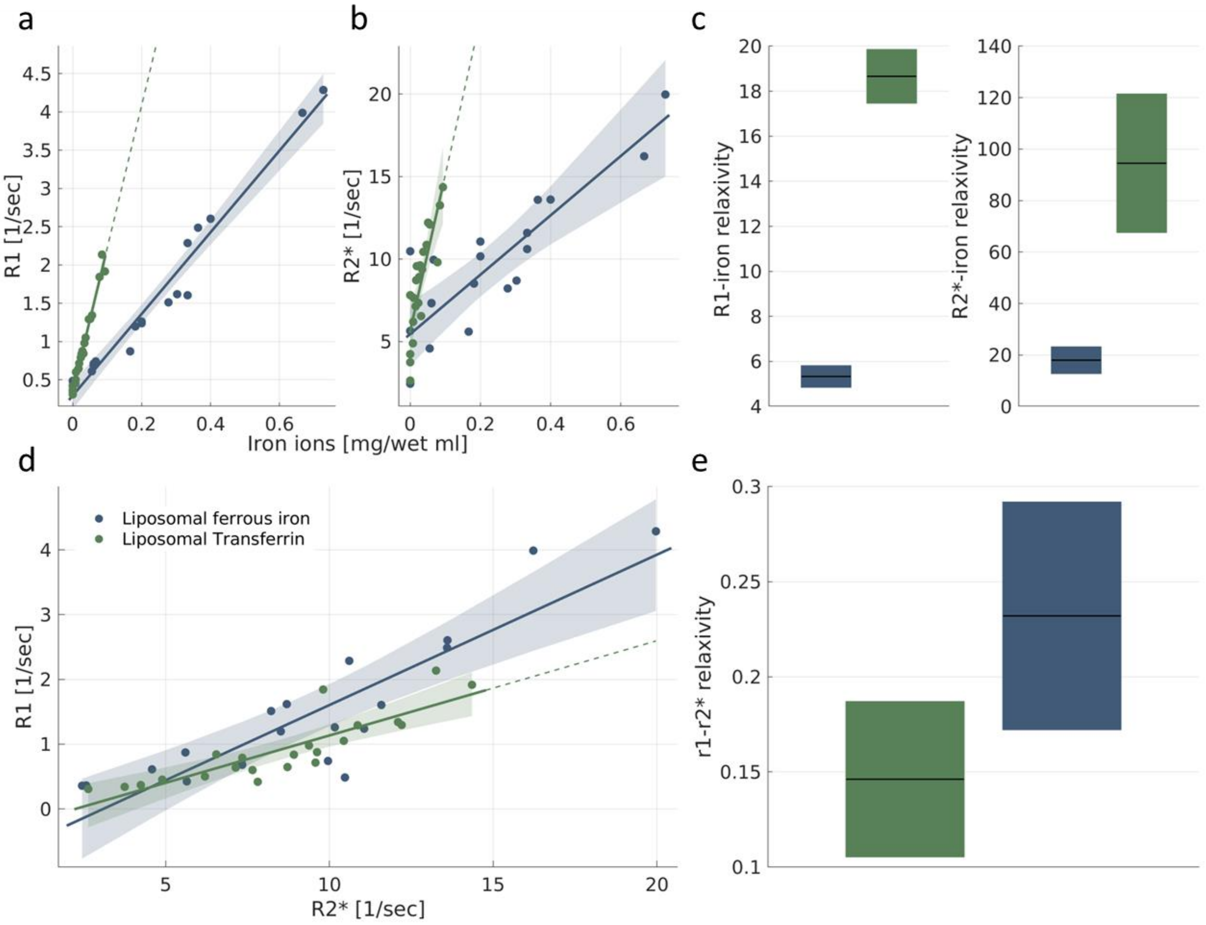


***Sup. Figure 3: The iron relaxivity and the $r_1$-$r_2^*$ relaxivity are sensitive to the molecular iron environment even when the iron concentration is similar. (a-b)*** *The dependency of $R_1$ and $R_2^*$ on the estimated iron concentration for two different iron environments: liposomal transferrin (N=22, purple) and liposomal ferrous iron (N=20, green). Data points represent median values of biologically independent liposomal samples with varying iron ion concentrations relative to the water fraction ([mg/wet ml]). The linear relationships between relaxation rates and iron ion concentration are marked by lines. The slopes of these lines are the iron relaxivities. Dashed lines represent extrapolations of the linear fits. Shaded areas represent the 95% confidence bounds.* ***(c)*** *The iron relaxivity of $R_1$ and $R_2^*$ is different for different iron environments (p(one-sided ANCOVA)=$1.3*10^{-13}$ & $1.4*10^{-5}$ respectively). Iron relaxivity is calculated by taking the slope of the linear relationships shown in (a,b), and is measured in [sec-1/(mg/wet ml)]. For each box, the central line marks the iron relaxivity, and the box shows the 95% confidence bounds of the linear fit.* ***(d)*** *The dependency of $R_1$ on $R_2^*$ for different iron environments. Data points represent median values of biologically independent samples with varying concentrations. The linear relationships between $R_1$ and $R_2^*$ are marked by lines. The slopes of these lines are the $r_1$-$r_2^*$ relaxivities. Dashed lines represent extrapolations of the linear fits. Shaded areas represent the 95% confidence bounds.* ***(e)*** *The $r_1$-$r_2^*$ relaxivity is different for different iron environments (p(one-sided ANCOVA)=0.027). For each box, the central line marks the $r_1$-$r_2^*$ relaxivity, and the box shows the 95% confidence bounds of the linear fit.*

# Supplementary Section 3 (Supplementary Figure 4)

# R2-based relaxivity

Similarly to $R_2^*$, $R_2$ is also sensitive to the effects of iron on the transverse relaxation[3,4]. Theoretically, the dependency of $R_1$ on $R_2$ could also be used as an *in vivo* estimation of the iron relaxivity. To test this, $R_2$ measurements of different iron environments were assessed. This analysis (Sup. Figure 4) shows that different iron environments have distinct $R_2$-iron relaxivity (p(one-sided ANCOVA)=$2.6*10^{-73}$). Comparing the $R_2$-iron relaxivity (Sup. Figure 4) and the $R_2^*$-iron relaxivity (Figure 2c) we find similar contrast between iron environments (e.g. highest relaxivity for ferrous iron and lowest relaxivity for transferrin). This contrast is different from the $R_1$-iron relaxivity. For example, while the $R_1$-iron relaxivity of ferritin is similar for free and liposomal states (Figure 2c), both the $R_2$-iron relaxivity and the $R_2^*$-iron relaxivity are different for liposomal and free ferritin (Sup. Figure 4a-b, Figure 2c). Therefore, these results suggest that $R_2$ could be used instead of $R_2^*$ when calculating the relaxivity. To test this, we estimated the $r_1$-$r_2$ relaxivity for different iron environments (Sup. Figure 4c-d). We found that different iron environments have distinct $r_1$-$r_2$ relaxivity (p(one-sided ANCOVA)=$1.2*10^{-40}$). Moreover, we found great agreement between the experimental results and the theoretical predictions for the $r_1$-$r_2$ relaxivity based on the ratio of iron relaxivities (eq. 2, Sup. Figure 4d). Interestingly, while the theoretical prediction for the $r_1$-$r_2^*$ relaxivity of ferrous iron and liposomal transferrin was a bit higher than the experimental results (Figure 2e), the theoretical prediction of these experimental results is more accurate for the $r_1$-$r_2$ relaxivity. Therefore, we believe that estimating the $r_1$-$r_2$ relaxivity may also be a useful marker of the iron homeostasis. Technically, acquiring $R_1$ and $R_2^*$ with standard MRI protocols can be shorter than acquiring $R_1$ and $R_2$. Both $R_1$ and $R_2^*$ can be estimated from the same widely available sequence (spoiled gradient echo with multiple echoes). However, if measurements of both $R_1$ and $R_2$ are available, we believe that an estimation of the $r_1$-$r_2$ relaxivity also could contribute to the *in vivo* characterization of the iron homeostasis. By joining several iron relaxivity measurements, it might be possible to further disentangle the contributions of different iron environments to the MRI signal.

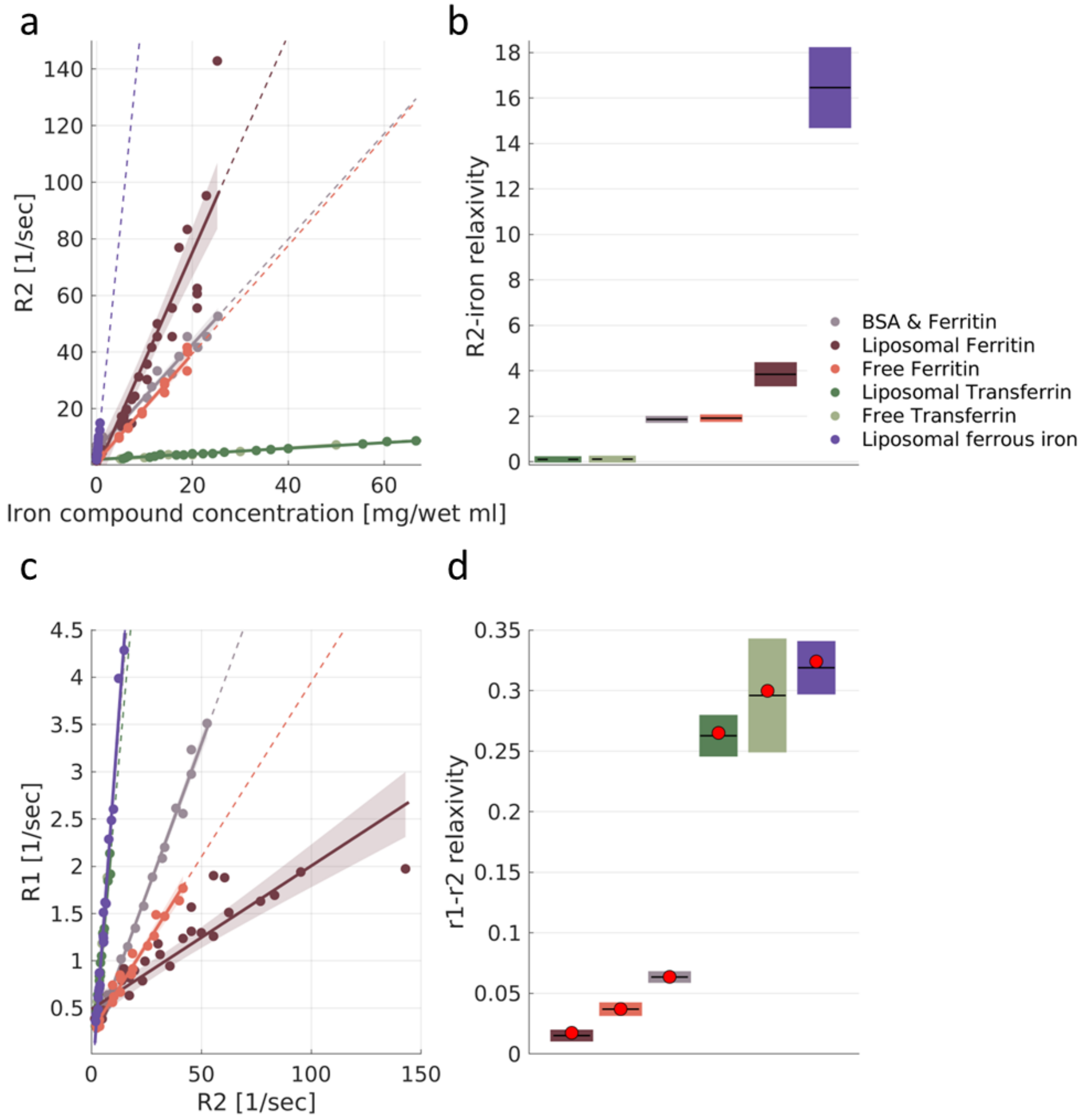


***Sup. Figure 4: The dependency of R1 on R2 for assessing different iron environments. (a)*** *The dependency of R2 on the iron compound concentrations for different iron environments: free ferritin (N=20), liposomal ferritin (N=36), bovine serum albumin (BSA)-ferritin mixture (N=22), free transferrin (N=6), liposomal transferrin (N=22) and liposomal ferrous iron (N=20). Data points represent median values of biologically independent samples with varying concentrations relative to the water fraction ([mg/wet ml]). The linear relationships between relaxation rates and iron- compounds concentrations are marked by lines. We define the slopes of these lines as the iron relaxivities. Dashed lines represent extrapolation of the linear fit. Shaded areas represent the 95% confidence bounds.* ***(b)*** *The iron relaxivity of R2 is different for different iron environments (p(one-sided ANCOVA)=$2.6*10^{-73}$). Iron relaxivities are calculated by taking the slopes of the linear relationships shown in (a,b), and are measured in [$sec^{-1}$/(mg/wet ml)]. For each box, the central mark is the iron relaxivity (slope); the box shows the 95% confidence bounds of the linear fit.* ***(c)*** *The dependency of R1 on R2 for different iron environments. Data points represent median values of biologically independent samples with varying iron compound*

*concentrations relative to the water fraction. The linear relationships of R1 and R2 are marked by lines. The slopes of these lines are the r1-r2 relaxivities. Dashed lines represent extrapolation of the linear fit. Shaded areas represent the 95% confidence bounds.* ***(d)*** *The r1-r2 relaxivities are different for different iron environments (p(one-sided ANCOVA)=1.2*10$^{-40}$). For each box, the central mark is the r1-r2 relaxivity, and the box shows the 95% confidence bounds of the linear fit. Red dots indicate the successful prediction of the experimental r1-r2 relaxivity from the ratio between the iron relaxivities of R1 and R2.*

## Supplementary Figure 5

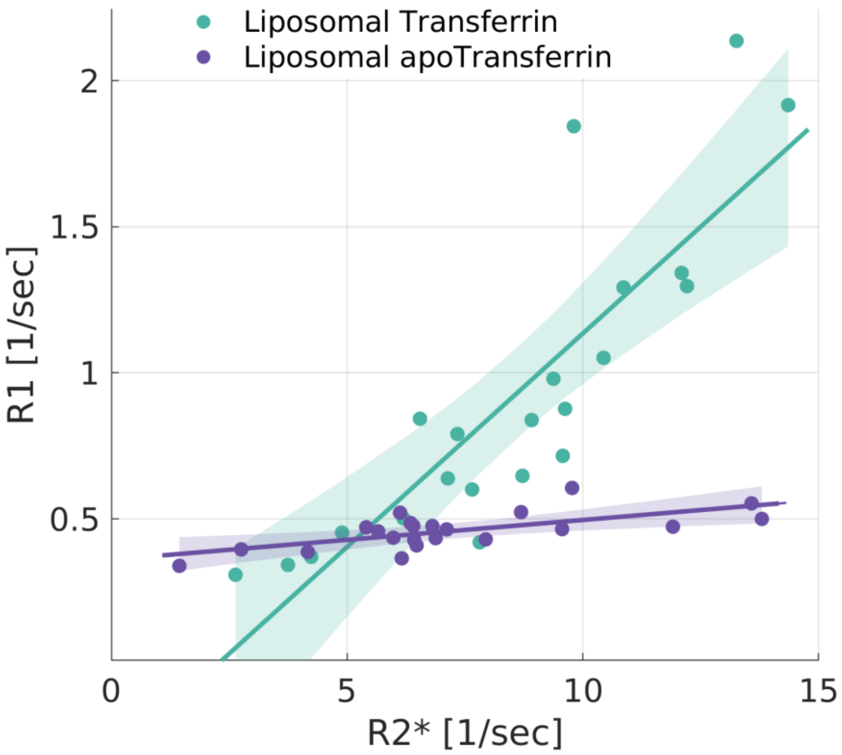


***Sup. Figure 5: Validating the sensitivity of the $r_1$-$r_2^*$ relaxivity to the paramagnetic properties of transferrin.*** *Data points represent the median values of biologically independent liposomal samples with varying concentrations of transferrin (green, N=22) and apo-transferrin (transferrin which is not bound to iron, in purple, N=22). The linear relationships between $R_1$ and $R_2^*$ are marked by solid lines. The slopes of these lines are the $r_1$-$r_2^*$ relaxivities. Shaded areas represent the 95% confidence bounds. Apo-transferrin with no iron has lower $r_1$-$r_2^*$ relaxivity compared to iron-bound transferrin (p(one sided ANCOVA)=5.6*10$^{-8}$). Therefore, the $r_1$-$r_2^*$ relaxivity is sensitive to the paramagnetic properties of iron-binding proteins and not to the proteins themselves.*

# Supplementary Section 4 (Supplementary Figures 6-8)

## The r1-r2* relaxivity of ferritin and transferrin mixtures in vitro.

We tested the theoretical formulation presented in eq. S1-S3 (Sup. Section 1) in an artificial environment of multiple iron compounds. For this aim, we constructed phantom experiments containing both ferritin and transferrin in a liposomal environment. In this synthetic toy model, the transferrin-ferritin fraction ($f = \frac{[Tf]}{[Ft]+[Tf]}$) represents an example for a feature of the molecular iron environment which should affect the $r_1$-$r_2^*$ relaxivity. To test this assumption, the biophysical model shown in Sup. Section 1 can be further developed under the conditions of these phantom experiments:

When ferritin, transferrin and myelin are present, eq. S1-S2 can be expressed as:

S4) $$R_1 = r_{(1,Ft)}[Ft] + r_{(1,Tf)}[Tf] + r_{(1,M)}[M]$$

S5) $$R_2^* = r_{(2,Ft)}[Ft] + r_{(2,Tf)}[Tf] + r_{(2,M)}[M]$$

Where $[Ft]$, $[Tf]$ are the ferritin and transferrin concentrations respectively, measured in [mg/ wet ml]. $[M]$ is the myelin concentration. $r_{(1,Ft)}$, $r_{(1,Tf)}$ and $r_{(1,M)}$ are the $R_1$-relaxivities of ferritin, transferrin and myelin respectively. $r_{(2,Ft)}$, $r_{(2,Tf)}$ and $r_{(2,M)}$ are the $R_2^*$-relaxivities of ferritin, transferrin and myelin respectively. Notably, this model does not depend on the exact units of concentration as long as the relaxivities (rate per concentration) and concentrations measurements agree in units.

According to eq. S3, the $r_1$-$r_2^*$ relaxivity measurement is equivalent to the total change in $R_1$ relative to the total change in $R_2^*$ ($\frac{\Delta R_1}{\Delta R_2^*}$):

S6) $$\frac{\Delta R_1}{\Delta R_2^*} = \frac{r_{(1,Ft)}[\Delta Ft]+r_{(1,Tf)}[\Delta Tf]+r_{(1,M)}[\Delta M]}{r_{(2,Ft)}[\Delta Ft]+r_{(2,Tf)}[\Delta Tf]+r_{(2,M)}[\Delta M]}$$

Where $[\Delta Ft]$, $[\Delta Tf]$, and $[\Delta M]$ are the changes in the ferritin, transferrin and myelin concentrations across *in vitro* samples, respectively.

In the ferritin-transferrin mixtures experiments we tested four different transferrin-ferritin fractions ($f = \frac{[Tf]}{[Ft]+[Tf]}$). For each fraction, we varied ferritin and transferrin concentrations, while keeping the fixed fraction between them (Sup. Figure 6). This allowed us to fit the linear relationship between $R_1$ and $R_2^*$ (the $r_1$-$r_2^*$ relaxivity) for each transferrin-ferritin fraction (Sup. Figure 7).

Assuming the transferrin-ferritin fraction ($f$) remains fixed across the *in vitro* samples over which the $r_1$-$r_2^*$ relaxivity is calculated:

$$f = \frac{Tf_1}{Tf_1 + Ft_1} = \frac{Tf_0}{Tf_0 + Ft_0}$$

(defining $[\Delta Tf] = Tf_1 - Tf_0$ and $[\Delta Ft] = Ft_1 - Ft_0$).

Under this condition:

S7) $$f = \frac{[\Delta Tf]}{[\Delta Ft]+[\Delta Tf]} = \frac{[Tf]}{[Ft]+[Tf]}$$

And therefore eq. S6 can be expressed as:

S8) $$\frac{\Delta R_1}{\Delta R_2^*} = \frac{\left(f*r_{(1,Tf)}+(1-f)r_{(1,Ft)}\right)[\Delta iron]+r_{(1,M)}[\Delta M]}{\left(f*r_{(2,Tf)}+(1-f)r_{(2,Ft)}\right)[\Delta iron]+r_{(2,M)}[\Delta M]}$$

Where $[\Delta Ft] + [\Delta Tf] = [\Delta iron]$.

In the ferritin-transferrin mixtures experiments, the liposomal fraction, which mimics the effect of myelin, was fixed at 17.5%. Therefore, in this case $[\Delta M] = 0$ and eq. S8 reduces to:

S9) $$\frac{\Delta R_1}{\Delta R_2^*} = \frac{\left(f*r_{(1,Tf)}+(1-f)r_{(1,Ft)}\right)[\Delta iron]}{\left(f*r_{(2,Tf)}+(1-f)r_{(2,Ft)}\right)[\Delta iron]} = \frac{\left(f*r_{(1,Tf)}+(1-f)r_{(1,Ft)}\right)}{\left(f*r_{(2,Tf)}+(1-f)r_{(2,Ft)}\right)}$$

Using the ferritin and transferrin relaxivities ($r_{(1/2,Tf/Ft)}$) measured for each liposomal iron compound individually (Figure 2a-c), we could test the prediction of this model. Notably, the theoretical $r_1$-$r_2^*$ relaxivities calculated with eq. S9 were in agreement with the experimental $r_1$-$r_2^*$ relaxivities (Sup. Figure 8), validating the presented biophysical framework *in vitro*. Moreover, the differences in the $r_1$-$r_2^*$ relaxivities measured for different transferrin-ferritin fractions were above the detection limit of this MRI measurement as estimated in a scan-rescan experiment (MAE=$3.9*10^{-4}$, Sup. Figure 9). Therefore, this toy example demonstrates that the heterogeneity in the iron environment can produce measurable changes in the $r_1$-$r_2^*$ relaxivity, which are in agreement with the biophysical modeling of this measurement.

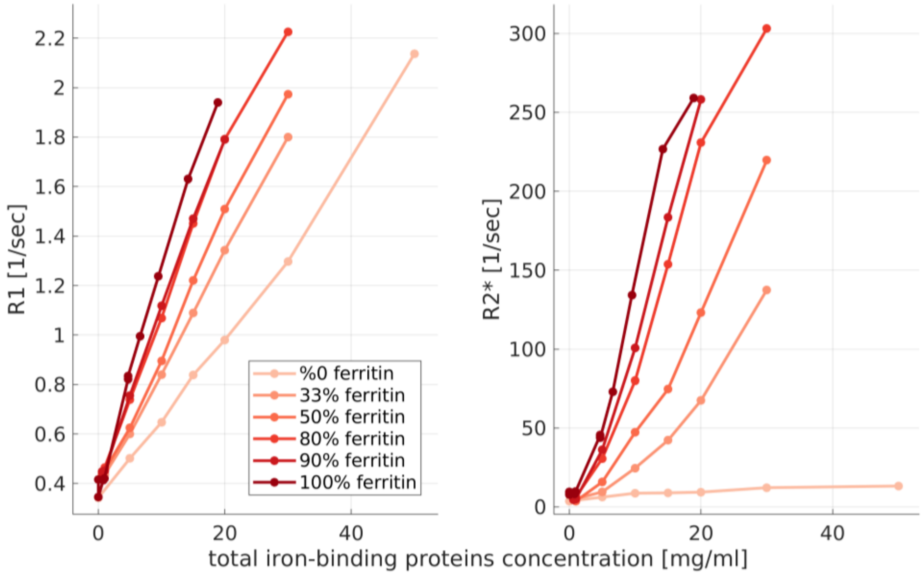


**Sup. Figure 6: The dependency of $R_1$ (left) and $R_2^*$ (right) on the total iron-binding proteins concentration for six transferrin-ferritin mixtures.** Each mixture has a different transferrin-ferritin fraction (different colors, legend shows the percentage of ferritin in the mixture). Data points are median values of different transferrin-ferritin samples, line connect between samples with the same transferrin-ferritin fraction and varying total iron-binding protein concentrations.

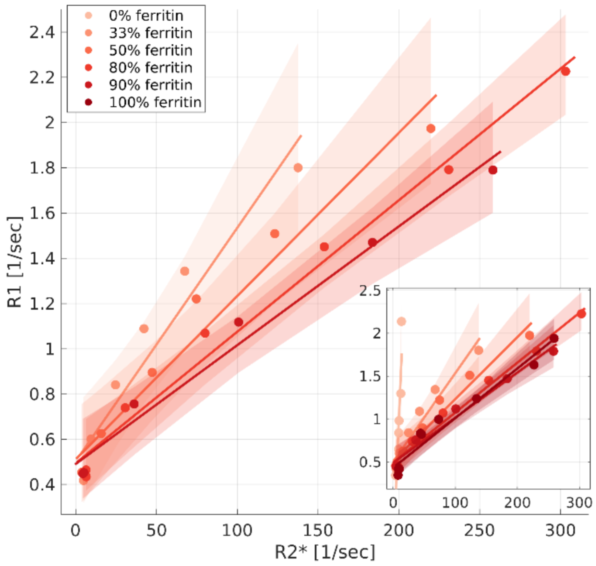


**Sup. Figure 7: The dependency of** $R_1$ **on** $R_2^*$ **for four transferrin-ferritin mixtures.** Each mixture has a different transferrin-ferritin fraction (different colors, legend shows the percentage of ferritin in the mixture). Data points represent median values of samples with varying total iron-binding proteins

concentrations. The linear relationships of $R_1$ and $R_2^*$ are marked by lines. The slopes of these lines are the $r_1$-$r_2^*$ relaxivities. Shaded areas represent the 95% confidence bounds of the linear fit. Inset shows the pure ferritin (100% ferritin) and pure transferrin (0% ferritin) samples as well.

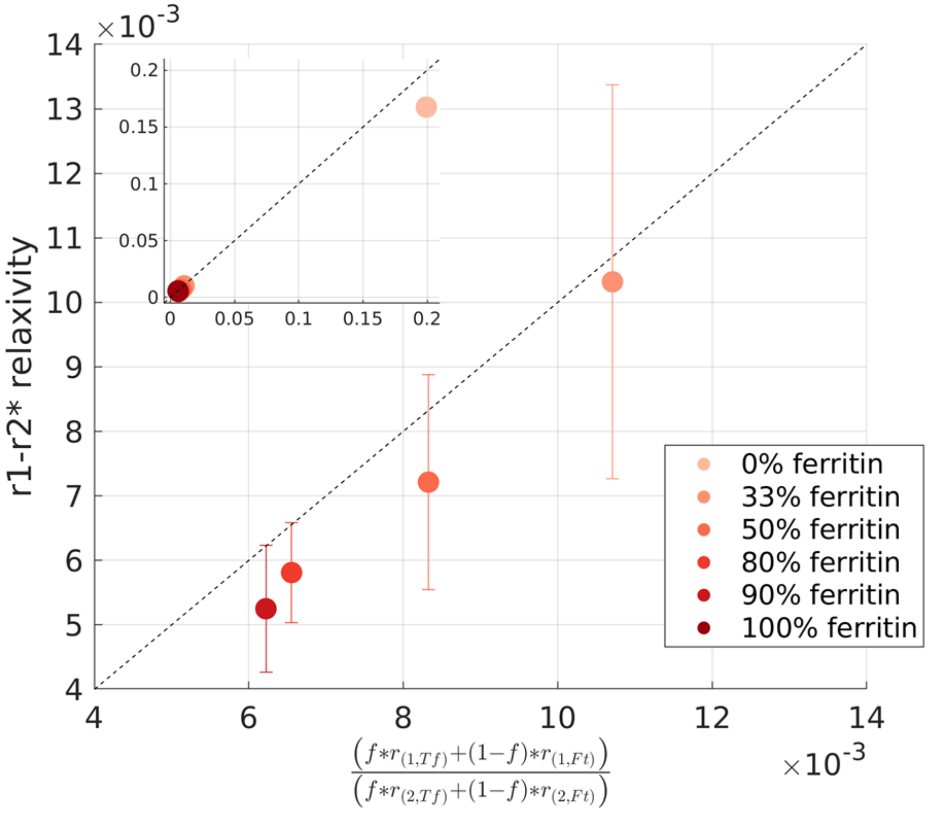


***Sup. Figure 8: The theoretical $r_1$-$r_2^*$ relaxivities calculated with eq. S9 are in agreement with the experimental $r_1$-$r_2^*$ relaxivities.*** *The y-axis shows the $r_1$-$r_2^*$ relaxivity calculated for four transferrin-ferritin mixtures with different transferrin-ferritin fractions (different colors, Sup. Figure 7). Data points represent the slope of the r1-r2* linear fit (calculated over N=7 independent samples for ferritin concentrations < 90%, N=6 for 90% ferritin and N=10 for 100% ferritin). Error bars show the 95% confidence bounds of the linear fit. The x-axis shows the prediction for the $r_1$-$r_2^*$ relaxivity based on eq. S9. The ferritin and transferrin relaxivities in the equation were plugged in based on our experimental results for liposomal ferritin and liposomal transferrin samples (Figure 2a-c). f represents the transferrin-ferritin fractions and varies between data points. Dashed line is the identity line. Inset shows the data points for pure ferritin (100% ferritin) and pure transferrin (0% ferritin) samples as well. Note that for these pure samples the prediction is already presented in figure 2e.*

## Supplementary Figure 9

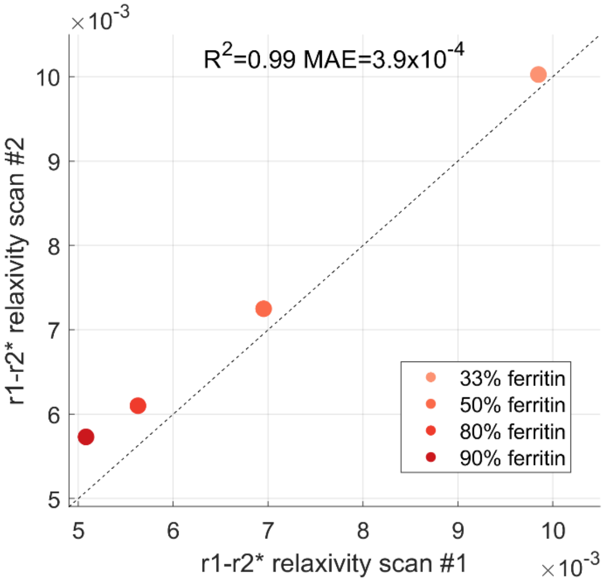


***Sup. Figure 9: The reproducibility of the $r_1$-$r_2^*$ relaxivity measurement in vitro.*** *The reproducibility of the $r_1$-$r_2^*$ relaxivity measurement for in vitro ferritin-transferrin mixtures was estimated based on scan-rescan experiments. Four different transferrin-ferritin mixtures were scanned twice (on different days). Each mixture experiment had a different transferrin-ferritin fraction (different colors, legend shows the percentage of ferritin in the mixture). The $r_1$-$r_2^*$ relaxivity of each mixture experiment was calculated over samples with the same transferrin-ferritin fraction but varying total iron-binding protein concentrations. Figure shows the $r_1$-$r_2^*$ relaxivity values measured in the first scan (x-axis) vs. the $r_1$-$r_2^*$ relaxivity values measured in the second scan (y-axis) for each in vitro experiment. Dashed line is the identity line. The measured scan-rescan mean absolute error (MAE) represents an experimental estimate of the detection limit of the $r_1$-$r_2^*$ relaxivity.*

## Supplementary Section 5 (Supplementary Figure 10)

## The effect of hemoglobin of the r1-r2* relaxivity.

We evaluated the effect of non-heme iron (ferritin, transferrin and ferrous iron) on the iron relaxivity. However, brain tissue also includes high concentration of hemoglobin in the blood[3]. In order to assess the effect of hemoglobin on the $r_1$-$r_2^*$ relaxivity, we performed additional in vitro experiments containing hemoglobin. First, we assessed the $r_1$-$r_2^*$ relaxivity of hemoglobin. Next, we examined whether the $r_1$-$r_2^*$ relaxivities of ferritin and transferrin change in the presence of hemoglobin (two different hemoglobin concentrations were tested). The hemoglobin concentrations in this experiment were constrained by its solubility (20mg/mL for hemoglobin alone, and lower when it is in a mixture with other iron-binding proteins). Sup. Figure 10a-b shows the dependency of $R_1$ and $R_2^*$ on the iron compound concentration.

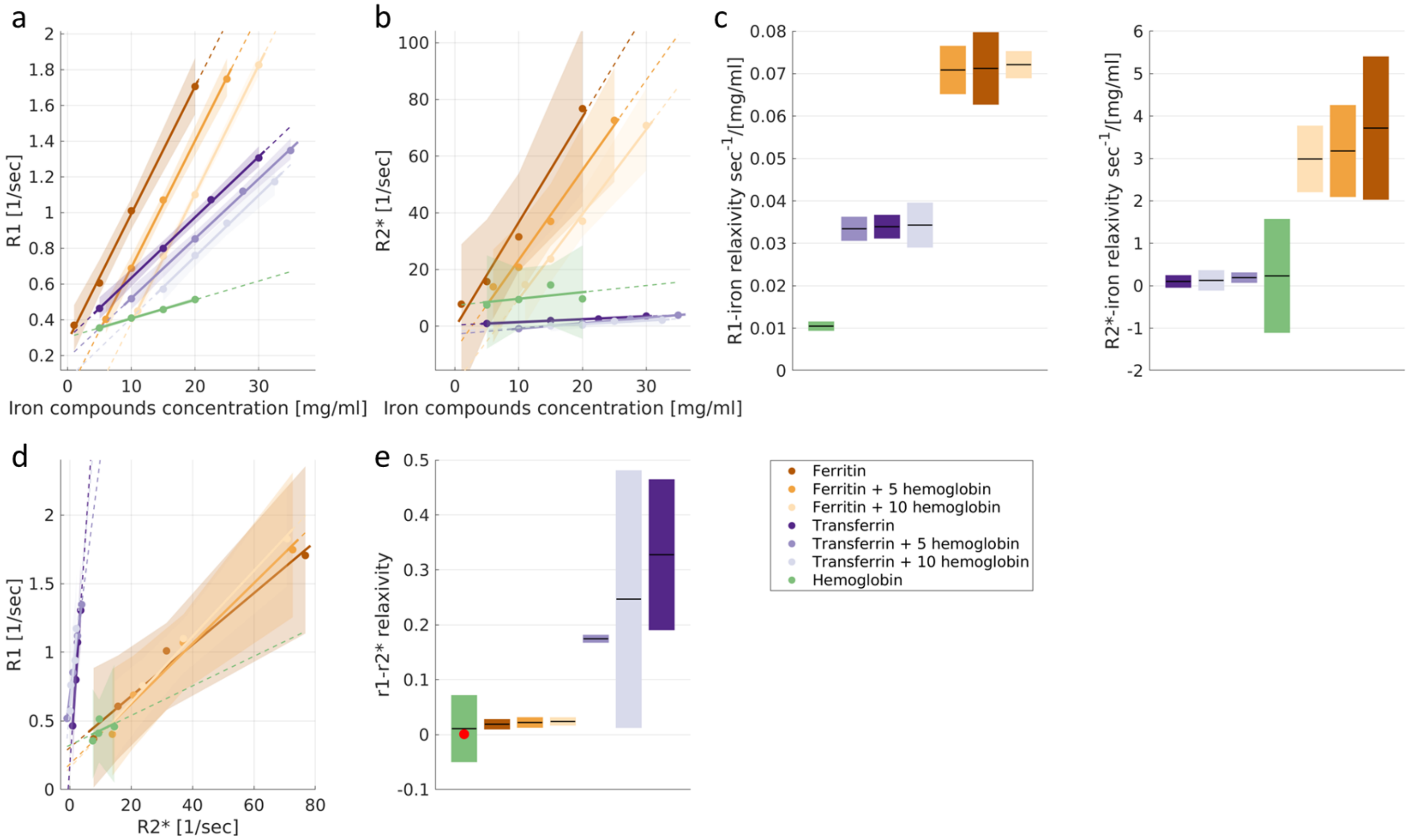


***Sup. Figure 10: the effect of hemoglobin on the r1-r2* relaxivity. (a-b****)* *The dependency of R1 and R2* on the iron compound concentrations for different iron environments: free ferritin, free transferrin, free hemoglobin, ferritin-hemoglobin and transferrin-hemoglobin mixtures (in two different hemoglobin concentration: 5 & 10 mg/ml). N=4 independent samples for each environment. For mixtures- data points represent samples with varying ferritin/transferrin concentrations while hemoglobin concentrations were kept constant (x-axis shows total iron compound concentrations including hemoglobin). The linear relationships between relaxation rates and iron compounds concentrations are marked by lines. We define the slopes of these lines as the iron relaxivities. Dashed lines represent extrapolation of the linear fit. Data points show median values of each sample, shaded areas represent the 95% confidence bounds.* ***(c)*** *The iron relaxivities of R1 and R2* for ferritin and transferrin in the presence of hemoglobin. Iron relaxivities are calculated by taking the slopes of the linear relationships shown in (a,b), and are measured in [sec-1/(mg/ml)]. For each box, the central mark is the iron relaxivity (slope); the box shows the 95% confidence bounds of the linear fit.* ***(d)*** *The dependency of R1 on R2* for ferritin and transferrin in the presence of hemoglobin. Data points represent samples with varying iron compound concentrations. The linear relationships of R1 and R2* are marked by lines. The slopes of these lines are the r1-r2* relaxivities. Data points show median values of each sample, dashed lines represent extrapolation of the linear fit. Shaded areas represent the 95% confidence bounds.* ***(e)*** *The r1-r2* relaxivities in the presence of hemoglobin. For each box, the central mark is the r1-r2* relaxivity, and the box shows the 95% confidence bounds of the linear fit. Red dot indicates the deoxyhemoglobin r1-r2* relaxivity estimated from the ratio between the R1 and R2* relaxivities of deoxyhemoglobin in blood reported by Blockley et al. [5]. Even in the presence of hemoglobin, ferritin and transferrin have distinct r1-r2* relaxivities ($p<0.005$ when comparing all ferritin-containing samples to all transferrin-containing samples, bonferroni-corrected one-way ANCOVA test).*

Hemoglobin has a distinct $R_1$ relaxivity ($p<0.0001$ when comparing hemoglobin to all other samples, bonferroni-corrected ANCOVA test), and an $R_2^*$ relaxivity similar to that of transferrin ($p<0.0001$ when comparing hemoglobin to ferritin and ferritin-hemoglobin mixtures, p=n.s for comparing to transferrin and transferrin-hemoglobin mixtures, bonferroni-corrected ANCOVA test). Importantly, while adding hemoglobin to ferritin and transferrin affects $R_1$ and $R_2^*$ values, the relaxivities of both ferritin and transferrin are not statistically affected by the presence of hemoglobin (Sup. Figure 10c, $p>0.05$ for comparing transferrin to transferrin-hemoglobin mixtures and ferritin to ferritin-hemoglobin mixtures, bonferroni-corrected ANCOVA test). Evaluating the $r_1$-$r_2^*$ relaxivities, we find that hemoglobin has lower values similar to ferritin (Sup. Figure 10d). Importantly, we compared this result with values previously published in the literature. Blockley et al. evaluated the dependency of whole blood $R_1$ and $R_2^*$ values on the deoxyhemoglobin concentrations[5]. We divided the $R_1$ and $R_2^*$ relaxivity of deoxyhemoglobin to get an estimation for the $r_1$-$r_2^*$ relaxivity (eq. 2). The prediction from the literature is presented in a red data point in Sup. Figure 10e. We find great agreement between our estimation of the hemoglobin $r_1$-$r_2^*$ relaxivity, and the literature reported values. Next, we tested whether the $r_1$-$r_2^*$ relaxivities of ferritin and transferrin are affected by the presence of hemoglobin (Sup. Figure 10e). We found that even in hemoglobin mixtures, ferritin and transferrin have distinct relaxivities ($p<0.005$ when comparing all ferritin-containing samples to all transferrin-containing samples, bonferroni-corrected ANCOVA test). Adding hemoglobin to ferritin and transferrin did not have significant effect on the $r_1$-$r_2^*$ relaxivity ($p>0.05$ bonferroni-corrected ANCOVA test). Notably, we scanned the hemoglobin samples twice, immediately after the preparation and a week later. While the rest of our *in vitro* experiments were stable and reproducible for different scan times, hemoglobin samples scanned a week after the preparation showed aggregation, visible both by eye and in the MRI scans. This aggregation was less visible in the first scan, immediately after the preparation. Therefore, we used results from the first scan for the analysis. However, aggregation processes may still impact these results.

Importantly, these *in vitro* experiments may not capture fully the effect of hemoglobin on the $r_1$-$r_2^*$ relaxivity in the *in vivo* brain. Particularly, the hemoglobin concentration in the blood is very high. We can simulate to what extent hemoglobin can affect the *in vivo* $r_1$-$r_2^*$ relaxivity. In normal gray-matter and white-matter, approximately 4–6% and 1–3% of the tissue volume is occupied by blood[6]. Assuming an extreme scenario where all blood volume is occupied by deoxyhemoglobin, and that the $r_1$-$r_2^*$ relaxivity of deoxyhemoglobin in blood is as reported in the literature[5], the $r_1$-$r_2^*$ relaxivity effect in a voxel with 6% blood volume would be:

$$hemoglobin\, r_1 - r_2^* \, relaxixity * blood\ volume = 7.2 * 10^{-4} * 0.06 = 4.3 * 10^{-5}$$

This is less than 1% percent of the average $r_1$-$r_2^*$ relaxivity measured in the *in vivo* brain. Therefore, based on this simulation, hemoglobin is not expected to be the main source governing the $r_1$-$r_2^*$ relaxivity contrast in the brain. Nonetheless, it could be that some of the $r_1$-$r_2^*$ relaxivity effects that we measured in the brain are related to hemoglobin. We therefore believe that the $r_1$-$r_2^*$ relaxivity in the brain is probably not sensitive exclusively to ferritin and transferrin. Hemoglobin and many other aspects of the iron homeostasis could be reflected in the $r_1$-$r_2^*$ relaxivity measurement.

## Supplementary Section 6 (Supplementary Figures 11-13)

## The dependency of the iron relaxivity on the liposomal fraction.

$R_1$ and $R_2^*$ measured in the brain are known to be sensitive to myelin content[4,7–12]. Myelin is composed mainly of lipids, though it also includes proteins. We tested the effect of the myelin fraction on iron relaxivity by varying the liposomal and protein (BSA) fractions in our phantoms.

In histological studies of brain iron, the iron concentrations often are reported relative to the wet weight, as this is considered more accurate[4]. To match our *in vitro* analysis to brain histology as much as possible, we calculated the iron-binding proteins' concentrations relative to the water fraction ([mg/wet ml]). This was done by computing the ratio between the iron concentration and the water fraction (which is complementary to the liposomal or protein fractions). The iron relaxivities shown in Figure 2 were therefore calculated as the linear dependencies of relaxation rates on the iron-binding protein concentration relative to the water fraction. Sup. Figure 11 presents the effect of the variable liposomal (or BSA) fractions on the iron relaxivity and on the $r_1$-$r_2^*$ relaxivity. When iron-binding protein concentrations were not calibrated to the water fraction (units of [mg/ml]), some variability in $R_1$ and $R_2^*$ values for different liposomal (or BSA) fractions was observed (Sup. Figure 11a,c). However, the iron relaxivities of different iron environments were still distinct, despite the liposomal (or BSA) fractions' variability. Calibrating the iron-binding protein concentrations to the water fraction (units of [mg/wet ml]) further eliminated the effect of the variable liposomal (or BSA) fractions on the iron relaxivities (Sup. Figure 11b,d). This is evident by the alignment of the data points with different liposomal (or BSA) fractions along the iron relaxivity's linear fits. Therefore, while the non-water fraction has an effect on the relaxation rates, it does not disrupt the sensitivity of the iron relaxivities to the molecular type of iron.

We further estimated the effect of the liposomal (or BSA) fractions on the $r_1$-$r_2^*$ relaxivity. In Sup. Figure 11e we show the same $r_1$-$r_2^*$ relaxivities presented in Figure 2**,** but now the liposomal (or BSA) fractions are indicated by different symbols. Similarly to the iron relaxivities, the $r_1$-$r_2^*$ relaxivities of different iron environments were distinct, even though they were calculated across varying liposomal (or BSA) fractions. Moreover, we estimated the $r_1$-$r_2^*$ relaxivity separately for each liposomal (or BSA) fraction (Sup. Figure 11f). We find that the $r_1$-$r_2^*$ relaxivity differences between iron compounds are greater than the differences within each iron compound for the variable liposomal (or BSA) fractions.

The dependency of $R_1$ on the macromolecular tissue volume (MTV) was associated with lipid composition in our previous work[13]. We tested this finding in the presence of iron by calculating the $R_1$-MTV dependencies for different types of lipids mixed with iron (Sup. Figure 12a-b). Notably, in the current study we sampled only three liposomal fractions, and therefore the variation in the iron concentration between the samples was much richer than the variation in lipid concentration. Still, we were able to replicate our finding regarding the sensitivity of the $R_1$-MTV dependency to lipid type. We find that the $R_1$-MTV dependencies are different for two types of lipid mixtures (phosphatidylcholine (PC) and phosphatidylcholine-sphingomyelin (PC-SM)) mixed with ferrous ($Fe^{2+}$) iron (Sup. Figure 12a). In the presence of ferritin, the difference between the $R_1$-MTV dependencies of the two lipids is smaller (Sup. Figure 12b).

Unlike the $R_1$-MTV dependency, the $r_1$-$r_2^*$ relaxivity is insensitive to the lipid composition (Sup. Figure 12c): different lipids mixed with ferritin have a similar $r_1$-$r_2^*$ relaxivity (p(one-sided ANCOVA)=0.11). The variability in the $r_1$-$r_2^*$ relaxivity was much bigger when comparing these different liposomal ferritin samples to liposomal transferrin (p(one-sided ANOCVA)$<10^{-7}$). Compared to the $R_1$-MTV dependencies, we find that the $r_1$-$r_2^*$ relaxivity provides a better distinction between iron compounds. Sup. Figure 13 presents the $r_1$-$r_2^*$ relaxivities and the $R_1$-MTV dependencies for different iron compounds. One-sided ANCOVA tests for the $R_1$-MTV dependencies reveal that the only significant distinction is between the BSA-ferritin mixture and all the liposomal iron compounds (p(ANCOVA)$<10^{-5}$). The rest of the iron environments are indistinguishable in terms of their $R_1$-MTV dependencies. On the contrary, all iron environments were distinguishable in terms of their $r_1$-$r_2^*$ relaxivity (p(ANCOA)$<10^{-32}$).

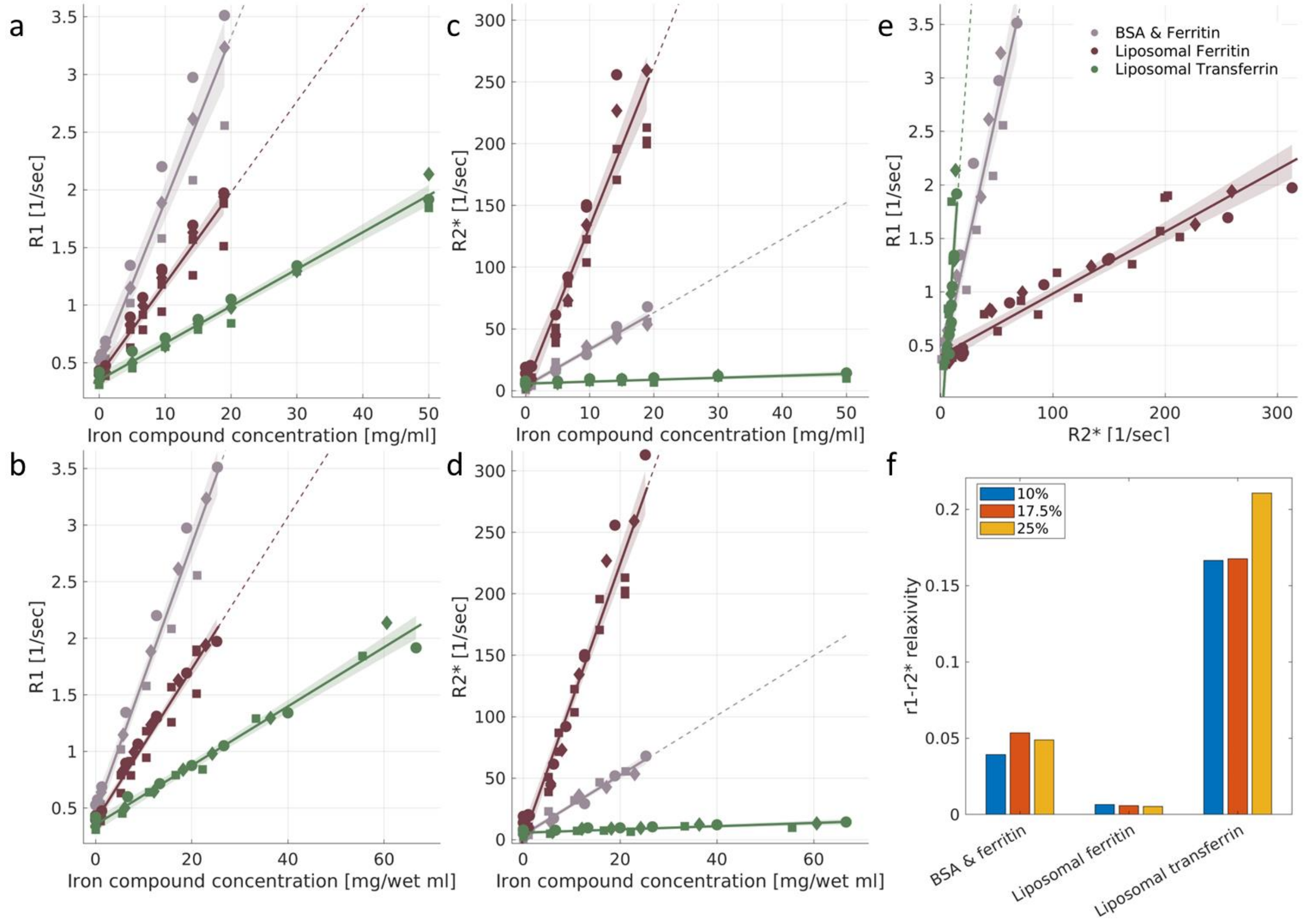


***Sup. Figure 11: Relaxivities are stable across liposomal or BSA fractions. (a)*** *The dependency of $R_1$ on the iron-binding protein concentration for different liposomal (or BSA) fractions (different symbols) and different iron environments (different colors). Data points represent median values of biologically independent liposomal samples with varying liposomal and iron concentrations. The x-axis represents the absolute concentration of iron-binding proteins (not relative to the water concentration, as in b). The linear relationships between relaxation rates and iron-binding protein concentration are marked solid by lines. The slopes of these lines are defined as the iron relaxivities. $R_1$ values are affected by the variable liposomal (or BSA) fractions, but the iron relaxivities of different iron environments are still distinct, regardless of this manipulation. Dashed lines represent extrapolations of the linear fits. Shaded areas represent the 95% confidence bounds.* ***(b)*** *The dependency of $R_1$ on the iron-binding protein concentration for different liposomal (or BSA) fractions (different symbols) and different iron environments (different colors). Data is as in (a), but here the x-axis represents the concentration of iron-binding proteins relative to the water fraction (which varies with the liposomal or BSA fraction). This estimation, in units of [mg/wet ml], further eliminates the effect of the liposomal (or BSA) fraction on the iron relaxivities. This is evident by the alignment of the data points with different liposomal (or BSA) fractions (different symbols) along the iron relaxivity linear fit. Solid lines are the relaxivities, shaded areas represent the 95% confidence bounds.* ***(c-d)*** *A similar analysis for the $R_2^*$-iron relaxivity. The effect of the different liposomal (or BSA) fractions on the $R_2^*$-iron relaxivity is eliminated by the calculation of*

*the iron-binding proteins concentration relative to the water fraction ([mg/wet ml]). Solid lines are the relaxivities, shaded areas represent the 95% confidence bounds.* ***(e)*** *The dependency of $R_1$ on $R_2^*$ for different liposomal (or BSA) fractions (different symbols) and different iron environments (different colors). The $r_1$-$r_2^*$ relaxivities of different iron environments are distinct even when calculated across liposomal (or BSA) fractions. Solid lines are the relaxivities, shaded areas represent the 95% confidence bounds.* ***(f)*** *The $r_1$-$r_2^*$ relaxivity (y-axis) for different compounds of iron (liposomal ferritin, liposomal transferrin and BSA-ferritin mixture) in three different liposomal (or BSA) fractions (colors). The differences in the $r_1$-$r_2^*$ relaxivity between iron environments are greater than the differences within each iron environment for the variable liposomal (or BSA) fractions.*

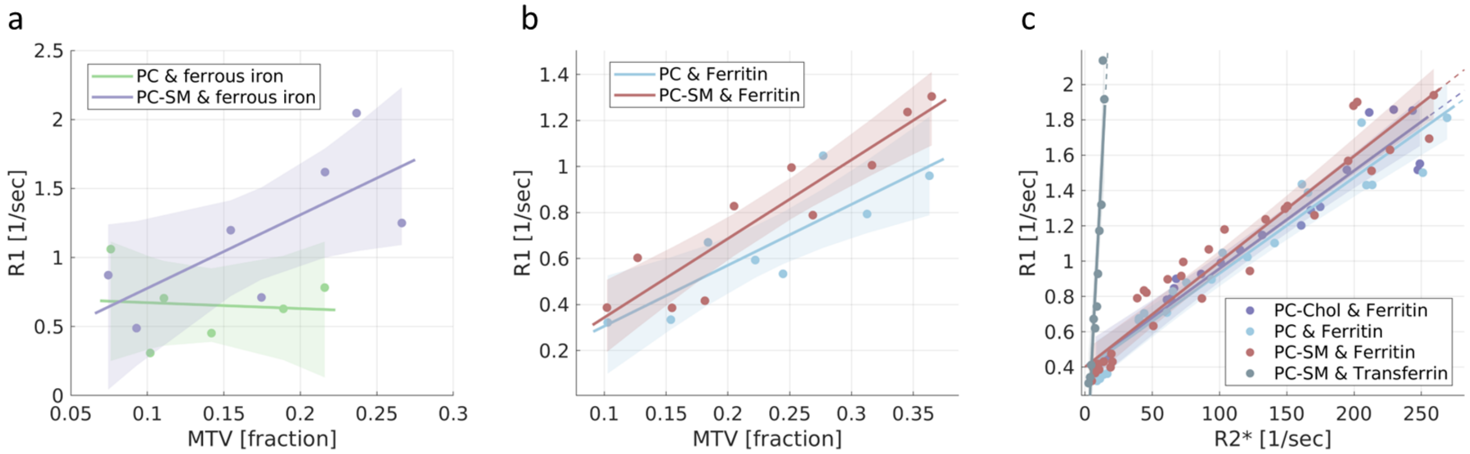


***Sup. Figure 12: The $r_1$-$r_2^*$ relaxivity is stable for different types of lipids, while the $R_1$-MTV dependency is sensitive to the lipid type. (a)*** *The dependency of $R_1$ on MTV for an iron ion compound ($Fe^{2+}$) mixed with two different lipids: phosphatidylcholine (PC, green) and a mixture of PC-sphingomyelin (PC-SM, blue). This result replicates the sensitivity of the MTV dependencies to lipid types[13] in $Fe^{2+}$-containing phantoms.* ***(b)*** *The dependency of $R_1$ on MTV for a second iron compound (ferritin) mixed with the same two lipids (PC and PC-SM).* ***(c)*** *The dependency of $R_1$ on $R_2^*$ ($r_1$-$r_2^*$ relaxivity) for four different iron-lipid mixtures: ferritin-PC ferritin-PC-SM, transferrin-PC-SM and ferritin-PC-cholesterol (PC-Chol, blue). The $r_1$-$r_2^*$ relaxivity is similar for the different lipid types mixed with ferritin, and the main difference is between the iron binding proteins; i.e., transferrin sample and the ferritin samples. For all panels the linear relationships are marked by solid lines. Shaded areas represent the 95% confidence bounds.*

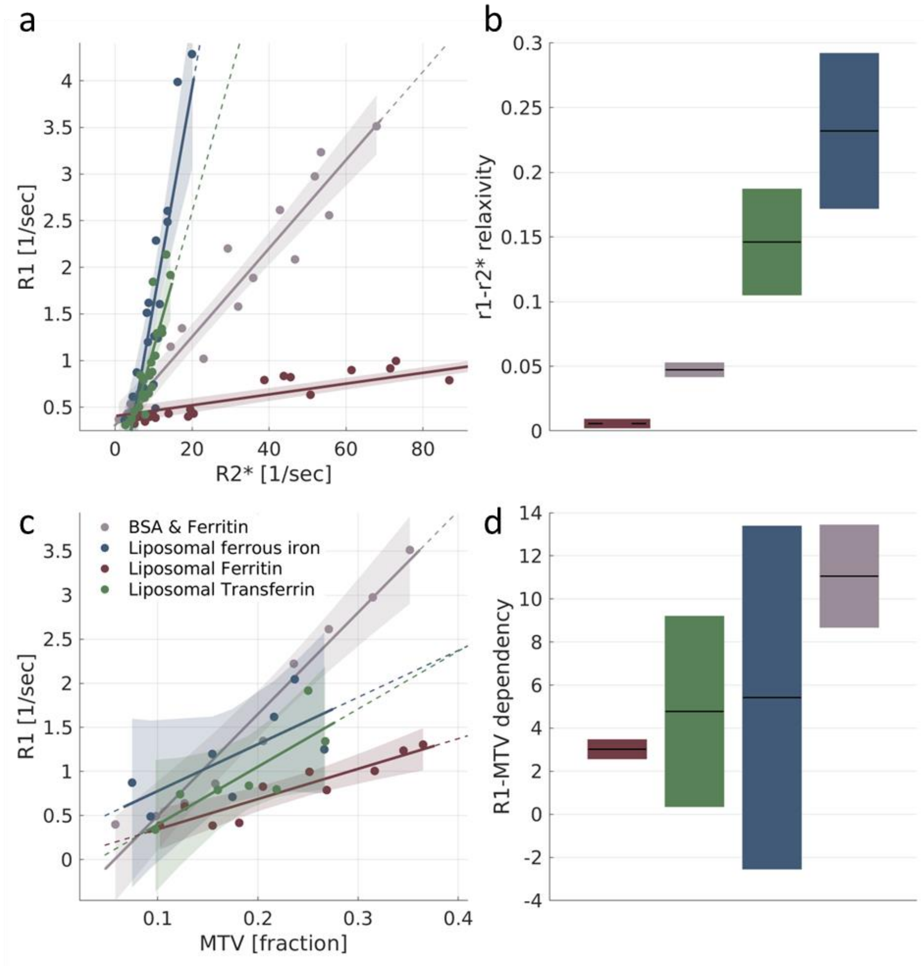


***Sup. Figure 13: Iron environments are less distinguishable with MTV dependencies than with the $r_1$-$r_2^*$ relaxivity.*** ***(a)*** *The dependency of $R_1$ on $R_2^*$ ($r_1$-$r_2^*$ relaxivity) for different iron environments: liposomal-ferritin (N=36), BSA-ferritin mixture (N=22), liposomal transferrin (N=22) and liposomal Ferrous iron (N=20). Liposomal samples are based on PC-sphingomyelin. Data points represent median values of biologically independent samples with varying iron compounds concentrations relative to the water fraction. The linear relationships between relaxation rates are marked by lines, whose slopes represent the $r_1$-$r_2^*$ relaxivities. Dashed lines represent extrapolations of the linear fits. Shaded areas represent the 95% confidence bounds. The x-axis presents only partial range of $R_2^*$ values, similar to Figure 2d (for the entire $R_2^*$ range, see the inset of Figure 2d).* ***(b)*** *The $r_1$-$r_2^*$ relaxivities are different for these four iron environments. For each box, the central line marks the $r_1$-$r_2^*$ relaxivity, and the box shows the 95% confidence bounds of the linear fit.* ***(c)*** *The dependency of $R_1$ on MTV for these four iron environments. Data points represent median values of biologically independent samples with varying iron compounds concentrations relative to the water fraction. The linear relationships between $R_1$ and MTV are marked by lines, whose slopes represent the $R_1$-MTV dependencies. Dashed lines represent extrapolations of the linear fits. Shaded areas represent the 95% confidence bounds.* ***(d)*** *The $R_1$-MTV dependencies for the four iron environments. For each box, the central line marks the $R_1$-MTV dependency, and the box shows the 95% confidence bounds of the linear fit.*

## Supplementary Figure 14

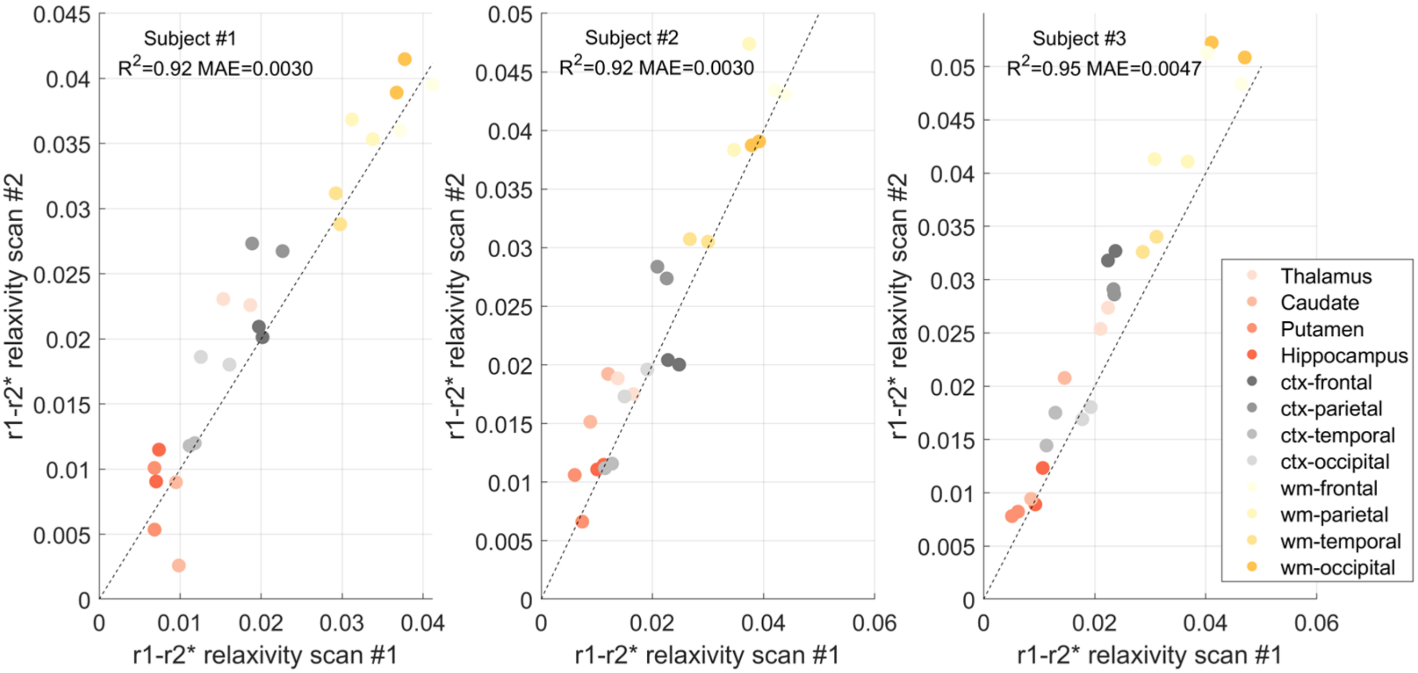


***Sup. Figure 14: The reproducibility of the $r_1$-$r_2^*$ relaxivity measurement in the in vivo brain.*** *The reproducibility of the $r_1$-$r_2^*$ relaxivity measurement in the in vivo brain was estimated based on scan-rescan experiments in three human subjects. Each subject was scanned twice in the MRI (on different days). The $r_1$-$r_2^*$ relaxivity was calculated for each scan in 12 different brain regions (different colors) in both hemispheres. Panels show the $r_1$-$r_2^*$ relaxivity values measured in the first scan (x-axis) vs. the $r_1$-$r_2^*$ relaxivity values measured in the scanned scan for each subject. Dashed line is the identity line. The measured scan-rescan mean absolute error (MAE) represents an experimental estimate of the detection limit of the $r_1$-$r_2^*$ relaxivity.*

# Supplementary Section 7 (Supplementary Figures 15-16)

# The intercept of the R1-R2* linear fit.

The intercept of the $R_1$-$R_2^*$ linear fit represents the residual $R_1$ not explained by $R_2^*$. Therefore, this measurement has the potential to be sensitive to biological sources affecting exclusively $R_1$ and not $R_2^*$. Based on the relaxivity model ("*In vivo* iron relaxivity model" in methods), the expression for intercept can be deduced:

$$\text{S10)} \qquad R_1 = r_{(1,a)}[a] + c_{(1,a)}$$

$$S11) \qquad R_2^* = r_{(2,a)}[a] + c_{(2,a)}$$

Substituting Eq. S10 in Eq. S11:

$$S12) \qquad R_1 = \frac{r_{(1,a)}}{r_{(2,a)}} R_2^* + c_a$$

Where the intercept can be expressed as:

$$S13) \qquad c_{a/b} = c_{(1,a/b)} - \frac{r_{(1,a/b)}}{r_{(2,a/b)}} c_{(2,a/b)}$$

Therefore, it should be sensitive both to the non-iron contributions to $R_1$ and $R_2^*$ ($c_{(1/2,a/b)}$), and to their iron relaxivities ($\frac{r_{(1,a/b)}}{r_{(2,a/b)}}$). We first tested the biophysical interpretation of the intercept *in vitro*. Sup. Figure 15 shows the intercept of different iron forms. Interestingly, the intercept seems to be less sensitive to the molecular environment of the iron forms compared to the $r_1$-$r_2^*$ relaxivity. For example, the $r_1$-$r_2^*$ relaxivity varied greatly between free and liposomal ferritin (Figure 2e, p<10-5, Bonferroni corrected ANCOVA test). On the other hand, the intercept is not significantly different between free and liposomal ferritin (p=0.99, Bonferroni corrected ANCOVA test). The intercept is sensitive to the molecular form of iron, and is different for liposomal ferritin and liposomal transferrin (p=0.0036, Bonferroni corrected ANCOVA test). Nevertheless, the difference between liposomal ferritin and liposomal transferrin is more significant when measuring the $r_1$-$r_2^*$ relaxivity ($p<10^{-8}$, Bonferroni corrected ANCOVA test).

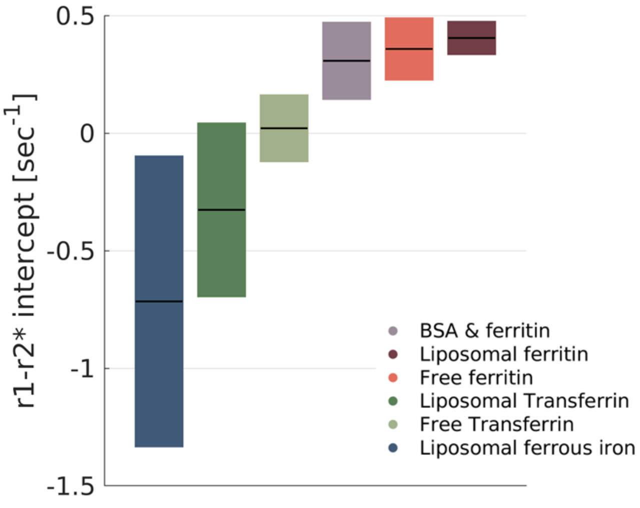


**Sup. Figure 15: The intercept of the R1-R2* linear fit in-vitro.** The intercept of the R1-R2* linear fit for different iron environments; free ferritin (N=20), liposomal ferritin (N=36), bovine serum albumin (BSA)-ferritin mixture (N=22), free transferrin (N=6), liposomal transferrin (N=22) and liposomal ferrous iron (N=20). For each box, the central mark is the intercept, and the box shows the 95% confidence bounds of the linear fit.

Next, we tested the intercept of the $R_1$-$R_2^*$ linear fit *in vivo*. Sup. Figure 16 shows the $R_1$-$R_2^*$ linear fit for four brain regions of a single subject. Sup. Figure 16b shows the variability of the intercept across young subjects for different brain regions. Interestingly, while white-matter regions tend to have high $R_1$, $R_2^*$ and $r_1$-$r_2^*$ relaxivity values, they have low intercept. Therefore, in the white-matter, most of the variability in $R_1$ is explained by $R_2^*$, implying for shared biological sources that govern both relaxation mechanisms in this tissue. On the contrary, gray-matter and subcortical regions have higher intercept, suggesting that in these regions there is a residual $R_1$ relaxation not explained by $R_2^*$. This residual $R_1$ could be attributed to biological sources affecting $R_1$ exclusively.

$R_1$ relaxation mechanisms are affected by local molecular interactions, while $R_2^*$ is sensitive to more global effects of extended paramagnetic interactions at the mesoscopic scale[14]. Therefore, it could be that the intercept is more sensitive to local molecular interactions that do not involve extended paramagnetic effects. Further work may provide insights into biological substrates that are characterized by such relaxation. To conclude, while we exploit the slope of the $R_1$-$R_2^*$ fit ($r_1$-$r_2^*$ relaxivity) for information on the iron homeostasis, the intercept might capture important information as well. Analyzing both the relaxivity and the intercept may contribute complementary information and allow better *in vivo* characterization of brain tissue.

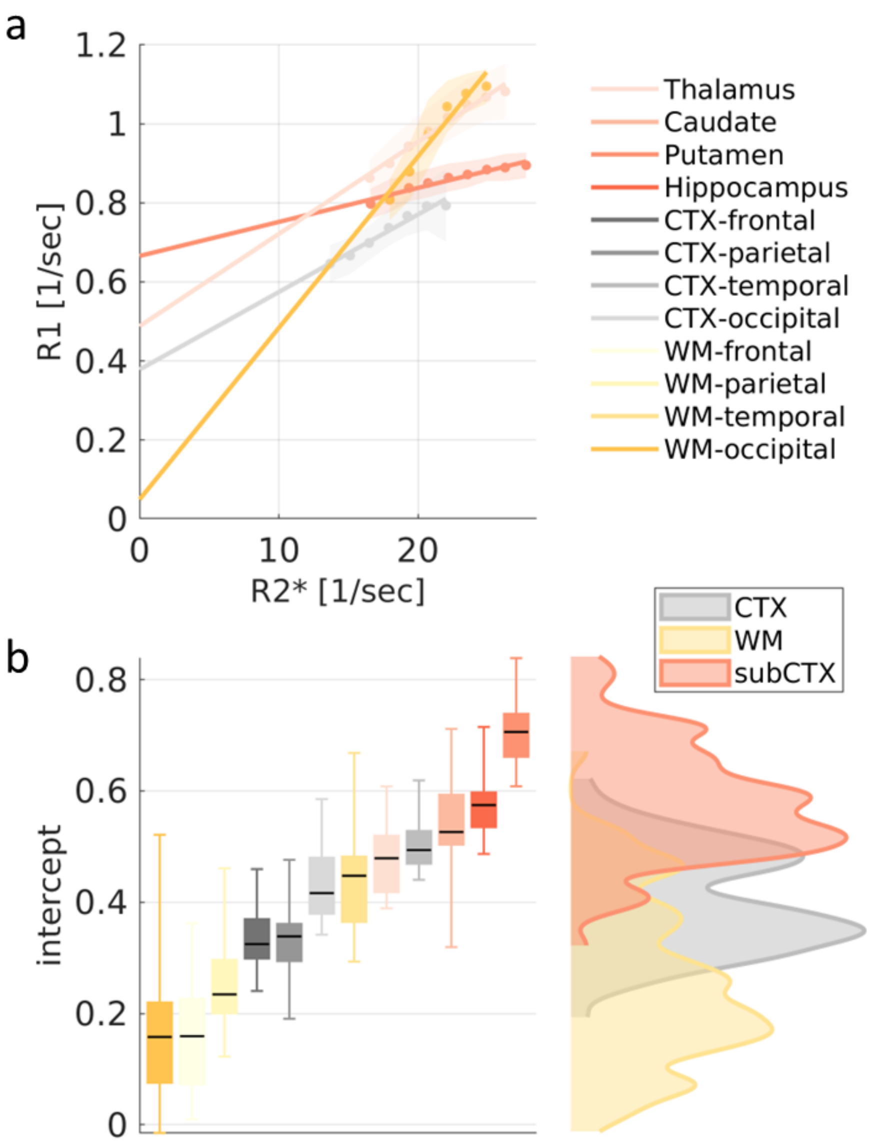


***Sup. Figure 16: The intercept of the R1-R2* linear fit in-vivo. (a)*** *The dependency of R1 on R2* in four representative brain regions (WM-occipital, CTX-occipital, Thalamus & Putamen) of a single subject. R2* and R1 were binned (dots represent the median; shaded areas represent the mean absolute deviation), and a linear fit was calculated. The slopes and intercepts of the linear fit vary across brain regions.* ***(b)*** *The intercept of the R1-R2* fit across the brain. Left: the reliability of the method in different brain regions as observed by the variation in the intercept across normal subjects (age 27±2, N = 21). The 25th, 50th and 75th percentiles and extreme data points are shown for each box. Right: the contrast of the intercept across the brain. Red, yellow and gray distributions represent the values of the intercept in sub-cortical (sub-CTX), white-matter (WM) and cortical (CTX) brain regions, respectively.*

# Supplementary Section 8 (Supplementary Figures 17-19)

# Voxel-wise r1-r2* relaxivity visualization.

Figure 3 and Figure 5 compare the contrast of $R_1$ and $R_2^*$ in the brain to the contrast generated by the $r_1$-$r_2^*$ relaxivity. The measurement of the $r_1$-$r_2^*$ relaxivity is calculated across all the voxels of a specific ROI in the brain (see "$r_1$-$r_2^*$ relaxivity computation for ROIs in the human brain" in Methods). Therefore, the contrasts are presented across different entire brain regions. In order to demonstrate a visualization of a voxel-wise $r_1$-$r_2^*$ relaxivity contrast, we generated representative maps of the local $r_1$-$r_2^*$ relaxivity in a healthy young subject and in a Meningioma patient. For this purpose, we used a moving-window approach, in which the $r_1$-$r_2^*$ relaxivity of each voxel is based on the local linear dependency of $R_1$ on $R_2^*$ in that voxel and all its neighboring voxels (125 voxels total, for more details see "Generating voxel-wise $r_1$-$r_2^*$ relaxivity visualizations" in Methods).

A comparison of the voxel-wise $r_1$-$r_2^*$ relaxivity to the $R_1$ and $R_2^*$ maps in the healthy brain can be seen in Sup. Figure 17. Similarly to the ROI-based approach (Figure 3), this voxel-wise comparison also shows that the $r_1$-$r_2^*$ relaxivity generates a unique contrast in the brain compared to $R_1$ and $R_2^*$. Interestingly, this local relaxivity contrast highlights the differences between superficial and deep white-matter. Such contrast was previously suggested to be driven by the microscopic iron distribution[15].

In meningioma patients, we show that the ROI-based approach for the $r_1$-$r_2^*$ relaxivity allows to enhance the contrast between tumor tissue and non-pathological tissue without contrast agent injection (Figure 5). A comparison of the voxel-wise $r_1$-$r_2^*$ relaxivity to the $R_1$ and $R_2^*$ maps and to the Gd-enhanced contrast in a representative meningioma patient can be seen in Sup. Figure 18. In this example, the boundaries of the tumor can be separated from the surrounding non-pathological tissue based on the voxel-wise contrast of the $r_1$-$r_2^*$ relaxivity. Importantly, for this patient we were able to replicate our ROI-based results on the voxel-wise level (Sup. Figure 19). Across voxels, we find that the $r_1$-$r_2^*$ relaxivity allows to distinguish between tumor tissue and non-pathological tissue better than $R_1$ and $R_2^*$ (for example, effect size for the difference between tumor and gray-matter is more than 10 times larger in the $r_1$-$r_2^*$ relaxivity compared to $R_1$ and $R_2^*$).

Nonetheless, these representative visualizations merely provide preliminary evidence for the adaptiveness of the $r_1$-$r_2^*$ relaxivity approach for voxel-wise analyses. Notably, the presented implementation still has some limitations. First, the moving-window approach used for calculating the local $r_1$-$r_2^*$ relaxivity leads to inherent smoothing. As a result, this approach is sensitive to partial volume

effects for voxels on the border between tissue types, which could be driving the observed contrast between superficial and deep white-matter and between tumor tissue and non-pathological tissue. In addition, the local computation of the $r_1$-$r_2^*$ relaxivity uses fewer voxels compared to the ROI-based approach. It also does not include the binning procedure prior to the fitting which we used in the ROI-based approach. Therefore, this computation is less stable and is more sensitive to the inherent SNR of $R_1$ and $R_2^*$, which is affected by magnetic field inhomogeneities, imperfections of the shim, and the heterogeneous magnetic susceptibility of the head. As a result, some of the calculated values in the brain are negative (4% of the voxels in the healthy subject and 11% of the voxels in the meningioma patient). These limitations should be accounted for prior to any future implementation of this voxel-wise approach for purposes other than visualization.

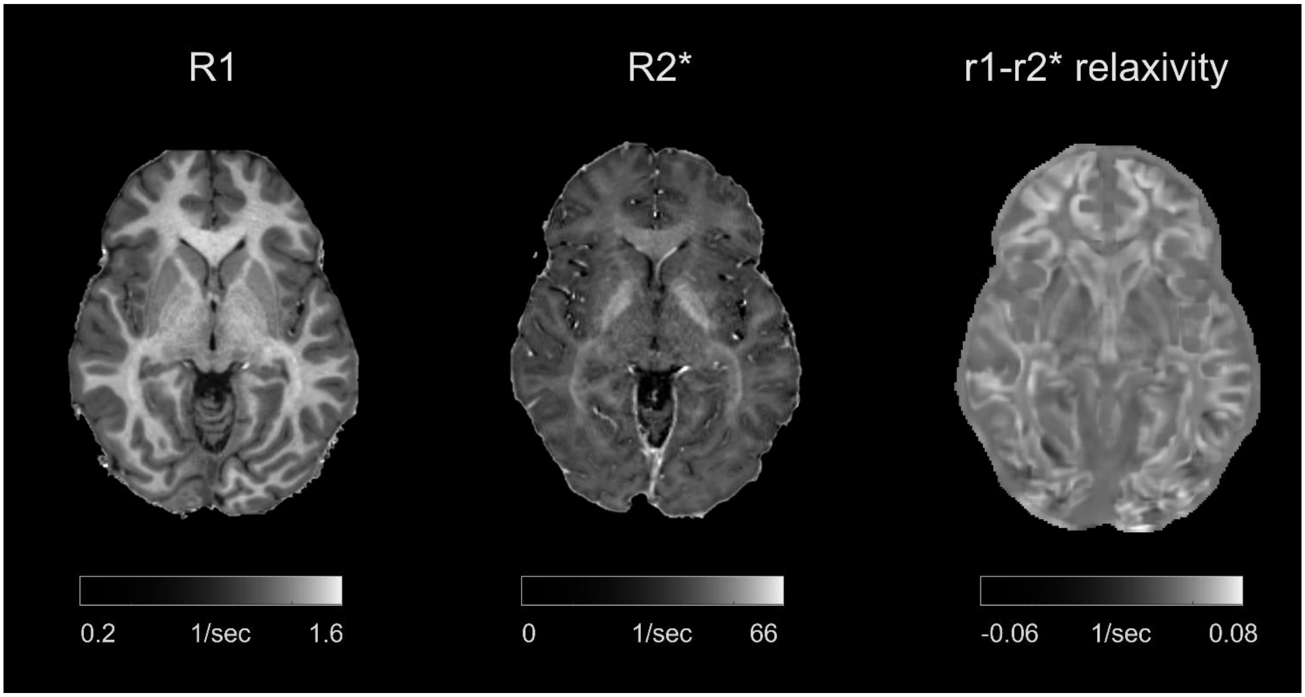


***Sup. Figure 17: Voxel-wise comparison of the $r_1$-$r_2^*$ relaxivity map to $R_1$ and $R_2^*$ maps in the in vivo healthy brain.*** *The maps of $R_1$ (left) and $R_2^*$ (middle) are compared to the local $r_1$-$r_2^*$ relaxivity visualization (right) on a representative young healthy subject. The voxel-wise visualization of the $r_1$-$r_2^*$ relaxivity in the brain was generated based on the local linear dependency of $R_1$ on $R_2^*$ using a moving-window approach (for more details see "Generating voxel-wise $r_1$-$r_2^*$ relaxivity visualizations" in Methods).*

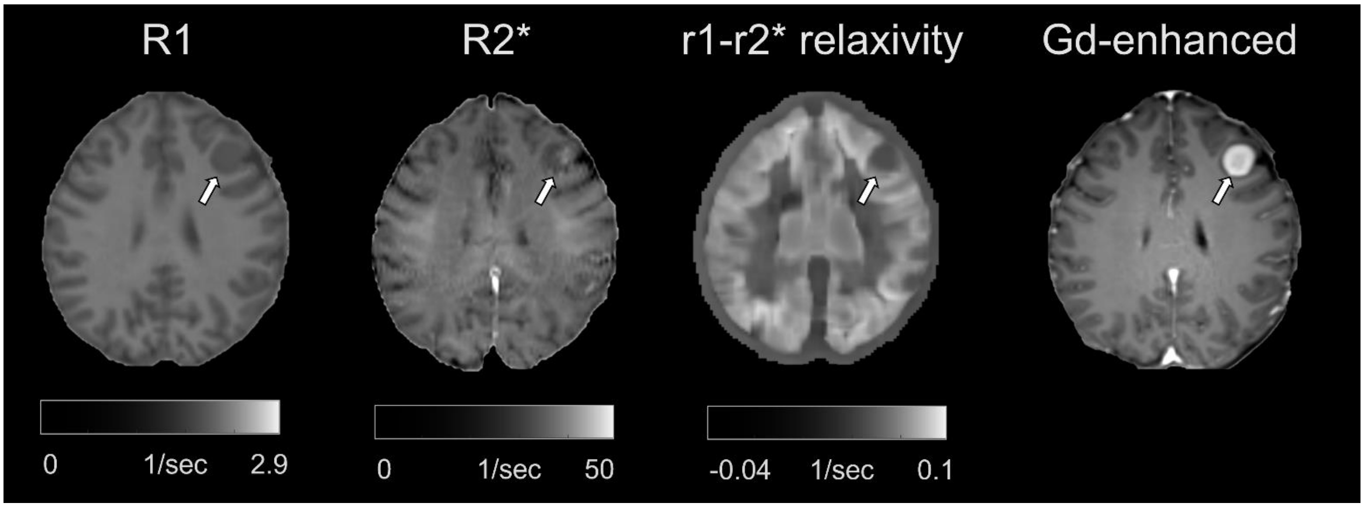


***Sup. Figure 18: Voxel-wise comparison of the $r_1$-$r_2^*$ relaxivity map to $R_1$ and $R_2^*$ maps and to the Gd-enhanced contrast in the in vivo brain of a meningioma patient.*** *Representative visualization of $R_1$, $R_2^*$, the voxel-wise $r_1$-$r_2^*$ relaxivity map and the Gd-enhanced contrast for a meningioma patient. Tumors are marked with arrows. The voxel-wise $r_1$-$r_2^*$ relaxivity map was generated based on the local linear dependency of $R_1$ on $R_2^*$ using a moving-window approach (for more details see "Generating voxel-wise $r_1$-$r_2^*$ relaxivity visualizations" in Methods).*

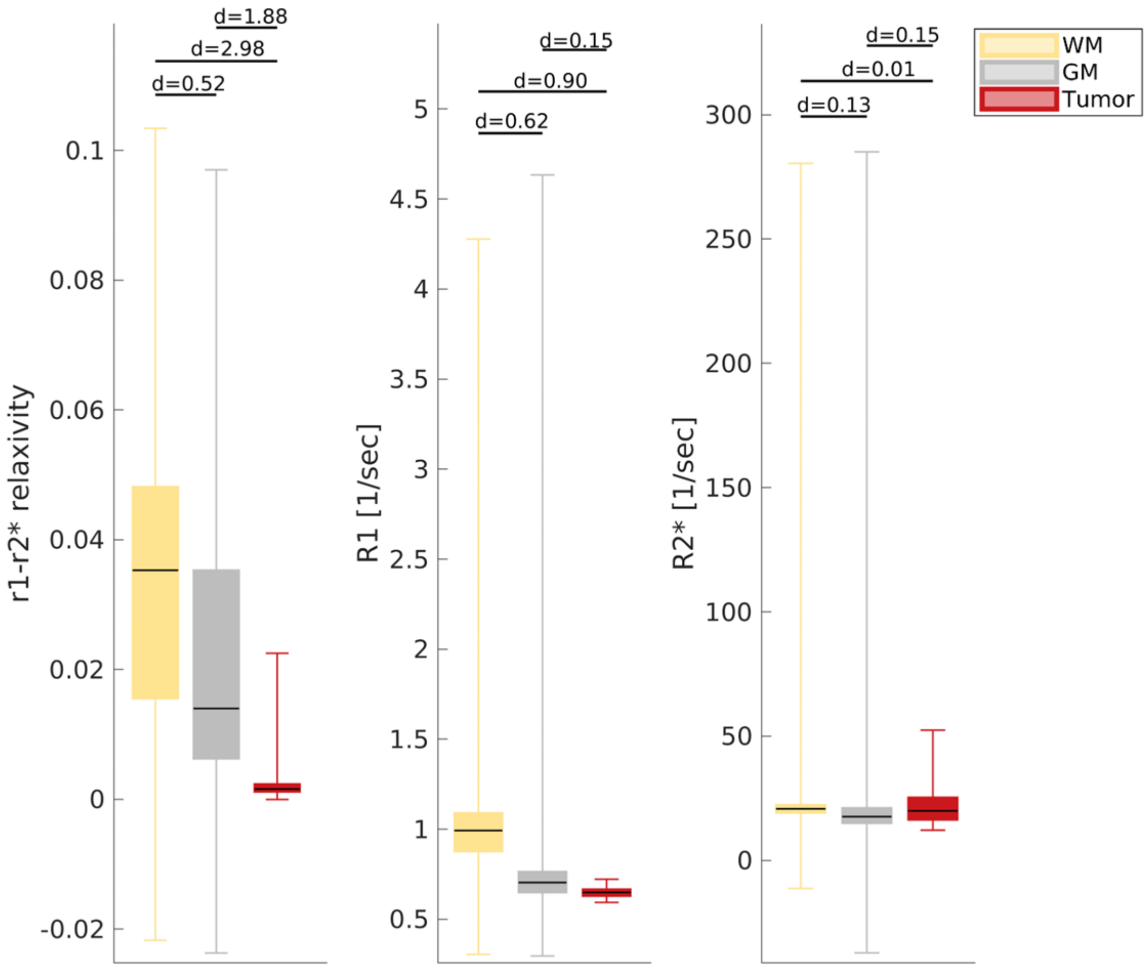


***Sup. Figure 19: The voxel-wise $r_1$-$r_2^*$ relaxivity enhances the contrast between tumor tissue and non-pathological tissue on a representative meningioma patient.*** *Replication of the ROI-based results presented in figure 4d-f on the voxel-wise level. The contrast between the white-matter (WM, N=60647 independent voxels), gray matter (GM, N=44251 independent voxels) and tumor tissues (N=313 independent voxels) is presented for $R_1$, $R_2^*$ and the voxel-wise $r_1$-$r_2^*$ relaxivity. The variation in each box is calculated across voxels in a representative meningioma patient (Sup. Figure 11). The 25th, 50th and 75th percentiles and extreme data points are shown for each box, whiskers show maximal and minimal values. The d-values represent the effect size (Cohen's d) of the differences between tissue types. Across voxels, the $r_1$-$r_2^*$ relaxivity allows to distinguish between tumor tissue and non-pathological tissue better than $R_1$ and $R_2^*$. Estimates in non-pathological tissues are for the tumor-free hemisphere.*

# Supplementary Figure 20

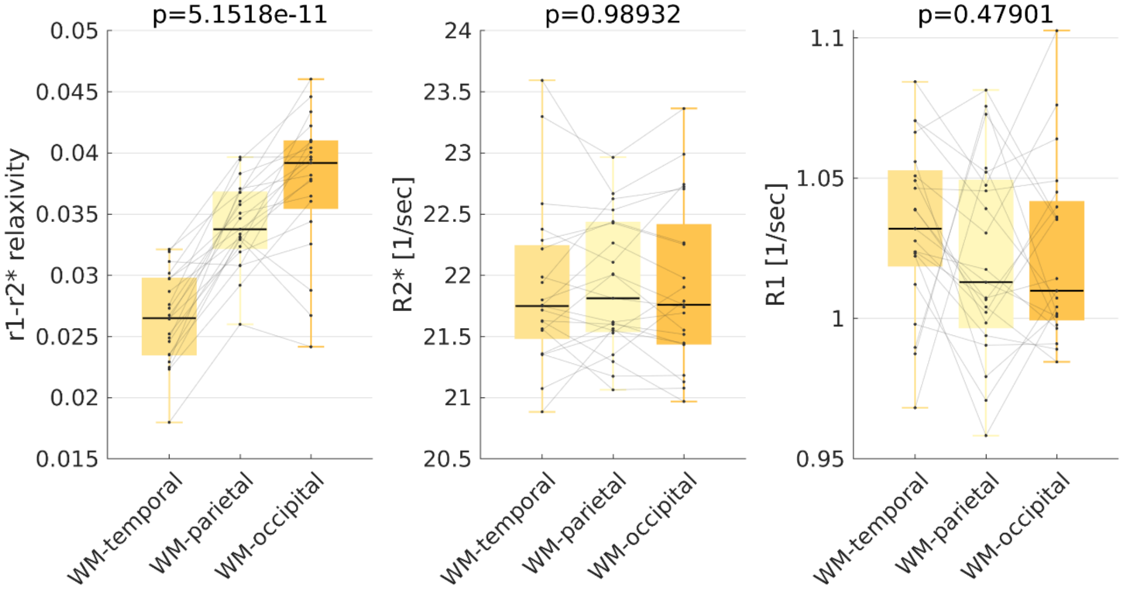


***Sup. Figure 20: The contrast of the $r_1$-$r_2^*$ relaxivity in white-matter.*** *From left to right, $r_1$-$r_2^*$ relaxivity, $R_2^*$ and $R_1$ for three different white-matter (WM) ROIs (temporal, parietal and occipital). We can separate these three WM ROIs with the $r_1$-$r_2^*$ relaxivity but not with either $R_2^*$ or $R_1$. Boxes represent the variation in the MRI parameters across normal subjects (age 27±2, N = 21). Lines connect between measurements of the same subject. The 50$^{th}$ percentile (horizontal black lines) 25th and 75th percentiles (box edges) and extreme data points (whiskers) are shown for each box. p-values are for one-way ANOVA test.*

## Supplementary Figure 21

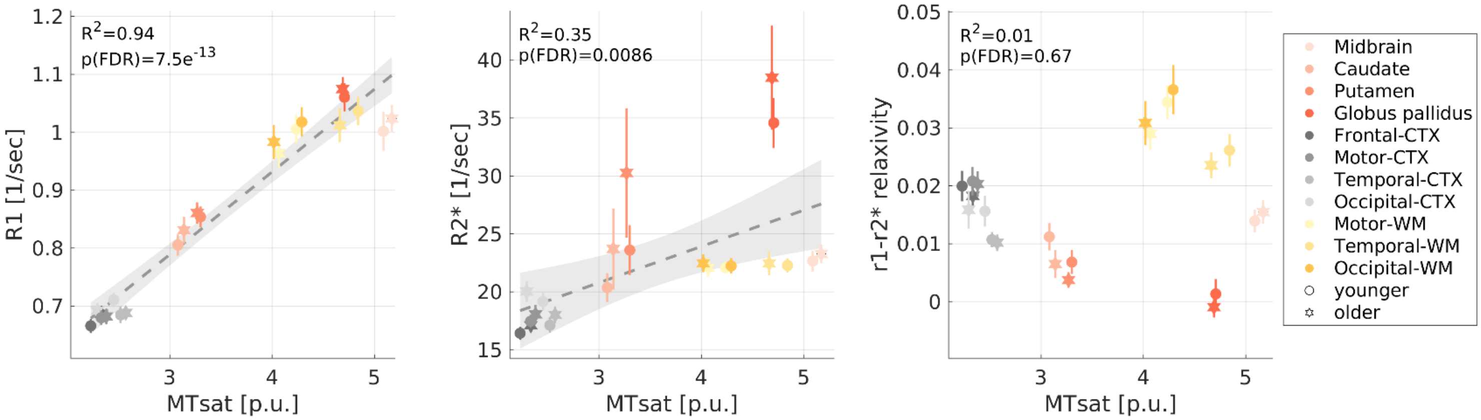


***Sup. Figure 21: The correlations of MRI parameters with MTsat.*** *The qMRI measurement of the magnetization transfer saturation (MTsat) vs. $R_1$, $R_2^*$ and the $r_1$-$r_2^*$ relaxivity measured in vivo across younger (aged 23-63 years, N =26) and older (aged 65-77 years, N=13) subjects (different marker shapes) in 10 brain regions (different colors). Data points show mean values and error bars show the mean absolute deviation across subjects. Unlike $R_1$ and $R_2^*$, the $r_1$-$r_2^*$ relaxivity is not significantly correlated with MTsat. p-values are for one-sided F-test corrected for multiple comparisons (FDR).*

## Supplementary Figure 22

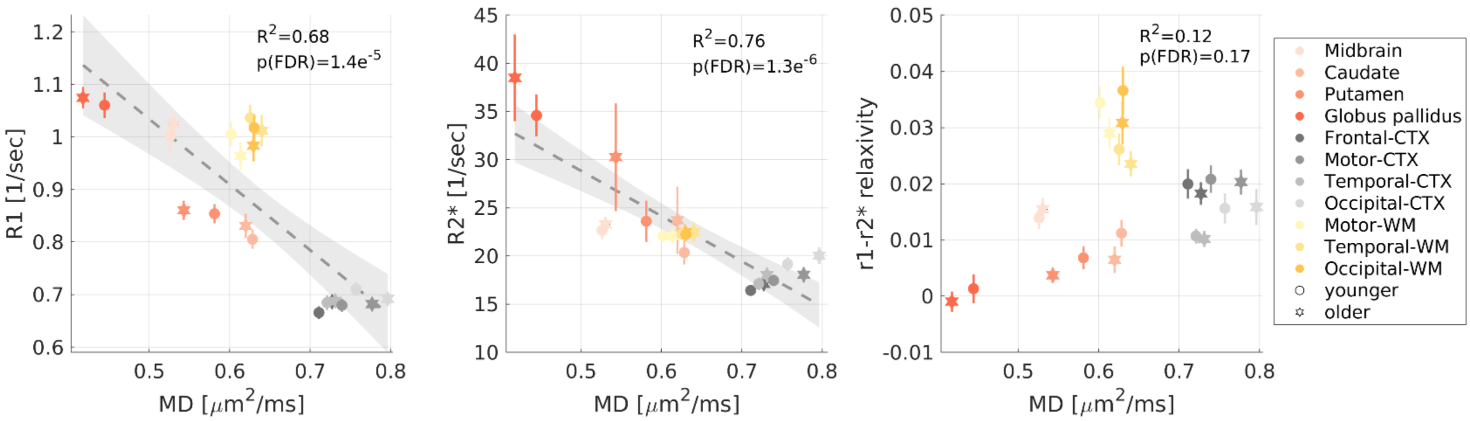


***Sup. Figure 22: The correlations of MRI parameters with MD.*** *The qMRI measurement of the mean diffusivity (MD) measured in vivo across younger (aged 23-63 years, N =25) and older (aged 65-77 years, N=12) subjects vs. $R_1$, $R_2^*$ and the $r_1$-$r_2^*$ relaxivity measured in vivo across younger (aged 23-63 years, N =26) and older (aged 65-77 years, N=13) subjects (different marker shapes) in 10 brain regions (different colors). Data points show mean values and error bars show the mean absolute deviation across subjects. Unlike $R_1$ and $R_2^*$, the $r_1$-$r_2^*$ relaxivity is not significantly correlated with MD. p-values are for one-sided F-test corrected for multiple comparisons (FDR).*

# Supplementary Section 9 (Supplementary Figure 23-31)
# Numerical simulations of the r1-r2* relaxivity.

The phantom experiments of ferritin and transferrin mixtures (Sup. Section 4) allowed us to establish a theoretical framework for the $r_1$-$r_2^*$ relaxivity in an *in vitro* environment where only the iron concentration changes, and the liposomal fraction mimicking the myelin is fixed ($[\Delta M] = 0$). In the brain, we estimate the $r_1$-$r_2^*$ relaxivity across all voxels of an anatomically-defined ROI. Within brain tissue ROIs, both the iron and the myelin concentration may vary[15].

Importantly, rearranging eq. S8 we find that the strength of the myelin effect on the $r_1$-$r_2^*$ relaxivity depends on how variable is the myelin content within an ROI relative to how variable is the iron content ($\frac{[\Delta M]}{[\Delta iron]}$):

$$\text{S14)} \qquad \frac{\Delta R_1}{\Delta R_2^*} = \frac{\left(f * r_{(1,Tf)} + (1-f) r_{(1,Ft)}\right) + r_{(1,M)} \frac{[\Delta M]}{[\Delta iron]}}{\left(f * r_{(2,Tf)} + (1-f) r_{(2,Ft)}\right) + r_{(2,M)} \frac{[\Delta M]}{[\Delta iron]}}$$

In order to evaluate how the $r_1$-$r_2^*$ relaxivity is modulated by the molecular iron environment and by the myelin and iron variabilities within an ROI in the brain, we performed a set of numerical simulations. In these simulations, similar to the *in vitro* mixtures experiments, the transferrin-ferritin fraction ($f = \frac{[Tf]}{[Ft]+[Tf]}$) represents an example for a feature of the molecular iron environment which should affect the $r_1$-$r_2^*$ relaxivity.

In these analyses we aim to simulate realistic concentrations of ferritin, transferrin and myelin, in order to achieve brain-like $R_1$ and $R_2^*$ values. Next, we follow our analysis pipeline; binning of the $R_2^*$ and $R_1$ measurements, excluding bins with small number of voxels, and assessing the $r_1$-$r_2^*$ relaxivity across the binned values. By varying the simulated content of the myelin and iron compounds we test to what extent each biological source contributes to the measurement of the $r_1$-$r_2^*$ relaxivity. First, we will examine our hypothesis that changes in the molecular iron environment, reflected by the transferrin-ferritin fraction, but not in the iron concentration, affect the $r_1$-$r_2^*$ relaxivity. We will then evaluate how the $r_1$-$r_2^*$ relaxivity is modulated by myelin. We will show that non-physiological conditions are required in order for the myelin by itself to fully explain the $r_1$-$r_2^*$ relaxivity changes measured in the brain.

Each numerical simulation was designed to mimic an ROI in the brain containing 1M voxels, with a fixed transferrin-ferritin fraction ($f$) across all voxels and varying myelin, transferrin and ferritin concentrations (Sup. Table 1).

| *Parameter* | *Value* | *Estimation method* |
|---|---|---|
| *Transferrin-ferritin fraction ($f$)* | 0.1 or 0.2 | Based on literature values[16–18]. |
| *$R_1$-ferritin relaxivity ($r_{(1,Ft)}$)* | 0.067 [($sec^{-1}$)/(mg/wet ml)] | *In vitro* linear dependency of $R_1$ on ferritin concentration |
| *$R_2^*$-ferritin relaxivity ($r_{(2,Ft)}$)* | 11.2 [($sec^{-1}$)/(mg/wet ml)] | *In vitro* linear dependency of $R_2^*$ on ferritin concentration |
| *$R_1$-transferrin relaxivity ($r_{(1,Tf)}$)* | 0.026 [($sec^{-1}$)/(mg/wet ml)] | *In vitro* linear dependency of $R_1$ on transferrin concentration |
| *$R_2^*$-transferrin relaxivity ($r_{(2,Tf)}$)* | 0.13 [($sec^{-1}$)/(mg/wet ml)] | *In vitro* linear dependency of $R_2^*$ on transferrin concentration |
| *Transferrin concentration ($[Tf]$)* | 0.025±0.025 [mg/wet ml] | Median is based on literature values[16–18]. Range across voxels was set so that the total change in $R_2^*$ will mimic the physiological change of 6-12 [1/sec] (**Sup. Figure**) |
| *Ferritin concentration ($[Ft]$)* | $\left(\frac{1}{f}-1\right)[Tf]$ | Set to satisfy the requirement for fixed transferrin-ferritin fraction ($f$) across all voxels. |
| *Myelin concentration in WM ($[M]_{WM}$)* | 0.29±0.047 [fraction] | Brain *in vivo* MTV values |
| *Myelin concentration in GM ($[M]_{GM}$)* | 0.19±0.043 [fraction] | Brain *in vivo* MTV values |
| *$R_2^*$-myelin relaxivity ($r_{(2,M)}$)* | 38.8 [($sec^{-1}$)/fraction] | Brain *in vivo* linear dependency of $R_2^*$ on MTV |
| *$R_1$-myelin relaxivity ($r_{(1,M)}$)* | 2.6 [($sec^{-1}$)/fraction] | Brain *in vivo* linear dependency of $R_1$ on MTV |

***Sup. Table 1: Simulation parameters.***

We synthetically generated $R_1$ and $R_2^*$ values for each voxel based on eq. S4-S5. The relaxivities of ferritin and transferrin ($r_{(1/2,Ft)}$, $r_{(1/2,Tf)}$) were taken from the results of our phantom experiments (Figure 2). In order to generate simulations that are as realistic as possible, the rest of the parameters were adapted from the human brain. The mean ferritin and transferrin concentrations (across all voxels of the ROI) were estimated based on post-mortem findings[16–18]. The myelin characteristics were simulated based on the qMRI measurement of the macromolecular tissue volume (MTV)[19], defined as 1-water fraction, which was shown to approximate the myelin content [20–24]. The myelin relaxivity ($r_{(1/2,M)}$) is defined as the dependency of relaxation rates on the myelin concentration[13]. We estimated the myelin relaxivity as the

linear dependency of $R_1$ and $R_2^*$ on MTV, averaged across 16 ROIs in the brains of 21 young subjects. In order to assess the changes in myelin content within brain ROIs ($[\Delta M]$), we calculated the range of MTV values within white-matter (WM) or gray-matter (GM) regions averaged across 8 ROIs in the brains of 21 young subjects (Sup. Figure 23). Finally, the changes in ferritin and transferrin concentrations within brain ROIs ($[\Delta iron]$) were determined based on the range of $R_2^*$ values within 16 WM and GM regions in the brains of 21 young subjects. We assumed that the changes in $R_2^*$ not explained by MTV are related to changes in iron concentration (Sup. Figure 24):

$$\text{S15)} \quad \Delta(iron)R_2^* = \Delta(total)R_2^* - r_{(2,M)}[\Delta M]$$

We found that the total change in $R_2^*$ within ROIs in the human brain is on average $\Delta(total)R_2^*$=9.0 1/sec, from which about 61% ($\Delta(iron)R_2^*$=5.6 1/sec) could be related to changes in iron concentration. Therefore, the simulated variably in the ferritin and transferrin concentrations were set to satisfy this requirement.

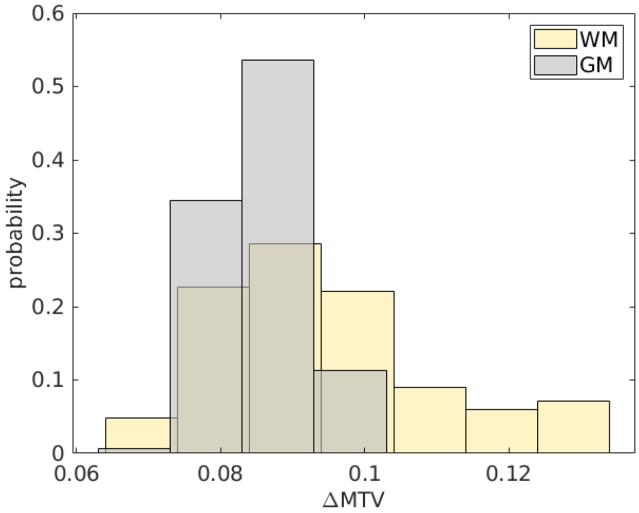


***Sup. Figure 23: Change in MTV values ($[\Delta MTV]$) within white-matter (WM) or gray-matter (GM) regions.*** *ΔMTV values for gray matter (GM) and white matter (WM) are presented across 16 ROIs in the brains of 21 young subjects. For each ROI, we extracted the MTV values from all voxels and pooled them into 36 bins spaced equally between 0.05 and 0.40 [fraction]. We removed any bins in which the number of voxels was smaller than 4% of the total voxel count in the ROI. This was done so that the calculation will not be heavily affected by outlier voxels with extreme values. The median MTV of each bin was computed, and the difference between the highest and lowest binned MTV values was set as ΔMTV ([fraction]) in the ROI.*

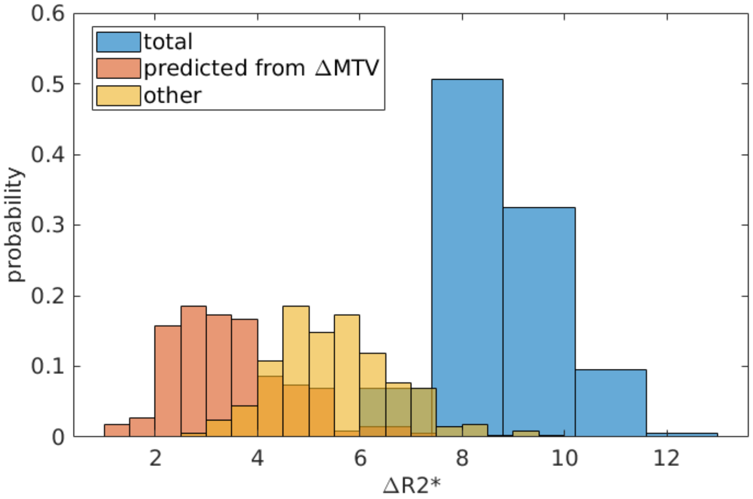


***Sup. Figure 24: Myelin- and iron- related changes in $R_2^*$ within brain regions.*** *Total change in $R_2^*$ values ($[\Delta R_2^*]$) within brain regions (blue histogram) is presented across 16 ROIs in the brains of 21 young subjects. For each ROI, we extracted the $R_2^*$ values from all voxels and pooled them into 36 bins spaced equally between 0 and 50. We removed any bins in which the number of voxels was smaller than 4% of the total voxel count in the ROI. This was done so that the calculation will not be heavily affected by outlier voxels with extreme values. The median $R_2^*$ of each bin was computed, and the difference between the highest and lowest binned $R_2^*$ values was set as $\Delta R_2^*$ in the ROI (in [1/sec]).* ***$R_2^*$ changes related to myelin (orange histogram)*** *were estimated based on MTV; the change in $R_2^*$ predicted from the change in MTV (ΔMTV) within each ROI was calculated as the linear dependency of $R_2^*$ on MTV in the ROI multiplied by ΔMTV in the ROI ($r_{(2,M)}[\Delta M]$).* ***$R_2^*$ changes related to iron (yellow histogram)*** *were estimated as the change in $R_2^*$ not explained by the change in MTV.*

An example result of the numerical simulation is presented in Sup. Figure 25a. It is evident that both the changes in myelin concentration across voxels of the simulated ROI (represented by different colors) and the changes in ferritin and transferrin concentrations across voxels (represented by the symbols size) affect the measured $r_1$-$r_2^*$ relaxivity. In our analysis pipeline we first bin the $R_1$ and $R_2^*$ values within the ROI and next calculate $r_1$-$r_2^*$ relaxivity over the binned values. Therefore, the variability in $R_1$ for a given $R_2^*$ bin is collapsed to an average $R_1$ value (black data points). We assume that this approach eliminates some of the variability related to myelin. We hypothesized that the $r_1$-$r_2^*$ relaxivity is sensitive to the iron environment. Indeed, in our simulations we find that by setting different physiological transferrin-ferritin fractions and leaving the myelin parameters constant, the $r_1$-$r_2^*$ relaxivity changes considerably (Sup. Figure 25).

In addition, we hypothesized that the $r_1$-$r_2^*$ relaxivity is less sensitive to the iron concentration and is more sensitive to the iron environment. To test this, we run two numerical simulations with the same transferrin-ferritin fraction but with different transferrin and ferritin concentrations. As expected, $R_1$ and

$R_2^*$ values changed with increased ferritin and transferrin concentrations but the $r_1$-$r_2^*$ relaxivity did not change (Sup. Figure 26). This indicates that the $r_1$-$r_2^*$ relaxivity measurement is less sensitive to absolute changes in the iron concentration and is sensitive to the interplay between iron compounds, reflected in the simulations by the transferrin-ferritin fraction. In the brain, changes in the myelin content between GM and WM are known to substantially affect the measurements of $R_1$ and $R_2^*$[4,7–12,25,26]. To test the potential contribution of the myelin to the $r_1$-$r_2^*$ relaxivity, we changed the myelin concentration in our simulation while keeping the rest of the parameters fixed. Setting the myelin concentration to that typical for GM or WM (as estimated *in vivo* by MTV) led to considerable changes in $R_1$ and $R_2^*$, but did not produce any change in the $r_1$-$r_2^*$ relaxivity (Sup. Figure 27).

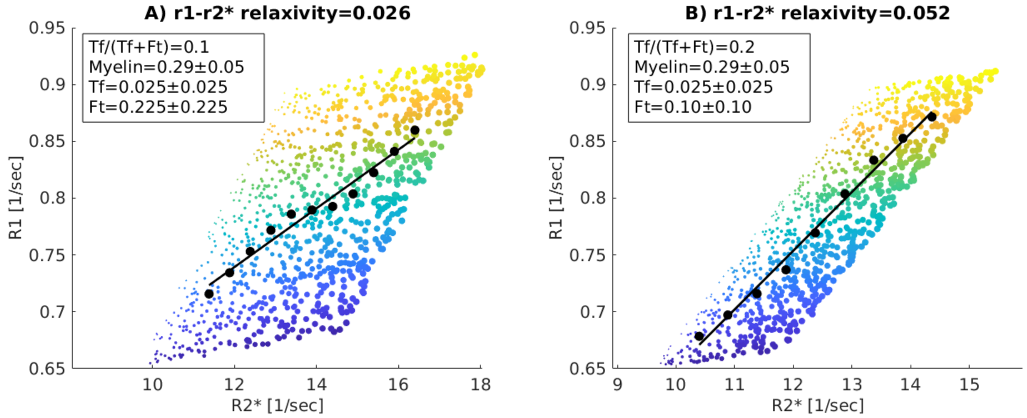


***Sup. Figure 25: The $r_1$-$r_2^*$ relaxivity in two simulated ROIs with different transferrin-ferritin fractions (Tf/(Tf+Ft))****; (A) the transferrin-ferritin fraction is 0.1; (B) the transferrin-ferritin fraction is 0.2. Each figure shows the dependency of $R_1$ on $R_2^*$ for 1,000 representative simulated voxels. The colors of the data points indicate the variability in myelin concentration across voxels, and their sizes indicate the variability in iron compounds concentration across voxels (the simulated concentrations are shown in the text box, myelin is in units of [fraction] as MTV, transferrin and ferritin are in units of [mg/ml]). As in our in vivo pipeline, $R_2^*$ and $R_1$ values were binned (black data points represent the bins' median), and a linear fit was calculated (black line). The slopes of the linear fit (shown in the title) represent the dependency of $R_1$ on $R_2^*$ ($r_1$-$r_2^*$ relaxivity) and vary with the transferrin-ferritin fraction.*

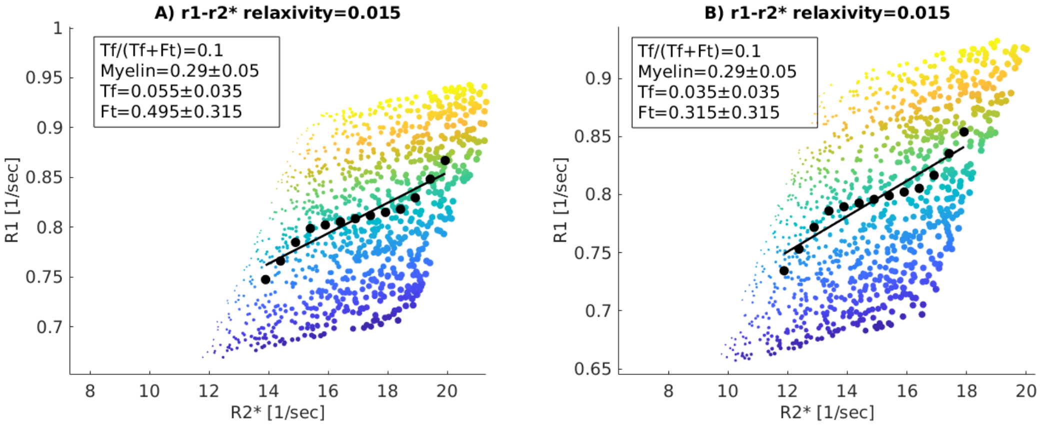


***Sup. Figure 26: The** $r_1$-$r_2^*$ **relaxivity in two simulated ROIs with different transferrin and ferritin concentrations and a similar transferrin-ferritin fraction (Tf/(Tf+Ft));*** *(A) a higher transferrin and ferritin concentrations and a transferrin-ferritin fraction of 0.1; (B) a lower transferrin and ferritin concentrations and a transferrin-ferritin fraction of 0.1. Each figure shows the dependency of* $R_1$ *on* $R_2^*$ *for 1,000 representative simulated voxels. The colors of the data points indicate the variability in myelin concentration across voxels, and their sizes indicate the variability in iron compounds concentration across voxels (the simulated concentrations are shown in the text box, myelin is in units of [fraction] as MTV, transferrin and ferritin are in units of [mg/ml]). As in our in vivo pipeline,* $R_2^*$ *and* $R_1$ *values were binned (black data points represent the bins' median), and a linear fit was calculated (black line). The slopes of the linear fit (shown in the title) represent the dependency of* $R_1$ *on* $R_2^*$ *(*$r_1$-$r_2^*$ *relaxivity) and do vary with the change in transferrin and ferritin concentrations.*

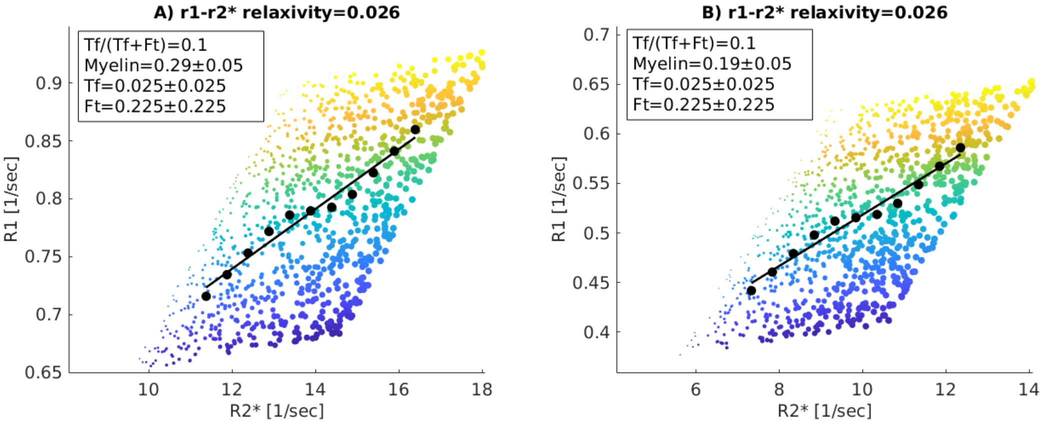


***Sup. Figure 27: The** $r_1$-$r_2^*$ **relaxivity in two simulated ROIs with different myelin concentrations and similar transferrin-ferritin fractions (Tf/(Tf+Ft))****; (A) a higher myelin concentration and transferrin-ferritin fraction of 0.1; (B) a lower myelin concentration and transferrin-ferritin fraction of 0.1. Each figure shows the dependency of* $R_1$ *on* $R_2^*$ *for 1,000 representative simulated voxels. The colors of the data points indicate the variability in myelin concentration across voxels, and their sizes indicate the variability in iron compounds concentration across voxels (the simulated concentrations are shown in the text box, myelin is in units of [fraction] as MTV, transferrin and ferritin are in units of [mg/ml]). As in our in vivo pipeline,* $R_2^*$ *and* $R_1$ *values were binned (black data points represent the bins' median), and a linear fit was*

*calculated (black line). The slopes of the linear fit (shown in the title) represent the dependency of $R_1$ on $R_2^*$ ($r_1$-$r_2^*$ relaxivity) and does not vary with the change in myelin concentration.*

The theoretical formulation presented here indicates that it is not the myelin concentration, but the variability in myelin within an ROI ($[\Delta M]$), that is important for determining the $r_1$-$r_2^*$ relaxivity (eq. S14). However, estimating the variability in myelin within ROIs *in vivo* based on the myelin marker MTV, we find that it only explains ~30% of the variation in the *in vivo* $r_1$-$r_2^*$ relaxivity measurements across the brain (Sup. Figure 28). In the simulations, setting both the range and concentration of myelin to the ones typical for WM or GM (as estimated by MTV, Sup. Figure 23), slightly changed the $r_1$-$r_2^*$ relaxivity (0.002, Sup. Figure 29). Importantly, changing the transferrin-ferritin fraction between the physiological values of 0.1-0.2 led to a change of 0.026 in the $r_1$-$r_2^*$ relaxivity (Sup. Figure 25). Therefore, the simulated changes related to the molecular iron environment were one order of magnitude bigger (Sup. Figure 25).

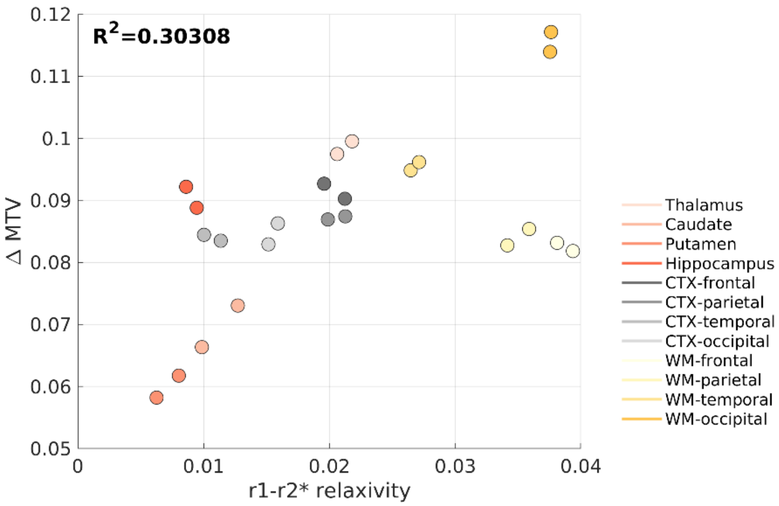


***Sup. Figure 28: The in vivo estimates of the variability in myelin within ROIs vs. the in vivo $r_1$-$r_2^*$ relaxivity.*** *The average estimated myelin variability within ROIs, computed across the brains of 21 young subjects, vs. the average $r_1$-$r_2^*$ relaxivity for the same subjects. Brain regions are presented in different colors (each ROI has left and right hemisphere estimates). The variability in myelin within brain regions was estimated based on the variability in MTV across voxels of each ROI (ΔMTV, y-axis). For each ROI, we extracted the MTV values from all voxels and pooled them into 36 bins spaced equally between 0.05 and 0.40 [fraction]. We removed any bins in which the number of voxels was smaller than 4% of the total voxel count in the ROI. This was done so that the calculation will not be heavily affected by outlier voxels with extreme values. The median MTV of each bin was computed, and the difference between the highest and lowest binned MTV values was set as ΔMTV ([fraction]) in the ROI.*

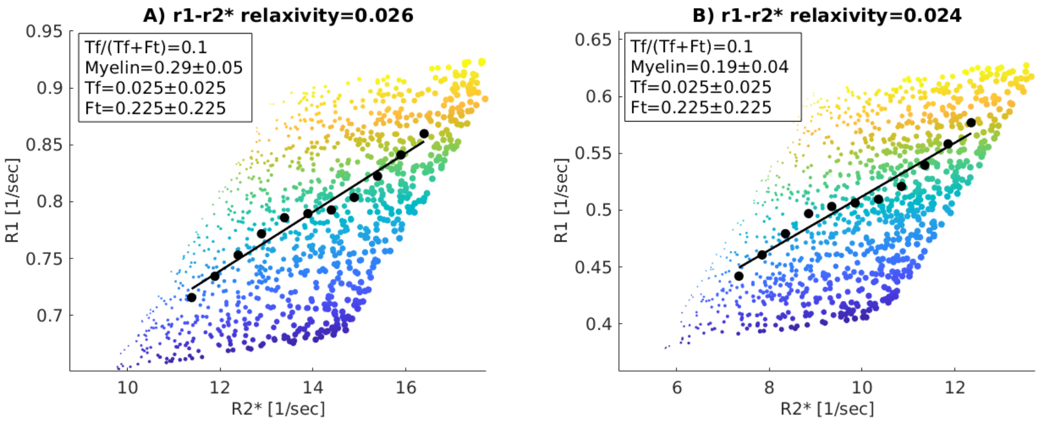


***Sup. Figure 29: The*** *$r_1$-$r_2^*$* ***relaxivity in two simulated ROIs with different myelin concentrations, different ranges of myelin concentrations ($[\Delta M]$), and similar transferrin-ferritin fractions (Tf/(Tf+Ft))****; (A) WM; a higher myelin concentration and a larger range of myelin variability, the transferrin-ferritin fraction is 0.1; (B) GM; a lower myelin concentration and a lower range of myelin variability, the transferrin-ferritin fraction is 0.1. Each figure shows the dependency of $R_1$ on $R_2^*$ for 1,000 representative simulated voxels. The colors of the data points indicate the variability in myelin concentration across voxels, and their sizes indicate the variability in iron compounds concentration across voxels (the simulated concentrations are shown in the text box, myelin is in units of [fraction] as MTV, transferrin and ferritin are in units of [mg/ml]). As in our in vivo pipeline, $R_2^*$ and $R_1$ values were binned (black data points represent the bins' median), and a linear fit was calculated (black line). The slopes of the linear fit (shown in the title) represent the dependency of $R_1$ on $R_2^*$ ($r_1$-$r_2^*$ relaxivity) and vary slightly with the range of myelin variability ($[\Delta M]$).*

We further tested what are the myelin properties that would generate similar $r_1$-$r_2^*$ relaxivity effect as the effect observed when changing the transferrin-ferritin fraction (a change of 0.026 in the $r_1$-$r_2^*$ relaxivity, Sup. Figure 25). As changing the myelin concentration does not change the $r_1$-$r_2^*$ relaxivity (Sup. Figure 27), we changed the variability in myelin concentration within the ROI ($[\Delta M]$, Sup. Figure 29). We found that in order to generate a change of 0.026 in the $r_1$-$r_2^*$ relaxivity only through myelin-related changes (when the iron-related properties are fixed), the variability in MTV within the simulated ROI should be in the order of 0.187 [fraction] (Sup. Figure 30). Evaluating the *in vivo* variability in MTV within WM, GM and subcortical ROIs across 21 young subjects, the typical variability is ~ 0.09 [fraction], and the most extreme variability that was measured was 0.13 [fraction] (in the WM, Sup. Figure 23). Even this atypical value is still much lower than that required to generate a change of 0.026 in the $r_1$-$r_2^*$ relaxivity (0.187 [fraction] in MTV). Thus, while the variability in myelin within ROIs can affect the $r_1$-$r_2^*$ relaxivity, there are no physiological myelin properties that would fully explain the $r_1$-$r_2^*$ relaxivity effect measured in the brain.

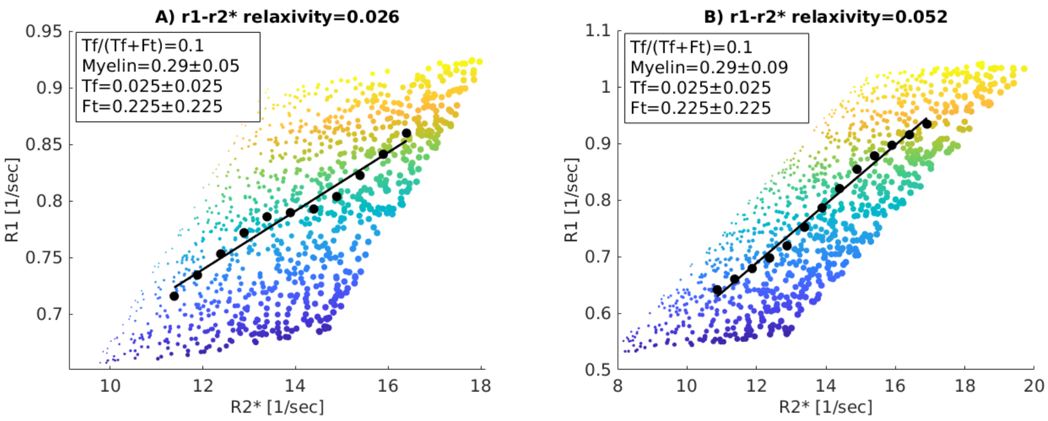


***Sup. Figure 30: The** $r_1$-$r_2^*$ **relaxivity in two simulated ROIs with different extreme ranges of myelin concentrations ([$\Delta M$]) and similar transferrin-ferritin fractions (Tf/(Tf+Ft));** (A) a physiological range of myelin variability, and a transferrin-ferritin fraction of 0.1; (B) the range of myelin variability is almost doubled, and the transferrin-ferritin fraction is 0.1. Each figure shows the dependency of $R_1$ on $R_2^*$ for 1,000 representative simulated voxels. The colors of the data points indicate the variability in myelin concentration across voxels, and their sizes indicate the variability in iron compounds concentration across voxels (the simulated concentrations are shown in the text box, myelin is in units of [fraction] as MTV, transferrin and ferritin are in units of [mg/ml]). As in our in vivo pipeline, $R_2^*$ and $R_1$ values were binned (black data points represent the bins' median), and a linear fit was calculated (black line). The slopes of the linear fit (shown in the title) represent the dependency of $R_1$ on $R_2^*$ ($r_1$-$r_2^*$ relaxivity) and change considerably under this condition of extreme variability in myelin concentration ([$\Delta M$]).*

The iron and myelin contents of brain tissue are tightly related, as iron is required for the formation of myelin[4]. To test how this affects the $r_1$-$r_2^*$ relaxivity measurement, we simulated a case where iron and myelin are completely correlated. Importantly, even in this extreme case, different transferrin-ferritin fractions exhibited different $r_1$-$r_2^*$ relaxivity (Sup. Figure 31).

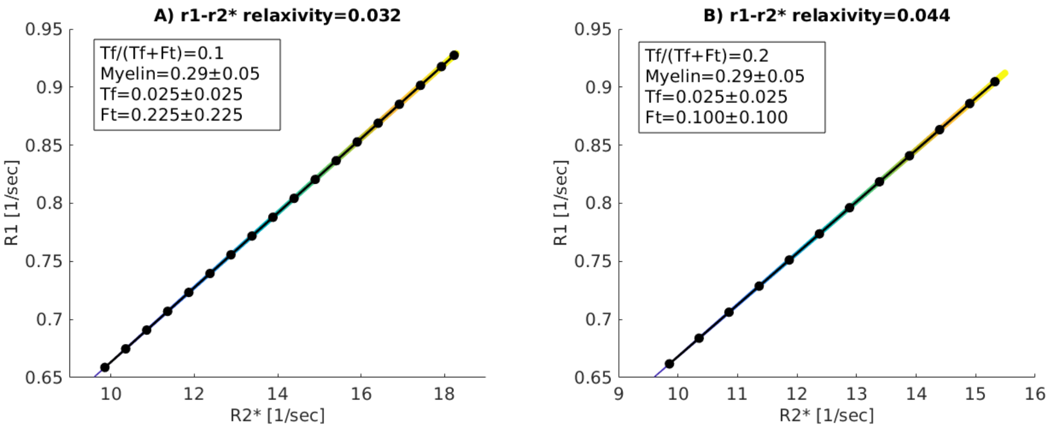


***Sup. Figure 31: The** $r_1$-$r_2^*$ **relaxivity in two simulated ROIs with different transferrin-ferritin fractions (Tf/(Tf+Ft)) and a correlation between iron and myelin concentrations across voxels;** (A) The*

*transferrin-ferritin fraction is 0.1; (B) the transferrin-ferritin fraction is 0.2. In both A and B iron and myelin are correlated. Each figure shows the dependency of $R_1$ on $R_2^*$ for 1,000 representative simulated voxels. The colors of the data points indicate the variability in myelin concentration across voxels, and their sizes indicate the variability in iron compounds concentration across voxels (the simulated concentrations are shown in the text box, myelin is in units of [fraction] as MTV, transferrin and ferritin are in units of [mg/ml]). As in our in vivo pipeline, $R_2^*$ and $R_1$ values were binned (black data points represent the bins' median), and a linear fit was calculated (black line). The slopes of the linear fit (shown in the title) represent the dependency of $R_1$ on $R_2^*$ ($r_1$-$r_2^*$ relaxivity) and change considerably with the transferrin-ferritin fraction even when iron and myelin are correlated.*

To conclude, we simulated a brain-like environment in order to test the biological sources affecting the $r_1$-$r_2^*$ relaxivity measurement. We found that physiological changes in the molecular iron environment, represented in the simulations by the ferritin-transferrin fraction, led to considerable changes in the $r_1$-$r_2^*$ relaxivity (0.026). This effect could not be attributed to the absolute concentrations of iron compounds, only to the ratio between them. To estimate whether the effect of changing the molecular iron environment is measurable *in vivo*, we assessed the detection limit of the $r_1$-$r_2^*$ relaxivity measurement using scan-rescan experiments (Sup. Figure 14). We found that the changes in the $r_1$-$r_2^*$ relaxivity simulated by different physiological transferrin-ferritin ratios are well above the detection limit of this MRI measurement *in vivo* (MAE~0.0035). Importantly, our arguments regarding the sensitivity of the $r_1$-$r_2^*$ relaxivity to the molecular iron environment are demonstrated in the simulations based on the example of the transferrin-ferritin ratio. However, it is evident from our theoretical formulation (eq. S14) that the variability in iron concentration within an ROI contributes to the $r_1$-$r_2^*$ relaxivity as well. Therefore, while the transferrin-ferritin ratio was used as an example, other features of the iron environment such as the spatial variability of iron compounds, their binding capacities and aggregate sizes, could affect the $r_1$-$r_2^*$ relaxivity as well. Next, we confirmed that changes in the myelin concentration affect the measurements of $R_1$ and $R_2^*$, but not the $r_1$-$r_2^*$ relaxivity. The myelin variability within an ROI can affect the $r_1$-$r_2^*$ relaxivity. However, we found that *in vivo* estimates of this myelin characteristic explain only 30% of the variation in the *in vivo* $r_1$-$r_2^*$ relaxivity measurement across the brain. In the simulation, setting the myelin variability within an ROI to typical GM and WM values led to a slight change in the $r_1$-$r_2^*$ relaxivity. However, we found that unrealistic myelin variability is required in order to produce the $r_1$-$r_2^*$ relaxivity effect observed for realistic changes in the transferrin-ferritin fraction. Therefore, while the myelin substantially affects the measurements of $R_1$ and $R_2^*$, it is not the main component governing the

measurement of the $r_1$-$r_2^*$ relaxivity, and under physiological conditions it cannot by itself explain the measured variability in the $r_1$-$r_2^*$ relaxivity across the brain.

## Supplementary Section 10 (Supplementary Figures 32-34)
## Comparison of the r1-r2* relaxivity and T1w/T2w.

The semi-quantitative T1w/T2w imaging is widely used as a myelin marker[27]. Both the $r_1$-$r_2^*$ relaxivity and the T1w/T2w approaches represent combinations of transverse and longitudinal relaxation. Due to the underlying similarities between these methods we wanted to verify that the $r_1$-$r_2^*$ relaxivity is less sensitive to myelin compared to T1w/T2w. Mathematically, it can be shown that the T1w/T2w enhances the myelin contribution, while the $r_1$-$r_2^*$ relaxivity reduces the myelin contribution. T1w is proportional to $R_1$, and T2w is proportional to 1/R2. Therefore, T1w/T2w is proportional to $R_1$*$R_2$. Assuming both $R_1$ and $R_2$ are linearly related to myelin, then T1w/T2w is proportional to myelin squared (as argued by Glasser et al[27]). On the contrary, the $r_1$-$r_2^*$ relaxivity (i.e., the slope of the $R_1$-$R_2^*$ linear fit) represents the change in $R_1$ relative to the change in $R_2^*$ ($\Delta R_1/\Delta R_2^*$), and is therefore less sensitive to the magnitude of these relaxation rates and more sensitive to their shared variation. While the myelin concentration has a large effect on the magnitudes of $R_1$ and $R_2^*$, its effect of the shared variation of $R_1$ and $R_2^*$ ($\Delta R_1/\Delta R_2^*$) is minimal. $\Delta R_1/\Delta R_2^*$ is mostly related to the variability in myelin concentration within ROIs ($\Delta M$)

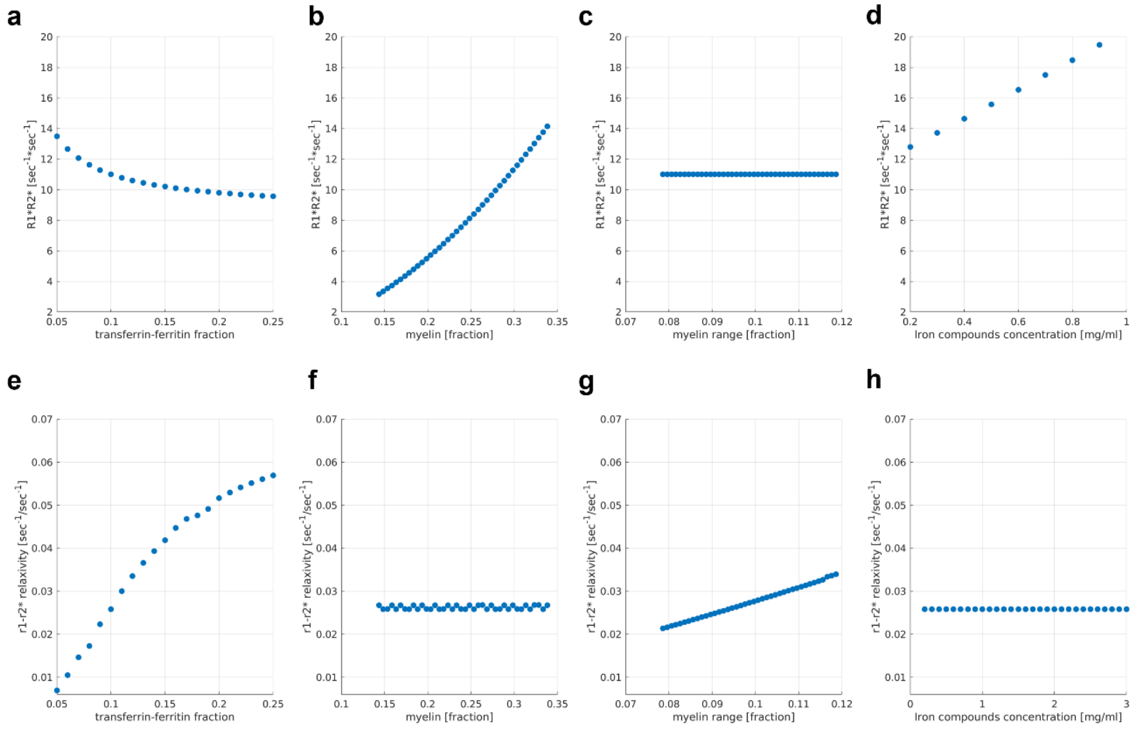

***Sup. Figure 32:** Simulations of the sensitivity of R1*R2* (a-d) and r1-r2* relaxivity (e-h) to different biophysical sources. The variability in R1*R2* and r1-r2* relaxivity was tested across the physiological ranges of transferrin-ferritin fraction (a,e), myelin concentration (b,f), myelin range (ΔM , c,g) and iron compounds concentrations (d,h). Simulations were performed under the same framework as in Supplementary Section . In each simulation we changed one biological component while keeping the rest fixed.*

(Sup. Section 9). We show in simulations that non-physiological values of ΔM are required to produce the r1-r2* relaxivity values measured in the brain. To demonstrate the differences between T1w/T2w and the $r_1$-$r_2^*$ relaxivity, we performed simulations of $r_1$-$r_2^*$ relaxivity and $R_1{*}R_2^*$ (similar to $R_1{*}R_2$ which is proportional to T1w/T2w). We tested these parameters across the physiological ranges of transferrin-ferritin fraction, myelin concentration, myelin range (ΔM) and iron compounds concentrations. In each simulation we changed one biological component while keeping the rest fixed. These simulations follow the same framework presented in Sup. Section 9. Sup. Figure 32 demonstrates that $R_1{*}R_2^*$ changes mostly with the myelin and iron concentrations, but also with the transferrin-ferritin fraction. On the other hand, the $r_1$-$r_2^*$ relaxivity changes mostly with the transferrin-ferritin fraction. It also changes with the myelin variability (ΔM), but to a smaller extent (Sup. Section 9). Nevertheless, the simulated $r_1$-$r_2^*$ relaxivity does not change with the myelin and iron concentrations.

Moreover, we provide a comparison between the $r_1$-$r_2^*$ relaxivity and $R_1{*}R_2^*$ *in vivo*. Sup. Figure 33 shows that across subjects and brain regions, the $r_1$-$r_2^*$ relaxivity is different compared to $R_1{*}R_2^*$. Sup. Figure 34

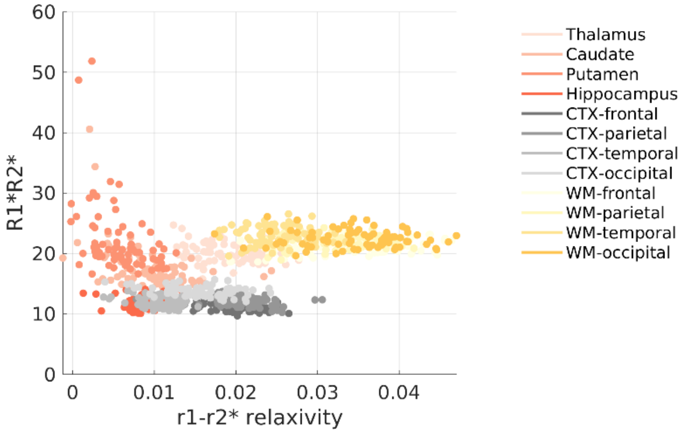


***Sup. Figure 33:** comparison between R1*R2* (y-axis) and the r1-r2* relaxivity (x-axis) across different brain areas (different colors) for all healthy human subjects (N=39).*

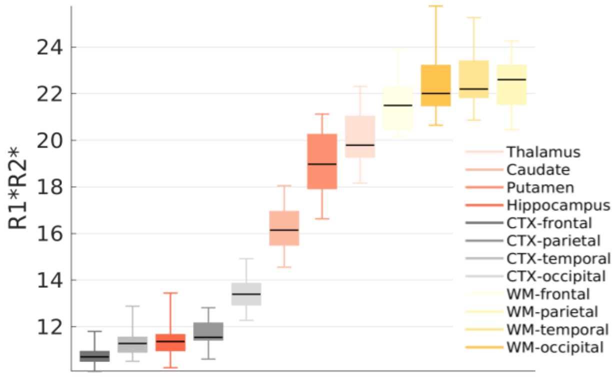


***Sup. Figure 34:** R1*R2* across the brain. The variation across young subjects (age 27±2, N = 21) in R1*R2* in different brain regions. The 25th, 50th and 75th percentiles and extreme data points are shown for each box.*

emphasizes that the contrast of $R_1{*}R_2^*$ across the brain is different from the $r_1$-$r_2^*$ relaxivity contrast (Figure 3b). Interestingly, the contrast between white-matter regions shown in the $r_1$-$r_2^*$ relaxivity (Figure

3b), is not present in $R_1*R_2^*$. To conclude, T1w/T2w images are similar in contrast to $R_1*R_2^*$. We show mathematically, in simulations and *in vivo*, that $R_1*R_2^*$ is different from the $r_1$-$r_2^*$ relaxivity, and more sensitive to myelin concentration.

## Supplementary Section 11 (Supplementary Figures 35-37)
## Comparison of the r1-r2* relaxivity and the R1/R2* ratio.

The voxel-wise division of $R_1$ and $R_2^*$ (the $R_1/R_2^*$ ratio) may provide a simple approximation of the $r_1$-$r_2^*$ relaxivity, which has the benefit of allowing higher spatial resolution. However, we demonstrate that the $R_1/R_2^*$ ratio has a different biophysical interpretation compared to the $r_1$-$r_2^*$ relaxivity. Mainly, the $R_1/R_2^*$ ratio depends on the relative magnitudes of $R_1$ and $R_2^*$. On the other hand, the slope of the $R_1$-$R_2^*$ linear fit (i.e. the $r_1$-$r_2^*$ relaxivity) represents the change in $R_1$ relative to the change in $R_2^*$ ($\Delta R_1/\Delta R_2^*$), and is therefore less sensitive to the magnitude of these relaxation rates and more sensitive to their shared variation. To demonstrate this point, we performed simulations of the $R_1/R_2^*$ ratio and the $r_1$-$r_2^*$ relaxivity across the physiological ranges of transferrin-ferritin fraction, iron compounds

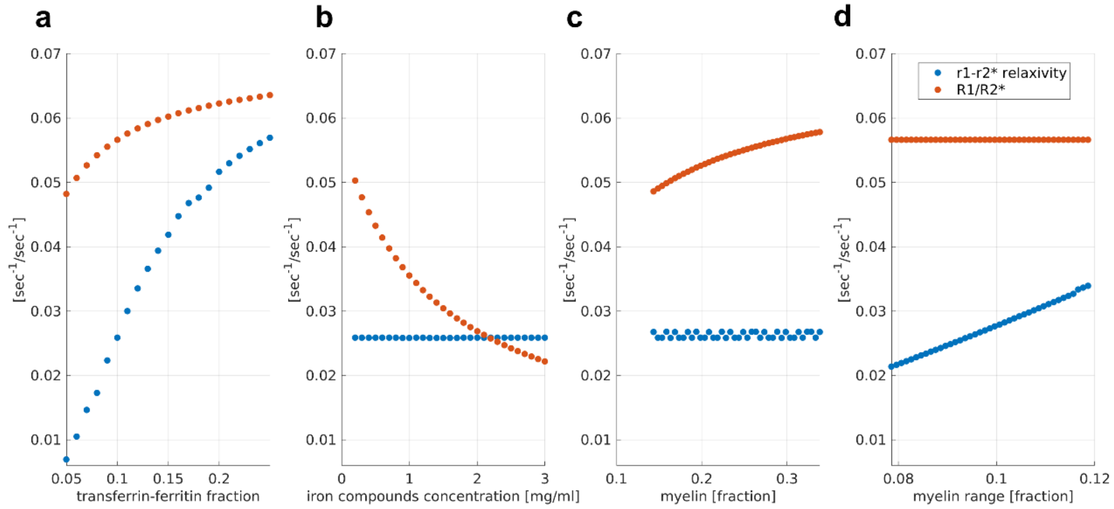


***Sup. Figure 35:** Simulations of the sensitivity of the R1/R2* ratio and the r1-r2* relaxivity to different biophysical sources. The variability in R1/R2* and r1-r2* relaxivity was tested across the physiological ranges of transferrin-ferritin fraction (a), iron compounds concentration (b), myelin concentration (c) and myelin variability (ΔM , d). In each panel only one biological property is changing while the rest are kept fixed. Simulations were performed under the same framework as in Sup. Section 9.*

concentration, myelin concentration and myelin variability (ΔM). In each simulation we changed one biological component while keeping the rest fixed. These simulations follow the same framework presented in Sup. Section 9. Sup. Figure 35 demonstrates that the $R_1/R_2^*$ ratio changes with several

physiological properties; the transferrin-ferritin fraction, the iron compounds concentration and the myelin concentration. On the other hand, the $r_1$-$r_2^*$ relaxivity changes mostly with the transferrin-ferritin fraction. It is also sensitive, but to a smaller degree, to changes in ΔM (Sup. Section 9). Changes in the myelin and the iron compounds concentrations do not affect the simulated $r_1$-$r_2^*$ relaxivity. Therefore, the $R_1/R_2^*$ ratio and the $r_1$-$r_2^*$ relaxivity have different biophysical sources.

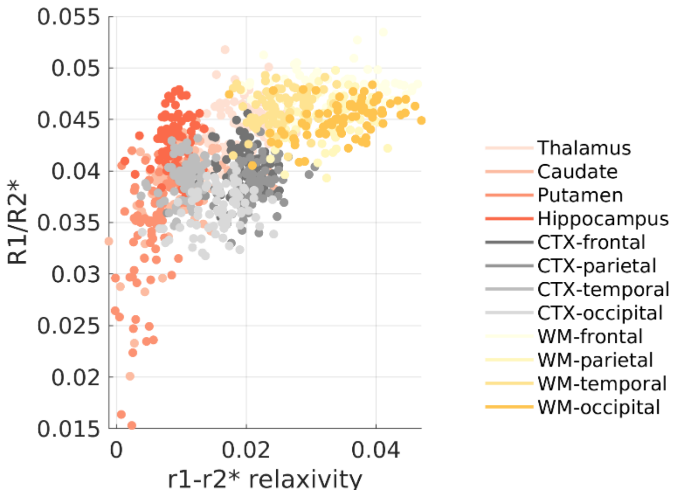


***Sup. Figure 36:*** *comparison between the R1/R2* ratio (y-axis) and the r1-r2* relaxivity (x-axis) across different brain areas (different colors) for all healthy human subjects (N=39).*

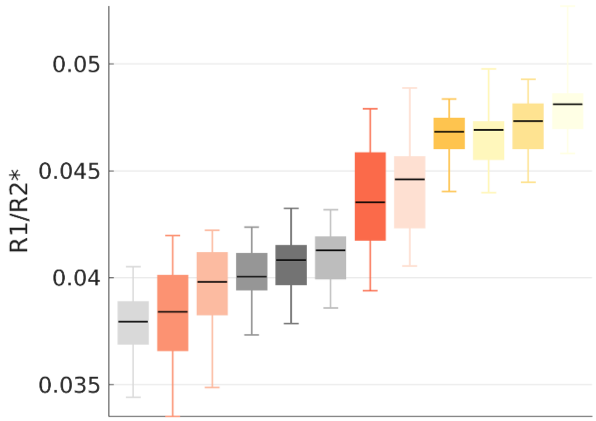


***Sup. Figure 37:*** *The R1/R2* ratio across the brain. The variation across young subjects (age 27±2, N = 21) in the R1/R2* ratio in different brain regions. The 25th, 50th and 75th percentiles and extreme data points are shown for each box.*

To further demonstrate the differences between the $r_1$-$r_2^*$ relaxivity and the $R_1/R_2^*$ ratio, we provide a comparison between these measurements *in vivo*. Sup. Figure 36 shows that across subjects and brain regions, the $r_1$-$r_2^*$ relaxivity is different compared to the $R_1/R_2^*$ ratio.

Sup. Figure 37 emphasizes that the contrast of the $R_1/R_2^*$ ratio across the brain is different from the $r_1$-$r_2^*$ relaxivity contrast (Figure 3b). Namely, the contrast between different white-matter regions that was observed in the $r_1$-$r_2^*$ relaxivity (Sup. Figure 20) vanishes in the $R_1/R_2^*$ ratio. The contrast between different gray-matter regions is also different between these measurements. Another example for the differences between the measurements is that the hippocampus, which has the second lowest $r_1$-$r_2^*$ relaxivity, is similar to the thalamus and is closer to white-matter regions in terms of its $R_1/R_2^*$ ratio.

These analyses validate that the $r_1$-$r_2^*$ relaxivity is inherently distinct from the $R_1/R_2^*$ ratio. It highlights the unique nature of the relaxivity measurement, which does not depend on the magnitude of the relaxation rates but rather on

their shared variation. This reduces the effect of the myelin and iron concentrations on the $r_1$-$r_2^*$ relaxivity, and enhances its sensitivity to the iron homeostasis.

## Supplementary Table 2

| | | $R^2$ | P-VALUE | F-STATISTIC | DF |
|---|---|---|---|---|---|
| **MTV** | R1 | 0.97 | 3.48E-16 | 802.5 | 20 |
| | r1-r2* relaxivity | 0.021 | 0.60 | 0.44 | 20 |
| | R2* | 0.46 | 0.0019 | 17.17 | 20 |
| **IRON** | R1 | 0.20 | 6.77E-02 | 5.11 | 20 |
| | r1-r2* relaxivity | 0.37 | 0.0076 | 11.90 | 20 |
| | R2* | 0.56 | 0.0003 | 25.44 | 20 |
| **IRON MOBILIZATION** | R1 | 0.0085 | 7.39E-01 | 0.17 | 20 |
| | r1-r2* relaxivity | 0.620 | 7.29E-05 | 32.7 | 20 |
| | R2* | 0.126 | 0.17 | 2.88 | 20 |

***Sup. Table 2: Statistical analysis for the results presented in figure 4.***

## Supplementary Figure 38

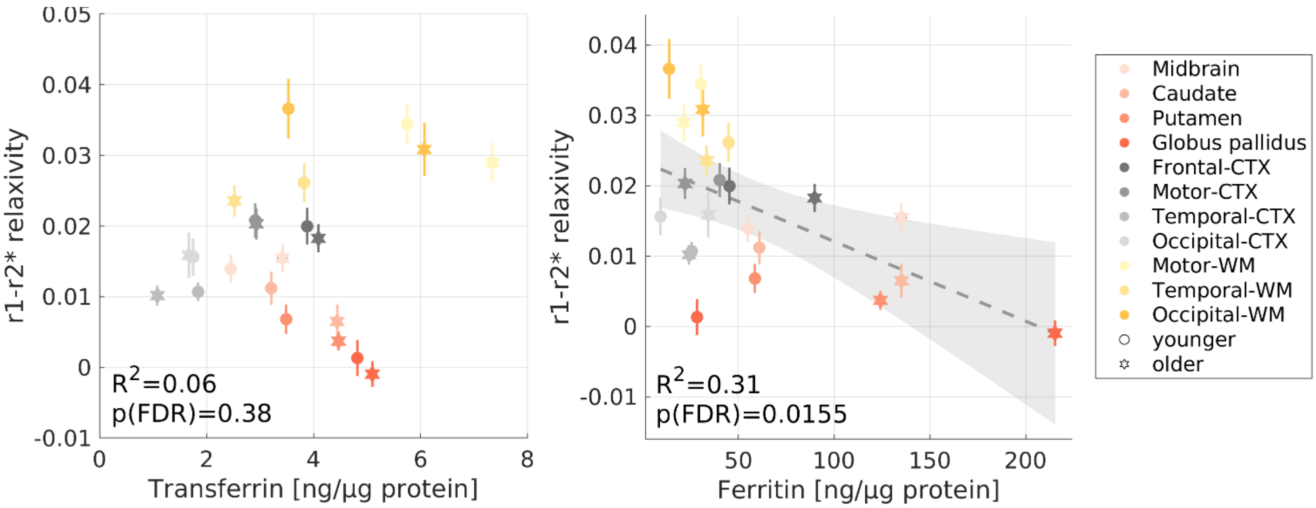


***Sup. Figure 38: The correlations of the $r_1$-$r_2^*$ relaxivity with the transferrin and ferritin concentrations.*** *The transferrin and ferritin concentrations (postmortem, from the literature[16–18]) in different brain regions of younger (aged 27-64 years, N>=7) and older (aged 65-88 years, N>=8) subjects vs. the $r_1$-$r_2^*$ relaxivity measured in vivo across younger (aged 23-63 years, N =26) and older (aged 65-77 years, N=13) subjects (different marker shapes) in 10 brain regions (different colors). For all panels, data points show mean values and error bars show the mean absolute deviation across subjects, p-values are for one-sided F-test corrected for multiple comparisons (FDR). Precise values of the sample sizes for the postmortem concentrations from the literature are in table 2.*

# Supplementary Section 12 (Supplementary Figure 39)

# The r1-r2* relaxivity in the pallidum.

The pallidum is unique in terms of its paramagnetic properties: it is highly rich in iron, but also contains iron oxides and metal depositions[3,28,29] which might affect the measurement of the $r_1$-$r_2^*$ relaxivity.

For young subjects, $R_1$ and $R_2^*$ values in the pallidum were the highest among all regions tested, and the $r_1$-$r_2^*$ relaxivity was the lowest (Figure 3). To make sure our results are not driven by the outlier values in the pallidum, we tried to exclude it from the comparisons between MRI and iron histology we show in Figure 4b-c. The correlation of the $r_1$-$r_2^*$ relaxivity with the iron concentration did not survive after excluding the pallidum (Sup. Figure 39). On the contrary, the correlations of $R_2^*$ with the iron concentration and of the $r_1$-$r_2^*$ relaxivity with the iron mobilization remained significant even when the pallidum was excluded from the analysis (Sup. Figure 39).

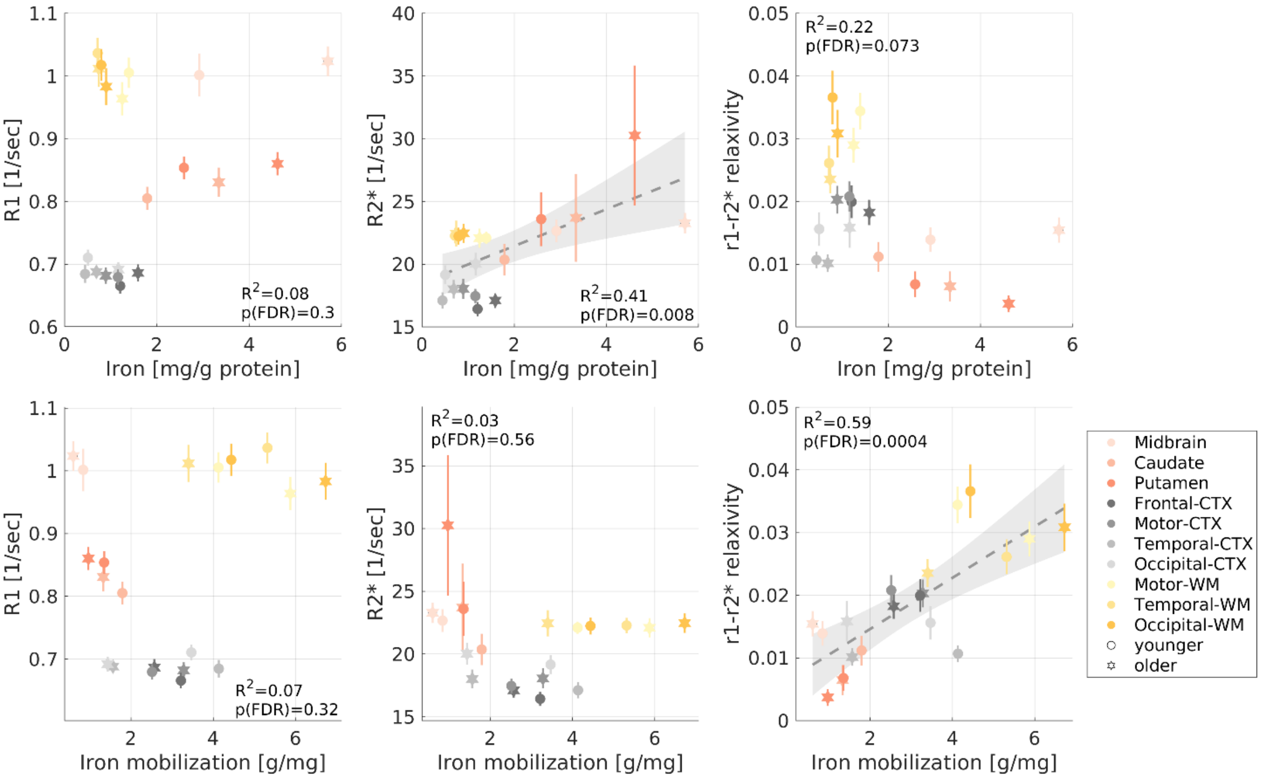


***Sup. Figure 39: The correlations of MRI parameters with the iron environment when excluding the pallidum.*** *Replication of Figure 5b-c excluding the globus pallidus. The iron concentration and the iron*

*mobilization capacity (transferrin/iron ratio) estimate postmortem (from the literature[16–18]) in different brain regions of younger (aged 27-64 years, N>=7) and older (aged 65-88 years, N>=8) subjects vs. $R_1$, $R_2^*$ and the $r_1$-$r_2^*$ relaxivity measured in vivo across younger (aged 23-63 years, N =26) and older (aged 65-77 years, N=13) subjects (different marker shapes) in 10 brain regions (different colors), excluding the pallidum. The correlation of the $r_1$-$r_2^*$ relaxivity with the iron concentration did not survive after excluding the pallidum. On the contrary, the correlations of $R_2^*$ with the iron concentration and of the $r_1$-$r_2^*$ relaxivity with the iron mobilization remained significant. Precise values of the sample sizes for the postmortem concentrations from the literature are in table 2. For all panels, data points show mean values and error bars show the mean absolute deviation across subjects, p-values are for one-sided F-test corrected for multiple comparisons (FDR).*

Therefore, the correlation of the $r_1$-$r_2^*$ relaxivity with the iron concentration was driven mostly by the distinct behavior of the pallidum, while its correlation with the iron mobilization was stronger and more stable. These analyses demonstrate the enhanced sensitivity of the r1-r2* relaxivity to the iron homeostasis rather than to the absolute iron concentration.

## Supplementary Table 3

| | | p-value | CI low | CI high | t-statistics | df | effect size |
|---|---|---|---|---|---|---|---|
| **r1-r2* relaxivity** | GM-Tumor | 7.03E-06 | -0.01 | -0.005 | -6.37 | 17 | 1.50 |
| | WM-Tumor | 1.24E-12 | -0.03 | -0.024 | -18.32 | 17 | 4.32 |
| | WM-GM | 7.05E-11 | -0.02 | -0.0167 | -14.24 | 17 | 3.36 |
| **R2*** | GM-Tumor | 0.67 | -3.89 | 5.92 | 0.435 | 17 | 0.10 |
| | WM-Tumor | 0.39 | -6.87 | 2.83 | -0.88 | 17 | 0.21 |
| | WM-GM | 1.19E-11 | -3.4 | -2.63 | -15.9 | 17 | 3.75 |
| **R1** | GM-Tumor | 0.084 | -0.09 | 0.0066 | -1.83 | 17 | 0.43 |
| | WM-Tumor | 4.27E-11 | -0.39 | -0.29 | -14.7 | 17 | 3.46 |
| | WM-GM | 9.86E-22 | -0.30 | -0.285 | -64.22 | 17 | 15.14 |
| **Gd-enhanced** | GM-Tumor | 2.77E-05 | -503.5 | -233.55 | -5.8 | 16 | 1.40 |
| | WM-Tumor | 0.0002 | -449.4 | -177.3 | -4.9 | 16 | 1.18 |
| | WM-GM | 1.14E-11 | 48.28 | 62.0 | 17.0 | 16 | 4.13 |

***Sup. Table 3: Statistical analysis for the results presented in figure 5.***

## Supplementary Figure 40

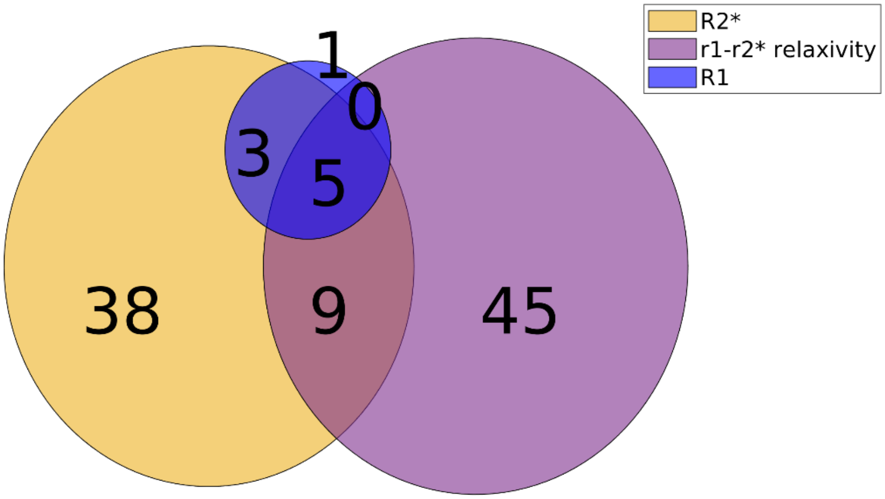


***Sup. Figure 40: The number of significantly enriched pathways associated with each qMRI parameter.*** *The Venn diagram shows the number of significantly enriched pathways (p(FWER)<0.01, p-values are for the two-sided permutation test and are corrected for multiple comparisons with familywise-error rate) for each qMRI parameter ($R_2^*$, $R_1$ and the $r_1$-$r_2^*$ relaxivity). Almost half of the significantly enriched pathways are exclusive for the $r_1$-$r_2^*$ relaxivity. See also supplementary table 4.*

# Supplementary Table 4

| pathway | R1 NES | R1 pval | R2* NES | R2* pval | r1-r2* relaxivity NES | r1-r2* relaxivity pval |
|---|---|---|---|---|---|---|
| GO ALPHA BETA T CELL ACTIVATION | 1.15 | 1 | 2.43 | 0.001 | -1.12 | 1 |
| GO ALPHA BETA T CELL DIFFERENTIATION | 1.26 | 1 | 2.44 | 0.001 | -1.04 | 1 |
| GO ANTIGEN BINDING | 0.79 | 1 | 2.23 | 0.024 | -2.86 | 0 |
| GO B CELL MEDIATED IMMUNITY | -0.64 | 1 | 1.48 | 1 | -2.56 | 0 |
| GO B CELL RECEPTOR SIGNALING PATHWAY | 0.97 | 1 | 2.21 | 0.038 | -2.50 | 0 |
| GO CD4 POSITIVE ALPHA BETA T CELL ACTIVATION | 0.99 | 1 | 2.32 | 0.005 | -1.22 | 1 |
| GO CD4 POSITIVE ALPHA BETA T CELL DIFFERENTIATION | 1.22 | 1 | 2.38 | 0.003 | -1.20 | 1 |
| GO COMPLEMENT ACTIVATION | -0.86 | 1 | 1.33 | 1 | -2.90 | 0 |
| GO CONDENSED NUCLEAR CHROMOSOME CENTROMERIC REGION | -2.33 | 0.004 | -1.64 | 1 | -1.46 | 1 |
| GO COTRANSLATIONAL PROTEIN TARGETING TO MEMBRANE | 2.55 | 0.001 | 2.44 | 0.001 | 3.18 | 0 |
| GO CYTOSOLIC LARGE RIBOSOMAL SUBUNIT | 2.31 | 0.019 | 2.27 | 0.012 | 2.95 | 0 |
| GO CYTOSOLIC RIBOSOME | 2.21 | 0.112 | 2.57 | 0 | 3.06 | 0 |
| GO CYTOSOLIC SMALL RIBOSOMAL SUBUNIT | 1.73 | 1 | 2.38 | 0.003 | 2.44 | 0.003 |
| GO DEFENSE RESPONSE TO BACTERIUM | 0.78 | 1 | 1.76 | 1 | -2.27 | 0.006 |
| GO ESTABLISHMENT OF PROTEIN LOCALIZATION TO ENDOPLASMIC RETICULUM | 2.11 | 0.317 | 2.08 | 0.272 | 3.14 | 0 |
| GO FC RECEPTOR MEDIATED STIMULATORY SIGNALING PATHWAY | 1.29 | 1 | 1.65 | 1 | -2.29 | 0.004 |
| GO HUMORAL IMMUNE RESPONSE | 0.72 | 1 | 1.72 | 1 | -2.43 | 0 |
| GO HUMORAL IMMUNE RESPONSE MEDIATED BY CIRCULATING IMMUNOGLOBULIN | -0.52 | 1 | 1.61 | 1 | -3.10 | 0 |
| GO IMMUNE RECEPTOR ACTIVITY | 1.27 | 1 | 2.46 | 0 | 0.71 | 1 |
| GO IMMUNOGLOBULIN COMPLEX | -1.14 | 1 | 2.52 | 0 | -3.62 | 0 |
| GO IMMUNOGLOBULIN COMPLEX CIRCULATING | 0.94 | 1 | 2.32 | 0.005 | -3.14 | 0 |
| GO IMMUNOGLOBULIN RECEPTOR BINDING | 0.72 | 1 | 2.30 | 0.008 | -3.06 | 0 |
| GO KINETOCHORE | -2.00 | 0.594 | -2.40 | 0.002 | -0.94 | 1 |
| GO LARGE RIBOSOMAL SUBUNIT | 0.91 | 1 | 1.13 | 1 | 2.59 | 0 |
| GO METAPHASE ANAPHASE TRANSITION OF CELL CYCLE | -2.10 | 0.219 | -2.55 | 0 | -0.81 | 1 |
| GO MITOCHONDRIAL GENE EXPRESSION | -2.18 | 0.067 | -2.45 | 0 | 1.57 | 1 |
| GO MITOCHONDRIAL TRANSLATION | -2.08 | 0.266 | -2.44 | 0 | 1.82 | 0.998 |
| GO MITOCHONDRIAL TRANSLATIONAL TERMINATION | -2.05 | 0.388 | -2.58 | 0 | 1.73 | 1 |
| GO MITOTIC METAPHASE PLATE CONGRESSION | -1.80 | 1 | -2.41 | 0.002 | 1.05 | 1 |
| GO MITOTIC NUCLEAR DIVISION | -2.12 | 0.156 | -2.32 | 0.009 | -0.85 | 1 |
| GO MITOTIC SISTER CHROMATID SEGREGATION | -2.19 | 0.059 | -2.36 | 0.005 | 0.85 | 1 |
| GO NEGATIVE REGULATION OF CHROMOSOME SEGREGATION | -2.01 | 0.548 | -2.35 | 0.007 | -0.84 | 1 |
| GO NEGATIVE REGULATION OF METAPHASE ANAPHASE TRANSITION OF CELL CYCLE | -2.13 | 0.155 | -2.46 | 0 | -0.98 | 1 |
| GO NUCLEAR TRANSCRIBED MRNA CATABOLIC PROCESS NONSENSE MEDIATED DECAY | 2.25 | 0.059 | 2.36 | 0.003 | 2.49 | 0.001 |
| GO PHAGOCYTOSIS RECOGNITION | -0.69 | 1 | 1.60 | 1 | -2.89 | 0 |
| GO POSITIVE REGULATION OF B CELL ACTIVATION | -0.51 | 1 | 1.50 | 1 | -2.35 | 0.001 |
| GO POSITIVE REGULATION OF LEUKOCYTE CELL CELL ADHESION | 0.91 | 1 | 2.32 | 0.003 | -1.06 | 1 |
| GO POSITIVE T CELL SELECTION | 1.14 | 1 | 2.45 | 0.001 | 0.92 | 1 |
| GO PROTEIN LOCALIZATION TO ENDOPLASMIC RETICULUM | 2.16 | 0.195 | 1.98 | 0.711 | 2.93 | 0 |
| GO PROTEIN TARGETING TO MEMBRANE | 1.83 | 0.998 | 1.71 | 1 | 2.50 | 0.001 |
| GO REGULATION OF B CELL ACTIVATION | 0.68 | 1 | 1.84 | 0.995 | -2.29 | 0.004 |
| GO REGULATION OF CHROMOSOME SEPARATION | -2.06 | 0.34 | -2.49 | 0 | -0.84 | 1 |
| GO REGULATION OF HUMORAL IMMUNE RESPONSE | -1.03 | 1 | 1.24 | 1 | -2.59 | 0 |
| GO REGULATION OF SISTER CHROMATID SEGREGATION | -1.94 | 0.878 | -2.43 | 0 | -0.80 | 1 |
| GO RIBOSOMAL SUBUNIT | 0.93 | 1 | 1.41 | 1 | 2.79 | 0 |
| GO RIBOSOME | -0.80 | 1 | 1.25 | 1 | 2.64 | 0 |
| GO SMALL RIBOSOMAL SUBUNIT | -0.91 | 1 | 1.43 | 1 | 2.40 | 0.007 |
| GO STRUCTURAL CONSTITUENT OF RIBOSOME | 1.07 | 1 | 1.49 | 1 | 2.91 | 0 |
| GO TRANSLATIONAL INITIATION | 1.93 | 0.951 | 2.05 | 0.401 | 2.47 | 0.001 |
| GO TRANSLATIONAL TERMINATION | -1.98 | 0.68 | -2.51 | 0 | 1.83 | 0.997 |
| GO T CELL ACTIVATION INVOLVED IN IMMUNE RESPONSE | 1.27 | 1 | 2.46 | 0 | -1.22 | 1 |
| GO T CELL RECEPTOR COMPLEX | -0.60 | 1 | 2.35 | 0.003 | 0.65 | 1 |
| GO T CELL SELECTION | 1.21 | 1 | 2.58 | 0 | 1.06 | 1 |
| GO UNFOLDED PROTEIN BINDING | -1.08 | 1 | -2.34 | 0.007 | 1.36 | 1 |
| KEGG LEISHMANIA INFECTION | 1.70 | 1 | 2.44 | 0.001 | 1.01 | 1 |
| KEGG RIBOSOME | 2.53 | 0.001 | 2.68 | 0 | 3.10 | 0 |
| PID IL12 2PATHWAY | 0.99 | 1 | 2.35 | 0.003 | -1.04 | 1 |
| PID PLK1 PATHWAY | -2.61 | 0 | -2.38 | 0.003 | -1.27 | 1 |
| PID TCR PATHWAY | 0.81 | 1 | 2.37 | 0.003 | -0.94 | 1 |
| REACTOME ACTIVATION OF THE MRNA UPON BINDING OF THE CAP BINDING COMPLEX AND EIFS AND SUBSEQUENT BINDING TO | 2.26 | 0.056 | 2.23 | 0.022 | 2.75 | 0 |
| REACTOME ANTIGEN ACTIVATES B CELL RECEPTOR BCR LEADING TO GENERATION OF SECOND MESSENGERS | 0.80 | 1 | 1.91 | 0.947 | -3.01 | 0 |
| REACTOME BINDING AND UPTAKE OF LIGANDS BY SCAVENGER RECEPTORS | -0.75 | 1 | 1.58 | 1 | -2.74 | 0 |
| REACTOME CD22 MEDIATED BCR REGULATION | -1.40 | 1 | 2.18 | 0.073 | -3.25 | 0 |
| REACTOME CELL CYCLE CHECKPOINTS | -2.17 | 0.073 | -2.54 | 0 | -1.00 | 1 |
| REACTOME COMPLEMENT CASCADE | -1.04 | 1 | 0.94 | 1 | -2.78 | 0 |
| REACTOME CREATION OF C4 AND C2 ACTIVATORS | -0.99 | 1 | 1.52 | 1 | -3.07 | 0 |
| REACTOME CYCLIN A B1 B2 ASSOCIATED EVENTS DURING G2 M TRANSITION | -2.34 | 0.004 | -2.45 | 0 | -0.97 | 1 |
| REACTOME EUKARYOTIC TRANSLATION ELONGATION | 2.67 | 0 | 2.80 | 0 | 3.19 | 0 |
| REACTOME EUKARYOTIC TRANSLATION INITIATION | 2.56 | 0.001 | 2.60 | 0 | 3.23 | 0 |
| REACTOME FCERI MEDIATED CA 2 MOBILIZATION | 1.18 | 1 | 2.11 | 0.172 | -2.91 | 0 |
| REACTOME FCERI MEDIATED MAPK ACTIVATION | 0.89 | 1 | 1.78 | 1 | -2.88 | 0 |
| REACTOME FCGAMMA RECEPTOR FCGR DEPENDENT PHAGOCYTOSIS | 1.40 | 1 | 1.61 | 1 | -2.41 | 0 |
| REACTOME FCGR3A MEDIATED IL10 SYNTHESIS | 0.76 | 1 | 1.93 | 0.891 | -2.86 | 0 |
| REACTOME FCGR ACTIVATION | 0.97 | 1 | 2.01 | 0.558 | -3.27 | 0 |
| REACTOME GENERATION OF SECOND MESSENGER MOLECULES | 1.34 | 1 | 2.57 | 0 | 1.17 | 1 |
| REACTOME IMMUNOREGULATORY INTERACTIONS BETWEEN A LYMPHOID AND A NON LYMPHOID CELL | 0.52 | 1 | 2.39 | 0.003 | -2.21 | 0.02 |
| REACTOME INFLUENZA INFECTION | 1.42 | 1 | 1.79 | 1 | 2.70 | 0 |
| REACTOME INITIAL TRIGGERING OF COMPLEMENT | -0.86 | 1 | 1.42 | 1 | -3.05 | 0 |
| REACTOME INTERLEUKIN 10 SIGNALING | 0.72 | 1 | 1.92 | 0.929 | -2.45 | 0 |
| REACTOME MITOCHONDRIAL TRANSLATION | -2.06 | 0.339 | -2.48 | 0 | 1.80 | 1 |
| REACTOME MITOTIC METAPHASE AND ANAPHASE | -1.99 | 0.624 | -2.46 | 0 | 1.20 | 1 |
| REACTOME MITOTIC PROMETAPHASE | -1.89 | 0.969 | -2.34 | 0.007 | -0.83 | 1 |
| REACTOME MITOTIC SPINDLE CHECKPOINT | -2.21 | 0.042 | -2.69 | 0 | -0.71 | 1 |
| REACTOME NONSENSE MEDIATED DECAY NMD | 2.28 | 0.039 | 2.42 | 0.001 | 2.66 | 0 |
| REACTOME PARASITE INFECTION | 0.90 | 1 | 1.39 | 1 | -2.59 | 0 |
| REACTOME REGULATION OF EXPRESSION OF SLITS AND ROBOS | 1.49 | 1 | 1.83 | 0.999 | 2.52 | 0.001 |
| REACTOME RESOLUTION OF D LOOP STRUCTURES THROUGH SYNTHESIS DEPENDENT STRAND ANNEALING SDSA | -2.15 | 0.101 | -2.43 | 0 | -1.38 | 1 |
| REACTOME RESOLUTION OF SISTER CHROMATID COHESION | -2.31 | 0.005 | -2.71 | 0 | -0.72 | 1 |
| REACTOME RESPONSE OF EIF2AK4 GCN2 TO AMINO ACID DEFICIENCY | 2.21 | 0.111 | 2.55 | 0 | 2.73 | 0 |
| REACTOME ROLE OF LAT2 NTAL LAB ON CALCIUM MOBILIZATION | 1.12 | 1 | 1.94 | 0.852 | -3.13 | 0 |
| REACTOME ROLE OF PHOSPHOLIPIDS IN PHAGOCYTOSIS | 1.41 | 1 | 2.25 | 0.016 | -3.04 | 0 |
| REACTOME RRNA PROCESSING | 0.91 | 1 | 1.32 | 1 | 2.69 | 0 |
| REACTOME SCAVENGING OF HEME FROM PLASMA | 1.37 | 1 | 2.20 | 0.038 | -3.27 | 0 |
| REACTOME SELENOAMINO ACID METABOLISM | 1.87 | 0.993 | 2.31 | 0.008 | 2.95 | 0 |
| REACTOME SEPARATION OF SISTER CHROMATIDS | -2.05 | 0.363 | -2.54 | 0 | 1.10 | 1 |
| REACTOME SIGNALING BY ROBO RECEPTORS | 1.61 | 1 | 1.90 | 0.961 | 2.40 | 0.007 |
| REACTOME SRP DEPENDENT COTRANSLATIONAL PROTEIN TARGETING TO MEMBRANE | 2.30 | 0.027 | 2.24 | 0.021 | 3.14 | 0 |
| REACTOME TRANSLATION | -1.14 | 1 | -1.34 | 1 | 2.93 | 0 |
| WP CYTOPLASMIC RIBOSOMAL PROTEINS | 2.71 | 0 | 2.77 | 0 | 3.17 | 0 |
| WP MICROGLIA PATHOGEN PHAGOCYTOSIS PATHWAY | 1.79 | 0.999 | 2.61 | 0 | 1.63 | 1 |
| WP TYROBP CAUSAL NETWORK | 2.30 | 0.024 | 2.83 | 0 | 1.16 | 1 |

***Sup. Table 4: Significantly enriched pathways for $R_1$, $R_2^*$ and the $r_1$-$r_2^*$ relaxivity.*** *For each of the 101 gene sets, we show the normalized enrichments score (NES) for each of the three qMRI parameters, along with the p-values (for the two-sided permutation test corrected for multiple comparisons with familywise-error rate). This table was used for the clustering shown in Figure 6b.*

## Supplementary Figure 41

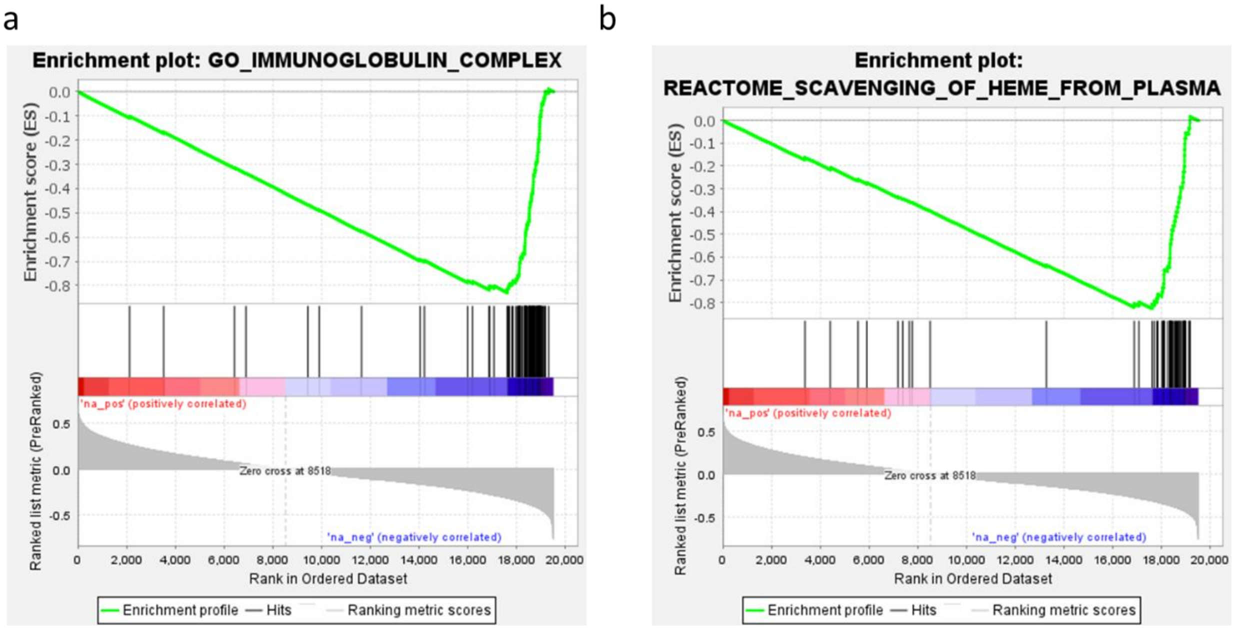


***Sup. Figure 41: Enrichment plots of the two most enriched pathways for the $r_1$-$r_2^*$ relaxivity.*** *Gene enrichment plots of the two most enriched pathways for the $r_1$-$r_2^*$ relaxivity "Immunoglobulin complex"* ***(a)*** *and "scavenging of heme from plasma"* ***(b)****. The top portion of each panel shows the running enrichment score for the gene set as the analysis goes over the ranked list of genes. The list is based on the genes' correlation with the $r_1$-$r_2^*$ relaxivity. The middle portion of each panel shows where the members of the gene set appear in the ranked list of genes. The bottom portion of each panel shows the r value of the correlation between genes and the $r_1$-$r_2^*$ relaxivity. The two gene sets preferentially fall toward the negative end of the correlation spectrum, indicating their significant association with the $r_1$-$r_2^*$ relaxivity.*

# Supplementary methods

### $R_1$-MTV dependency computation for phantoms.

We computed the linear dependency of $R_1$ on MTV across samples with varying iron-binding protein concentrations and liposomal fractions[13]. This process was implemented in MATLAB. We extracted the MTV values from all voxels and pooled them into 12 bins spaced equally between 0.05 and 0.40. This was done so that the linear fit would not be heavily affected by the density of the samples in different MTV regimes. The median MTV of each bin was computed, along with the median $R_1$. We fitted the following linear model across samples:

$$R1 = a * MTV + b$$

The slope of this linear model ($a$) represents the $R_1$-MTV dependency. $b$ is constant.

# Supplementary references


1. Donahue, K. M., Weisskoff, R. M. & Burstein, D. Water diffusion and exchange as they influence contrast enhancement. *J. Magn. Reson. Imaging* **7**, 102–110 (1997).

2. Rooney, W. D. *et al.* Magnetic field and tissue dependencies of human brain longitudinal1H2O relaxation in vivo. *Magn. Reson. Med.* **57**, 308–318 (2007).

3. Haacke, E. M. *et al.* Imaging iron stores in the brain using magnetic resonance imaging. *Magn. Reson. Imaging* **23**, 1–25 (2005).

4. Möller, H. E. *et al.* Iron, Myelin, and the Brain: Neuroimaging Meets Neurobiology. *Trends Neurosci.* (2019).

5. Blockley, N. P. *et al.* Field strength dependence of R1 and R relaxivities of human whole blood to prohance, vasovist, and deoxyhemoglobin. *Magn. Reson. Med.* **60**, 1313–1320 (2008).

6. Copen, W. A., Lev, M. H. & Rapalino, O. Brain perfusion: computed tomography and magnetic resonance techniques. *Handb. Clin. Neurol.* **135**, 117–135 (2016).

7. Gelman, N., Ewing, J. R., Gorell, J. M., Spickler, E. M. & Solomon, E. G. Interregional variation of

longitudinal relaxation rates in human brain at 3.0 T: Relation to estimated iron and water contents. *Magn. Reson. Med.* (2001).

8. Callaghan, M. F. *et al.* Widespread age-related differences in the human brain microstructure revealed by quantitative magnetic resonance imaging. *Neurobiol. Aging* **35**, 1862–1872 (2014).

9. Lorio, S. *et al.* Disentangling in vivo the effects of iron content and atrophy on the ageing human brain. *Neuroimage* **103**, 280–289 (2014).

10. Weiskopf, N., Mohammadi, S., Lutti, A. & Callaghan, M. F. Advances in MRI-based computational neuroanatomy. *Curr. Opin. Neurol.* **28**, 313–322 (2015).

11. Heath, F., Hurley, S. A., Johansen-Berg, H. & Sampaio-Baptista, C. Advances in noninvasive myelin imaging. *Dev. Neurobiol.* **78**, 136–151 (2018).

12. Lutti, A., Dick, F., Sereno, M. I. & Weiskopf, N. Using high-resolution quantitative mapping of R1 as an index of cortical myelination. *Neuroimage* **93**, 176–188 (2014).

13. Filo, S. *et al.* Disentangling molecular alterations from water-content changes in the aging human brain using quantitative MRI. *Nat. Commun.* **10**, (2019).

14. Kiselev, V. G. & Novikov, D. S. Transverse NMR relaxation in biological tissues. *NeuroImage* vol. 182 149–168 (2018).

15. Kirilina, E. *et al.* Superficial white matter imaging: Contrast mechanisms and whole-brain in vivo mapping. *Sci. Adv.* **6**, aaz9281 (2020).

16. Connor, J. R., Snyder, B. S., Arosio, P., Loeffler, D. A. & LeWitt, P. A Quantitative Analysis of Isoferritins in Select Regions of Aged, Parkinsonian, and Alzheimer's Diseased Brains. *J. Neurochem.* **65**, 717–724 (1995).

17. Loeffler, D. A. *et al.* Transferrin and Iron in Normal, Alzheimer's Disease, and Parkinson's Disease Brain Regions. *J. Neurochem.* **65**, 710–716 (1995).

18. Connor, J. R., Snyder, B. S., Beard, J. L., Fine, R. E. & Mufson, E. J. Regional distribution of iron and iron-regulatory proteins in the brain in aging and Alzheimer's disease. *J. Neurosci. Res.* **31**, 327–

335 (1992).

19. Mezer, A. *et al.* Quantifying the local tissue volume and composition in individual brains with magnetic resonance imaging. *Nat. Med.* **19**, 1667–72 (2013).

20. Mohammadi, S. & Callaghan, M. F. Towards in vivo g-ratio mapping using MRI: Unifying myelin and diffusion imaging. *J. Neurosci. Methods* **348**, 108990 (2021).

21. Berman, S., West, K. L., Does, M. D., Yeatman, J. D. & Mezer, A. A. Evaluating g-ratio weighted changes in the corpus callosum as a function of age and sex. *Neuroimage* **182**, 304–313 (2018).

22. Mancini, M. *et al.* An interactive meta-analysis of MRI biomarkers of Myelin. *Elife* **9**, 1–23 (2020).

23. Piredda, G. F., Hilbert, T., Thiran, J. & Kober, T. Probing myelin content of the human brain with MRI: A review. *Magn. Reson. Med.* **85**, 627–652 (2021).

24. Lévy, S. *et al.* Test-retest reliability of myelin imaging in the human spinal cord: Measurement errors versus region- and aging-induced variations. *PLoS One* **13**, (2018).

25. Stüber, C. *et al.* Myelin and iron concentration in the human brain: A quantitative study of MRI contrast. *Neuroimage* **93**, 95–106 (2014).

26. Callaghan, M. F., Helms, G., Lutti, A., Mohammadi, S. & Weiskopf, N. A general linear relaxometry model of R1 using imaging data. *Magn. Reson. Med.* **73**, 1309–14 (2015).

27. Glasser, M. F. & van Essen, D. C. Mapping Human Cortical Areas In Vivo Based on Myelin Content as Revealed by T1- and T2-Weighted MRI. *J. Neurosci.* **31**, 11597–11616 (2011).

28. Dlháň, Ľ., Kopáni, M. & Boča, R. Magnetic properties of iron oxides present in the human brain. *Polyhedron* **157**, 505–510 (2019).

29. Erikson, K. M., Syversen, T., Steinnes, E. & Aschner, M. Globus pallidus: A target brain region for divalent metal accumulation associated with dietary iron deficiency. *J. Nutr. Biochem.* **15**, 335–341 (2004).
