# Supplementary material for: Non-invasive assessment of normal and impaired iron homeostasis in the brain
Source: Nat Commun. 2023 Sep 12;14:5467. doi: 10.1038/s41467-023-40999-z (PMC10497590; doi:10.1038/s41467-023-40999-z)
Supplement: Supplementary file 3 — Reporting Summary [file 41467_2023_40999_MOESM3_ESM.pdf]

nature portfolio

Corresponding author(s): Shir Filo

Last updated by author(s): Aug 15, 2023

Reporting Summary

Nature Portfolio wishes to improve the reproducibility of the work that we publish. This form provides structure for consistency and transparency in reporting. For further information on Nature Portfolio policies, see our Editorial Policies and the Editorial Policy Checklist.

Statistics

For all statistical analyses, confirm that the following items are present in the figure legend, table legend, main text, or Methods section.

n/a	Confirmed	
☐	☒	The exact sample size (*n*) for each experimental group/condition, given as a discrete number and unit of measurement
☐	☒	A statement on whether measurements were taken from distinct samples or whether the same sample was measured repeatedly
☐	☒	The statistical test(s) used AND whether they are one- or two-sided<br>*Only common tests should be described solely by name; describe more complex techniques in the Methods section.*
☐	☒	A description of all covariates tested
☐	☒	A description of any assumptions or corrections, such as tests of normality and adjustment for multiple comparisons
☐	☒	A full description of the statistical parameters including central tendency (e.g. means) or other basic estimates (e.g. regression coefficient) AND variation (e.g. standard deviation) or associated estimates of uncertainty (e.g. confidence intervals)
☐	☒	For null hypothesis testing, the test statistic (e.g. *F*, *t*, *r*) with confidence intervals, effect sizes, degrees of freedom and *P* value noted<br>*Give P values as exact values whenever suitable.*
☐	☒	For Bayesian analysis, information on the choice of priors and Markov chain Monte Carlo settings
☐	☒	For hierarchical and complex designs, identification of the appropriate level for tests and full reporting of outcomes
☐	☒	Estimates of effect sizes (e.g. Cohen's *d*, Pearson's *r*), indicating how they were calculated

Our web collection on statistics for biologists contains articles on many of the points above.

Software and code

Policy information about availability of computer code

Data collection	Healthy Human subjects data was collected on a 3T Siemens MAGNETOM Skyra scanner equipped with a 32-channel head receive-only coil at the ELSC neuroimaging unit at the Hebrew University. Human measurements were performed on 26 young adults (aged 27 ± 10 years, 10 females), and 13 older adults (aged 70 ± 3 years, 4 females). The Helsinki Ethics Committee of Hadassah Hospital, Jerusalem, Israel approved the experimental procedure. Written informed consent was obtained from each participant prior to the procedure.<br>Meningioma patients data was collected on a 3T Siemens MAGNETOM Skyra scanner equipped with a 32-channel head receive-only coil at the Shaare Zedek Medical Center. MRI measurements were performed on 19 patients. Brain meningioma surgical specimens, available for 16 patients, were obtained from the fresh frozen tissue biobank of the Department of Neurosurgery, Shaare Zedek Medical Center, Jerusalem, Israel, and were transferred on dry ice for western-blot and gene expression analyses. Study participants provided informed consent according to an institutional review board.
Data analysis	The software described in the paper for calculating r1-r2* relaxivities was developed on Matlab 2017b.<br>It is publicly available, including example data (https://github.com/shirfilo/r1_r2s_rel_toolbox).<br>R1 and R2* maps were generated using the following softwares:<br>1) mrQ v2.1 (https://github.com/mezera/mrQ)<br>2) vistasoft v1.0 (https://github.com/vistalab/vistasoft/wiki)<br>3) Voxel Based Quantification (VBQ) toolbox (v2e) in SMP12.<br>4) Freesurfer v6.0 (https://surfer.nmr.mgh.harvard.edu/)<br><br>Gene expression analysis was done using the following software:<br>1) Illumina's bcl2fastq software (v2.19.1.403)

March 2021

2) HTseq (v 0.13.5)
3) RNAseQC software (Picard v2.26.10)
4) DEseq2 R package from Bioconductor (v3.13)
5) GSEA software (v4.1.0)
6) STAR (v2.7.3a)
Tumor contouring was performed by the neurosurgeon (T.S.) using BrainLab's Elements software, Cranial Navigation 3.1.5 (BrainLab AG, Munich, Germany)
Blot intensities were quantified using the FIJI ImageJ software (v2.35)

For manuscripts utilizing custom algorithms or software that are central to the research but not yet described in published literature, software must be made available to editors and reviewers. We strongly encourage code deposition in a community repository (e.g. GitHub). See the Nature Portfolio guidelines for submitting code & software for further information.

Data

Policy information about availability of data

All manuscripts must include a data availability statement. This statement should provide the following information, where applicable:
- Accession codes, unique identifiers, or web links for publicly available datasets
- A description of any restrictions on data availability
- For clinical datasets or third party data, please ensure that the statement adheres to our policy

The raw and processed RNA-sequencing data generated in this study have been deposited in the GEO database under accession code GSE240204 [https://www.ncbi.nlm.nih.gov/geo/query/acc.cgi?acc=GSE240204]. MRI and proteomics measurements for each Meningioma patient, and MRI measurements for healthy subjects at all presented ROIs are available in the source data file. The raw MRI data are not publicly available due to them containing information that could compromise research participant privacy/consent. Anonymized images and any additional raw data are available from the corresponding author [S.F.] upon request which conform with the privacy guidelines of the Hadassah Hospital and Shaare Zedek Medical Center Helsinki Ethics Committees. The timeframe for response to such requests is within a month. Source data are provided with this paper.

Human research participants

Policy information about studies involving human research participants and Sex and Gender in Research.

Reporting on sex and gender	We collected MRI data of both males and females (see below). Sex and gender are irrelevant for the findings of our study.
Population characteristics	Healthy Human subjects data was collected for 26 young adults (aged 27 ± 10 years, 10 females), and 13 older adults (aged 70 ± 3 years, 4 females).<br>Meningioma MRI data was collected for 19 patients. Brain meningioma surgical specimens were collected for 16 of these patients.
Recruitment	Healthy participants were recruited through printed and electronic advertisements. The recruitment process might be biased towards volunteers from the community surrounding the university. However, as this is a structural brain study this bias is unlikely to impact the results. Meningioma patients were recruited in Shaare Zedek Medical Center upon diagnosis. The Helsinki Ethics Committees of Hadassah Hospital and Shaare Zedek Medical Center, Jerusalem, Israel approved the experimental procedure. Written informed consent was obtained from each participant prior to the procedure.
Ethics oversight	The Helsinki Ethics Committee of Hadassah Hospital, Jerusalem, Israel.<br>The Helsinki Ethics Committee of Shaare Zedek Medical Center, Jerusalem, Israel.

Note that full information on the approval of the study protocol must also be provided in the manuscript.

Field-specific reporting

Please select the one below that is the best fit for your research. If you are not sure, read the appropriate sections before making your selection.

[x] Life sciences   [ ] Behavioural & social sciences   [ ] Ecological, evolutionary & environmental sciences

For a reference copy of the document with all sections, see nature.com/documents/nr-reporting-summary-flat.pdf

Life sciences study design

All studies must disclose on these points even when the disclosure is negative.

Sample size	Healthy Human subjects data was collected for 26 young adults (aged 27 ± 10 years, 10 females), and 13 older adults (aged 70 ± 3 years, 4 females).<br>Meningioma MRI data was collected for 19 patients. Brain meningioma surgical specimens were collected for 16 of these patients.<br><br>The sample size for this study was determined based on the number of subjects recruited by the time data analysis began. Data collection

	continued until a predefined milestone was reached, ensuring a sufficient sample size for analysis. The decision to stop data collection and start the analysis only after collecting all the data was deliberate to prevent bias in the study's findings. By doing so, we ensured that we did not repeatedly test our results until obtaining a desired outcome. The chosen sample size was taken into account in the statistical analysis, and provided adequate power for meaningful conclusions.
Data exclusions	One subject, with a titanium cranial fixation plate adjacent to the tumor, was excluded from the study due to local disruption of the magnetic field.
Replication	We successfully replicated our results on two independent MRI datasets collected at the ELSC neuroimaging unit at the Hebrew University and at the Shaare Zedek Medical Center.
Randomization	Participants were allocated to groups according to their age and medical condition.
Blinding	During MRI data collection the investigator met the participants and hence was aware of their age and medical condition. As this is a structural and not behavioral MRI study blinding is irrelevant.

Reporting for specific materials, systems and methods

We require information from authors about some types of materials, experimental systems and methods used in many studies. Here, indicate whether each material, system or method listed is relevant to your study. If you are not sure if a list item applies to your research, read the appropriate section before selecting a response.

Materials & experimental systems

n/a	Involved in the study
☐	☒ Antibodies
☒	☐ Eukaryotic cell lines
☒	☐ Palaeontology and archaeology
☒	☐ Animals and other organisms
☒	☐ Clinical data
☒	☐ Dual use research of concern

Methods

n/a	Involved in the study
☒	☐ ChIP-seq
☒	☐ Flow cytometry
☐	☒ MRI-based neuroimaging

Antibodies

Antibodies used	Anti-Ferritin Light chain (#AB69090, Abcam, 1:1,000 dilution) and Anti-Transferrin (#AB82411, Abcam, 1:10,000 dilution) primary antibodies and appropriate horseradish peroxidase-conjugated secondary antibody (#AB6721, Abcam , 1:20000 dilution).
Validation	Both primary antibodies were widely used in many high quality papers. #AB69090 has been referenced in 78 publications (see https://www.abcam.com/products/primary-antibodies/ferritin-light-chain-antibody-ab69090.html). #AB82411 has been referenced in 50 publications (see https://www.abcam.com/products/primary-antibodies/transferrin-antibody-ab82411.html).

Magnetic resonance imaging

Experimental design

Design type	As we use quantitative MRI this is not relevant.
Design specifications	As we use quantitative MRI this is not relevant.
Behavioral performance measures	As we use quantitative MRI this is not relevant.

Acquisition

Imaging type(s)	structural
Field strength	3 T
Sequence & imaging parameters	MRI Acquisition:<br>Quantitative R1, R2* & MTV mapping: 3D Spoiled gradient (SPGR) echo images were acquired with different flip angles (α = 4°, 10°, 20° and 30°). Each image included 5 equally spaced echoes (TE=3.34-14.02 ms) and the TR was 19 ms (except for 6 young subjects for which the scan included only one TE=3.34 ms). The scan resolution was 1 mm isotropic. Additional SPGR echo image was acquired with an MT pulse (TE=3.34, TR=27, α = 10, 1 mm isotropic). For B1+ mapping, we acquired additional spin-echo inversion recovery scan with an echo-planar imaging (EPI) read-out (SEIR-epi). This scan was done with a slab-inversion pulse and spatial-spectral fat suppression. For SEIR-epi, the TE/TR was 49/2920 ms. TI were 200, 400, 1,200, and 2,400 ms. We used 2-mm in-plane resolution with a slice thickness of 3 mm. The EPI readout was performed using 2× acceleration.

Anatomical images: 3D magnetization-prepared rapid gradient echo (MPRAGE) scans were acquired for 30 of the 39 healthy subjects. The scan resolution was 1 mm isotropic, the TE/TR were 2.98/2,300 ms. Magnetization-prepared 2 rapid acquisition gradient echo (MP2RAGE) scans were acquired for the remaining 9 subjects. The scan resolution was 1 mm isotropic, the TE/TR were 2.98/5,000 ms.
Whole-brain DTI measurements: performed using a diffusion-weighted spin-echo EPI sequence with isotropic 1.5-mm resolution. Diffusion weighting gradients were applied at 64 directions and the strength of the diffusion weighting was set to b = 2000 s/mm2 (TE/TR=95.80/6,000 ms, G=45 mT/m, δ=32.25 ms, Δ=52.02 ms). The data includes eight non-diffusion-weighted images (b = 0). In addition, we collected non-diffusion-weighted images with reversed phase-encode blips. For two subjects (1 young, 1 old) we failed to acquire this correction data and they were excluded from the diffusion analysis.

Gd-enhanced anatomical images: Gd-enhanced MPRAGE scans were acquired for Meningioma patients. The scan resolution was 1 mm isotropic, the TE/TR were 2.4/1,800 ms. The contrast agent was either Multihance or Dotarem at a dose of 0.1 mmol/kg. Contrast agent injection and MPRAGE acquisition were done after the acquisition of the quantitative MRI protocol, or on a different day.

Area of acquisition: Whole brain

Diffusion MRI: ☐ Used ☒ Not used

Preprocessing

Preprocessing software:
The software described in the paper for calculating r1-r2* relaxivities was developed on Matlab 2017b.
It is publicly available, including example data (https://github.com/shirfilo/r1_r2s_rel_toolbox).
R1 and R2* maps were generated using the following softwares:
1) mrQ v2.1 (https://github.com/mezera/mrQ)
2) vistasoft v1.0 (https://github.com/vistalab/vistasoft/wiki)
3) Voxel Based Quantification (VBQ) toolbox (v2e) in SMP12.
4) Freesurfer v6.0 (https://surfer.nmr.mgh.harvard.edu/)

Gene expression analysis was done using the following software:
1) Illumina's bcl2fastq software (v2.19.1.403)
2) HTseq (v 0.13.5)
3) RNAseQC software (Picard v2.26.10)
4) DEseq2 R package from Bioconductor (v3.13)
5) GSEA software (v4.1.0)
6) STAR (v2.7.3a)
Tumor contouring was performed by the neurosurgeon (T.S.) using BrainLab's Elements software, Cranial Navigation 3.1.5 (BrainLab AG, Munich, Germany)
Blot intensities were quantified using the FIJI ImageJ software (v2.35)

Normalization: Our approach is designed for the individual subject level and uses quantitative MRI, therefore no normalization was needed.

Normalization template: Our approach is designed for the individual subject level and uses quantitative MRI, therefore no normalization was needed.

Noise and artifact removal: As we use quantitative MRI this is not relevant.

Volume censoring: As we use quantitative MRI this is not relevant.

Statistical modeling & inference

Model type and settings: As we use quantitative MRI this is not relevant.

Effect(s) tested: As we use quantitative MRI this is not relevant.

Specify type of analysis: ☒ Whole brain ☐ ROI-based ☐ Both

Statistic type for inference (See Eklund et al. 2016): As we use quantitative MRI this is not relevant.

Correction: We employed multiple comparisons correction using the false discovery rate (FDR) method.

Models & analysis

n/a	Involved in the study
☒	☐ Functional and/or effective connectivity
☒	☐ Graph analysis
☒	☐ Multivariate modeling or predictive analysis
